# Retinoic acid-induced 2 deficiency impairs genomic stability in breast cancer

**DOI:** 10.1186/s13058-025-02085-8

**Published:** 2025-07-22

**Authors:** Lena Boettcher, Sarah Greimeier, Kerstin Borgmann, Shabbir S. Mughal, Bernhard Ellinger, Kai Bartkowiak, Bernd Zobiak, Antonio V. Failla, Pascal Steffen, Ellen Claus, Katharina Besler, Christopher Buccitelli, Violetta Schaaf, Ann-Kathrin Ozga, Simona Parretta, Svenja Schneegans, Wael Y. Mansour, Jan O. Korbel, Hartmut Schlueter, Benedikt Brors, Klaus Pantel, Harriet Wikman, Stefan Werner

**Affiliations:** 1 Institute of Tumour Biology, University Medical Centre Hamburg-Eppendorf, 20246 Hamburg, Germany; 2 Department of Radiotherapy and Radiooncology, University Medical Centre Hamburg-Eppendorf, 20246 Hamburg, Germany; 3 Division of Applied Bioinformatics (G330), German Cancer Research Center (DKFZ), Heidelberg, Germany; 4 National Centre for Tumour Diseases (NCT), 69120 Heidelberg, Germany; 5 Department Screening Port, Fraunhofer Institute for Translational Medicine and Pharmacology ITMP, 22525 Hamburg, Germany; 6 UKE Microscopy Imaging Facility, University Medical Centre Hamburg-Eppendorf, 20246 Hamburg, Germany; 7 Section Mass Spectrometric Proteomics, University Medical Centre Hamburg- Eppendorf, 20246 Hamburg, Germany; 8 European Molecular Biology Laboratory (EMBL), Genome Biology Unit, 69117 Heidelberg, Germany; 9 Max Delbrück Centre for Molecular Medicine, 13125 Berlin, Germany; 10 Ruprecht-Karls-Universität Heidelberg, 69117 Heidelberg, Germany; 11 Institute of Medical Biometry and Epidemiology, University Medical Centre Hamburg-Eppendorf, 20246 Hamburg, Germany; 12 Mildred Scheel Cancer Career Centre HaTriCS4, University Medical Centre Hamburg-Eppendorf, 20246 Hamburg, Germany

**Keywords:** Breast cancer, Genomic instability, Premature mitotic entry, DNA damage response, poly-(ADP-ribose)

## Abstract

**Background:**

Genome instability is a fundamental feature and hallmark of cancer, associated with aggressiveness, drug resistance and poor prognosis. RAI2 was initially identified as a novel metastasis suppressor protein specifically associated with the presence of disseminated tumour cells in the bone marrow of breast cancer patients, but its molecular function is largely unknown.

**Methods:**

We analysed the consequences of RAI2 depletion on gene expression and genomic stability in luminal breast cancer cell lines, performed cytotoxicity profiling using a library of pharmacologically active compounds, and characterized a potential function of the RAI2 protein in the DNA damage response. We performed in silico validation in different breast cancer datasets.

**Results:**

Analysis of clinical samples revealed that in primary breast tumours, low *RAI2* gene expression is significantly associated with genomically unstable tumours and poor prognosis. RAI2 depletion in breast cancer cell lines resulted in loss of mitotic fidelity characterized by prolonged mitosis with increased chromosome segregation errors and micronuclei formation. Drug screening revealed increased sensitivity of RAI2-depleted breast cancer cells to topoisomerase I and Aurora A inhibitors. We also found that genotoxic stress induces the RAI2 protein, which has an affinity for and colocalises with poly-(ADP-ribose). We validated the association of *RAI2* gene expression with DNA repair capacity in clinical samples.

**Conclusions:**

Our findings support, for the first time, a functional role of RAI2 in the maintenance of genomic stability. Understanding the underlying the molecular mechanism could help to improve patient diagnosis and treatment.

**Supplementary Information:**

The online version contains supplementary material available at 10.1186/s13058-025-02085-8.

## Background

Metastasis is a multistep process involving the hematogenous dissemination of cells from the primary tumor to distant sites such as the bone marrow, where the cells can either remain dormant as disseminated tumor cells (DTCs) or begin to proliferate and form overt metastases [1]. Due to its complex phenotype, the acquisition of metastatic competence represents a major selective barrier during cancer progression. It has been proposed that such a macroevolutionary leap leading to metastatic competence of tumor cells can only be achieved by large-scale genomic alterations leading to chromosomal instability (CIN) [2]. CIN is the predominant mechanism leading to genomic instability [3]. CIN can arise through either mitotic or premitotic defects [4] and is associated with aggressiveness, drug resistance, and poor prognosis [5, 6]. Recent evidence also suggests that genomic instability may shape the antitumor immune response [7]. Fundamental studies have confirmed that intra-tumor heterogeneity mediated by genome doubling and ongoing dynamic CIN drives disease recurrence in both renal cell and lung cancer. Interestingly, this effect was not associated with mutational burden [8, 9]. More recently, the evolution of late-stage metastatic melanoma has been shown to be dominated by aneuploidy and whole genome doubling [10].

Previously, RAI2 was identified as a novel metastasis suppressor gene associated with both breast and colorectal cancer metastasis, but its molecular function is still largely unknown [11, 12]. Low *RAI2* gene expression in primary breast cancer is significantly correlated with the mutational status of the *TP53* gene [11], which led us to test whether low *RAI2* gene expression might also be associated with genomic instability. Here we provide the first evidence that the RAI2 protein maintains genomic stability by contributing to efficient repair of DNA double strand breaks (DSB). In addition, RAI2 depletion appears to have additive effects with anticancer agents that induce DNA damage, which may further influence the response to chemotherapeutic treatment or metastatic relapse in human breast cancer.

## Methods

A comprehensive description of all materials and methods is provided as Supplementary Information. The description of live cell imaging, cytotoxicity profiling and immunoprecipitation combined with protein analysis by quantitative mass spectrometry proteomics, the rigor adherence table (Supplementary Table S1) and the key resources table (Supplementary Table S2) are provided as Supplementary Information only.

### Clinical in Silico validation

We used the Bioconductor package (RRID: SCR_006442) to download TCGA-BRCA transcriptome profiles, copy number segments, and clinical data. Normalized gene expression data for *RAI2* and other genes of interest in the METABRIC dataset were obtained from cBioPortal. Normalized *RAI2* gene expression was correlated with the expression of genes indicative of CIN (CIN70 score) using the weighted genome instability index (wGII). P-values were calculated by Student’s t-test. Multivariable regression analysis was performed to evaluate the association between CIN70 score and *RAI2* gene expression, adjusting for other covariates such as oestrogen receptor α (ER) status and PAM50 subtype and *TP53* status. For linear regression, RAI2 levels were log-transformed. For multivariable analysis, the Cox regression model was used, including those histopathological factors that were clinically significant in the univariable survival analysis in the multivariable analysis (tumour stage, ER status, HER2 status, grade, and molecular subtype) and presented as hazard ratio with 95% confidence interval. To assess whether *RAI2* gene expression correlated with patterns of genomic instability, copy number alteration (burden total number of somatic copy number variations (SCNAs) per tumour type) and point mutations (PM) burden were summed. Spearman correlations were performed to compare mRNA abundance and somatic variant burden on a per-gene basis. P-values were corrected using the Benjamini-Hochberg method to obtain false discovery rates (FDRs). For survival analysis, samples from the METABRIC dataset were divided by the median of *RAI2* gene expression and CIN70 score. Differences in five-year overall survival between these groups were determined by Kaplan-Meier analysis using the log-rank test. The Recombination Proficiency Score (RPS) was calculated for each tumour sample using normalized expression values for four signature genes involved in the DNA repair pathway (*RIF1*, *PARI*,* RAD51*, and *KU80*).

### Cell culture

MCF-7 (RRID: CVCL_0031), KPL-1 (RRID: CVCL_2094), MCF-10 A (RRID: CVCL_0598), CAMA-1 (RRID: CVCL_1115) and 293T (ATTC #CRL-3216) cells were cultured under standard conditions. Cell line authentication was performed using short tandem repeat profiling to exclude cross-contamination between cell lines. Cell lines were tested monthly for mycoplasma contamination.

### Plasmids, viral transduction and transfection

Plasmid construction for overexpression of wild-type RAI2 protein, as well as viral production and transduction procedures, have been described previously [11]. For knockdown experiments, we used plasmids derived from pLKO.1: non-target shRNA (Sigma-Aldrich #SHC016), shRNA1 (TRCN0000139927), and shRNA2 (TRCN0000441623). We used the pH2B-eYFP plasmid (plasmid #51002) to establish KPL-1 cells with constitutive H2B-eYFP overexpression.

### Gene expression profiling

500 ng total RNA from KPL-1 breast cancer cells was hybridized to the Illumina HT-12 Array v4 BeadChip (Illumina, San Diego, CA, USA) according to the manufacturer’s protocols. Microarrays were scanned using the Illumina iScan scanner (Illumina, San Diego, CA, USA) according to the standard Illumina scanning protocol. Bead-level data were aggregated using BeadStudio and normalized using the quantile method. Differential expression on normalized expression data was determined using the samr package (R version 3.4.2) with an unpaired t-test with a delta of 0.06 and a minimum twofold change in gene expression. Functional annotation of differentially expressed genes was performed using the gene functional classification tool in DAVID Bioinformatics Resources 6.8 (RRID: SCR_001881).

### Quantitative real-time RT-PCR analysis (qRT-PCR)

RNA was extracted from cultured cells during the exponential growth phase using the Nucleospin RNA Kit (Macherey Nagel, Germany) according to the standard protocol. 1000 ng of RNA from each sample was transcribed using the First Strand cDNA Synthesis Kit (Thermo Scientific, MA, USA) and random hexamers. Human Cell Cycle RT² Profiler™ PCR Arrays and reagents (Eppendorf, Germany) were used for cell cycle focused gene expression analysis. The qRT-PCR reactions were performed in triplicate using the Mastercycler Eppendorf Realplex thermal cycler (Eppendorf, Germany). Data analysis and significance testing were performed using QIAGEN GeneGlobe Data Analysis Centre (RRID: SCR_021211).

### Western blotting

Whole cell extracts from cultured cells were prepared by direct lysis and sonication of cells in 2% SDS sample buffer containing phosphatase and protease inhibitors. Cell extracts were separated on 8–15% denaturing polyacrylamide gels and blotted onto nitrocellulose or PVDF membranes. The following antibodies were used for detection: RAI2 (RRID: AB_2800292), Aurora A (RRID: AB_2665504), Aurora B (RRID: AB_10695307), Cyclin A2 (RRID: AB_627334), Cyclin B1 (RRID: AB_2783553), cyclin B2 (RRID: AB_2072392), survivin (RRID: AB_2063948), γH2AX (RRID: AB_2118009), and HSC-70 (RRID: AB_627761). HRP-conjugated anti-rabbit IgG, (RRID: AB_2099233) and (RRID: AB_330924) or infrared dye-labelled anti-rabbit IgG, (RRID: AB_621843) and anti-mouse IgG (RRID: AB_10956588) were used for detection. Differences between signal intensities in different cell lines and results of three independent experiments were evaluated by two-tailed Student’s t-test.

### Cell cycle analysis

Cell cycle profiles were assessed by quantifying DNA content using flow cytometry. Cells were fixed in 4% formaldehyde, treated with RNase and stained with propidium iodide. Flow cytometric analysis was performed using a NovoCyte Quanteon (Agilent, CA, USA) flow cytometer. A minimum of 20,000 cells were collected for analysis. After doublet discrimination, cell cycle profiles were automatically calculated using NovoExpress software (Agilent, CA, USA). The Watson model was used for cell cycle fitting. To determine the mitotic fraction, flow cytometry analysis of P-H3(S10) (RRID: AB_1549592) stained cells was performed using a FACS CantoII (Becton Dickinson, NJ, USA) equipped with FACSDiva software (RRID: SCR_001456). Three independent biological replicates were used to calculate the percentage of each cell cycle phase and the standard deviation. The difference was tested by two-tailed t-test.

### Immunofluorescence staining

Cells were fixed in 4% paraformaldehyde, washed three times with PBS, and permeabilized with 0.2% Triton X-100 in PBS. After incubation with 1% non-fat dry milk (w/v) in PBS for 30 min, cells were further incubated with primary antibodies: P-H3(S10) (RRID: AB_1549592), anti-centrosome (RRID: AB_212756), RAI2 (RRID: AB_2800292), CtBP1 (RRID: AB_399429), poly(ADP-ribose) (RRID: AB_785249), γH2AX (RRID: AB_2118009), or 53BP1 (RRID: AB_2921289). Specific antibody binding was visualized with Alexa Fluor 488 goat anti-rabbit IgG (RRID: AB_143165) and Alexa Fluor 546 goat anti-mouse IgG (RRID: AB_2534093). Confocal laser scanning microscopy was performed using a Leica TCS SP5 microscope (Leica, Germany) and Imaris imaging software (RRID: SCR_007370) to identify and measure the number of γH2AX and 53BP1 foci. At least 100 independent events were evaluated for quantification, and differences were tested by two-tailed t-test.

### Metaphase spread analysis

For metaphase spreads, exponentially growing KPL-1 cells were treated overnight with Colcemid (0.02 µg/ml), incubated with 0.0075 M KCl, fixed with methanol/acetic acid (3:1), dropped onto slides, stained with 5% Giemsa and mounted with Entellan before imaging with a Zeiss Axioplan 2 microscope (Zeiss, Germany). 100 metaphases per experiment were counted in three independent experiments.

### Traffic light reporter assay

The BFP-TLR-SceI plasmid (Addgene plasmid #31481) was digested with SceI (New England Biolabs, MA, USA) for 4 h, run on an agarose gel, and purified with a purification kit (Macherey Nagel, Germany) before being used for transfection. For RAI2 overexpression, HEK293T cells were first transfected with phCMV3-RAI2-HA [11] using OPTIMEM (Thermo Fisher, MA, USA) and Lipofectamin2000 (Invitrogen, MA, USA) according to standard protocol. After 24 h, the cells were cotransfected with 500 ng cut BFP-TLR-SceI and GFP donor plasmid (Addgene MA, USA, plasmid #31475). At 48 h post-transfection, trypsinised cells were quenched with media and mCherry, eGFP and BFP fluorescence signal was analysed by flow cytometry (LSR Fortessa, Becton Dickinson, NJ, USA) using 561 nm, 488 nm and 405 nm laser. The percentage of mCherry (for NHEJ events) and eGFP (for HR events) positive cells in the BFP positive cell fraction was used for analysis. Three independent biological replicates were used to calculate relative DNA repair efficiency. Differences were tested by two-tailed t-test.

## Results

### Low RAI2 expression is a feature of genetic unstable breast carcinomas

**Fig. 1:**
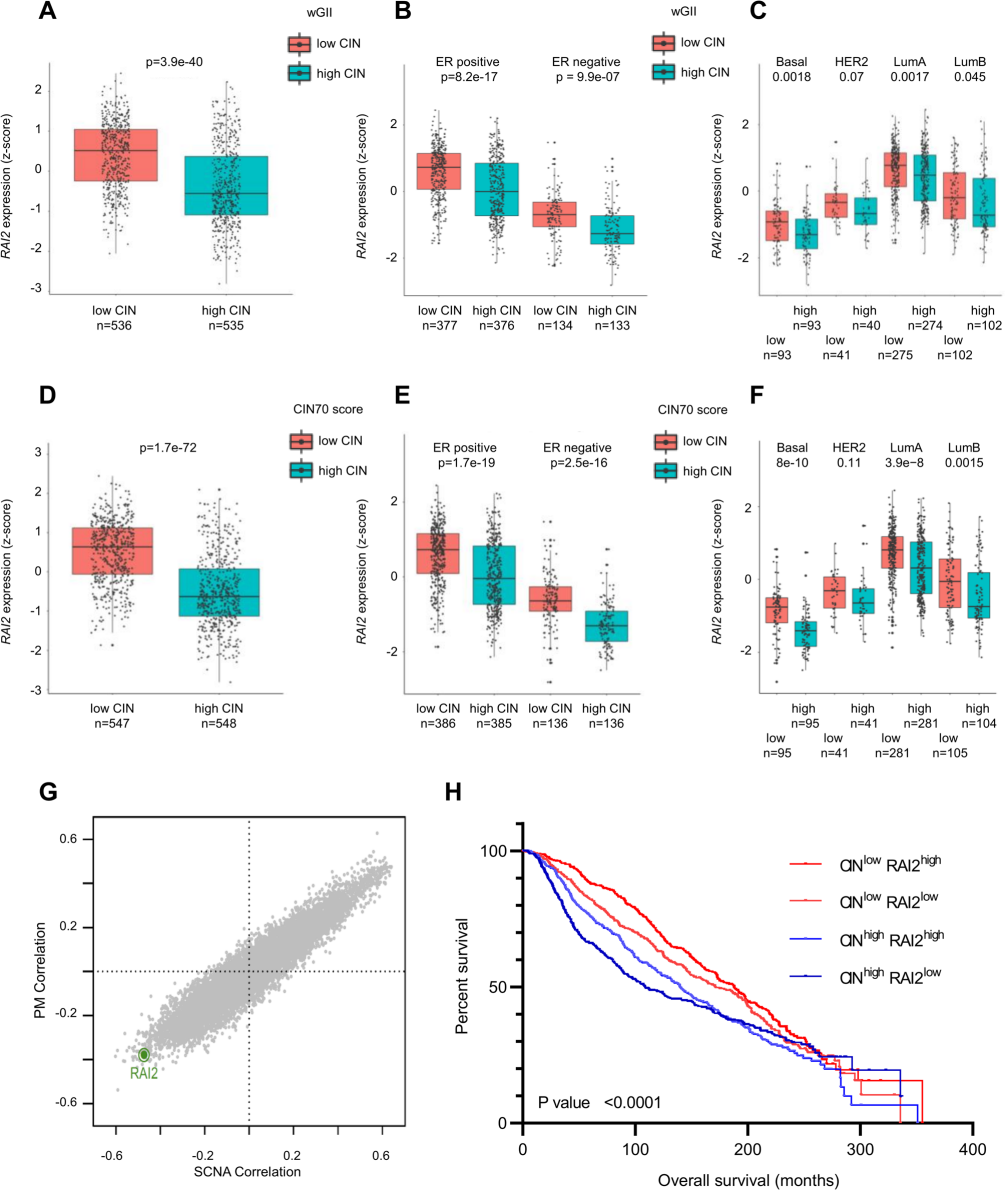
Low RAI2 gene expression is a feature of chromosomally unstable breast tumors and predicts poor patient outcome. **A-C)** Weighted genomic instability score (wGII). wGII score was calculated for the TCGA-BRCA cohort and patients were divided into two groups based on median wGII score. For each group, normalized gene expression of *RAI2* was plotted against wGII score in **(A)** all tumors, **(B)** in subgroups of ER and **(C)** in PAM50 molecular subtypes. Benjamini-Hochberg corrected p-values are presented. D-F) TCGA BRCA patients were divided into low and high CIN70 groups based on median CIN70 signature values. Normalized *RAI2* gene expression was plotted for each group. CIN70 stratification showing **(D)** all tumors and **(E)** in subgroups of ER and **(F)** in PAM50 molecular subtypes. Benjamini-Hochberg corrected p-values are presented. **(G)** The Cancer Genome Atlas (TCGA) data including exonic point mutations and somatic copy number alterations (SCNA) were examined to analyze the correlation between RAI2 gene expression and the pattern of genome instability. Copy number burden (total number of SCNAs per tumor type) and PM burden were summed. P-values were corrected using the Benjamini-Hochberg method to obtain FDRs. **(H)** Overall survival (OS) analysis of RAI2 gene expression stratified by median and CIN score in patients in the METABRIC dataset analyzed by Kaplan-Meier estimation. A two-tailed significance level below 0.05 was considered significant

To assess whether *RAI2* expression is associated with genomic instability in breast cancer patients, we performed an analysis of large published clinical datasets. First, we calculated the weighted genome integrity index (wGII) as an independent measure of CIN [13] and divided samples into low and high wGII groups based on the median cut-off. We found that *RAI2* gene expression was clinically lower in tumors with a high wGII score (Fig. 1A). We confirmed clinically differences in *RAI2* gene expression in estrogen receptor (ER)-positive and -negative subgroups (Fig. 1B) as well as in basal, luminal A and B molecular subtypes (Fig. 1C). We then examined *RAI2* gene expression in patient groups stratified according to the CIN70 signature of chromosomal instability derived from gene expression profiles [14, 15]. Consistent with the results from wGII, we observed clinically lower *RAI2* gene expression in samples with a high CIN70 score (Fig. 1D), ER-positive tumors showed higher *RAI2* gene expression compared to ER-negative tumors (Fig. 1E). However, the overall trend remained consistent in all groups of basal, luminal A and B molecular subtypes (Fig. 1F).

For validation a correlation of *RAI2* gene expression with the complete CIN70 score was assessed in the METABRIC breast cancer dataset [16]. Univariable analyses showed a clinically significant association between low *RAI2* gene expression and high CIN70 score (Figure S1A-C). Also, in multivariable linear regression analysis a significant association between the CIN70 score and low *RAI2* gene expression was found in the METABRIC data set (*p* < 0.001, multiplicative factor = 0.999 [CI:0.998–0.999]). Likewise, the basal subtype (*p* = 0.002, multiplicative factor = 0.949 [CI:0.918–0.982]) and HER2 (*p* = 0.038, multiplicative factor = 0.938 [CI:0.910–0.967]) showed an influence on *RAI2* gene expression compared to luminal A, whereas for ER and *TP53* no influence was seen (Figure S1D).

To evaluate these results, we correlated CIN70 gene expression with that of the *XPA*, *XPC*, *BRCA1* and *BRCA2* genes, whose gene products are known to sustain genomic integrity [17], as well as *B2M* and *KCNK3* as negative controls. As with *RAI2*, we found a strong anti-correlation between *XPA* and *XPC* gene expression and CIN70 genes. However, *BRCA1* and *BRCA2* gene expression is strongly correlated with CIN70 genes (Supplementary Figure S2). These findings also emerge when groups with low and high CIN70 or wGII scores are compared for individual genes (Supplementary Figure S3).

In the TCGA dataset we further investigated a possible correlation of *RAI2* gene expression with somatic copy number alterations (SCNA) and point mutations (PM) by a previously published approach [18]. *RAI2* was among the top genes (rank = 36 out of 12961) whose expression correlated inversely with aneuploidy (spearman rho = -0.47, FDR = 4.96 e-40) (Fig. 1G). Taken together these results confirm that compared to other genes in the genome there is a strong correlation between low *RAI2* gene expression and different characteristics of genome instability in primary breast tumors.

### Low RAI2 expression predicts poor clinical outcome in breast cancer patients

We then evaluated whether low *RAI2* gene expression identifies an aggressive subset of tumors associated with poor clinical outcome in the METABRIC dataset. Five-year overall survival analysis of patients showed that low *RAI2* gene expression combined with a high CIN score had a worse prognosis compared to other patient groups (*p* < 0.001). At five years, 36.2% of early relapse patients with low *RAI2* gene expression and high CIN score had died, while the best survival rate with only 9.4% of deaths was seen in patients with high *RAI2* gene expression and low CIN score. Patients with a mixed phenotype had intermediate 5-year survival rates of 79.4% and 82.1% (Fig. 1H). Multivariable analysis showed that the combined RAI2^low^/CIN^high^ phenotype was an independent poor prognostic factor (hazard ratio: 1.60 [CI: 1.05–2.42, *p* = 0.027]). Other independent risk parameters were grade (hazard ratio: 1.46 [CI: 1.08–1.99, *p* = 0.017]), ER status (hazard ratio: 1.88 [CI: 1.28–2.75, *p* = 0.001]) and stage (hazard ratio: 2.2 [1.63–2.98, *p* < 0.001]). In the subgroup analysis, we confirmed differences in overall survival for the RAI2^low^/CIN^high^ phenotype in patients with ER-positive and luminal B tumors. However, no significant results were found for patients with ER-negative, luminal A, HER2 and basal tumors (Figure S4). Taken together, these results indicate that low *RAI2* gene expression is a hallmark of genetically unstable tumors and that these patients have a significantly higher risk of early metastatic relapse.

### RAI2 depletion in human breast cancer cells causes deregulation of cell cycle-related genes and proteins

**Fig. 2:**
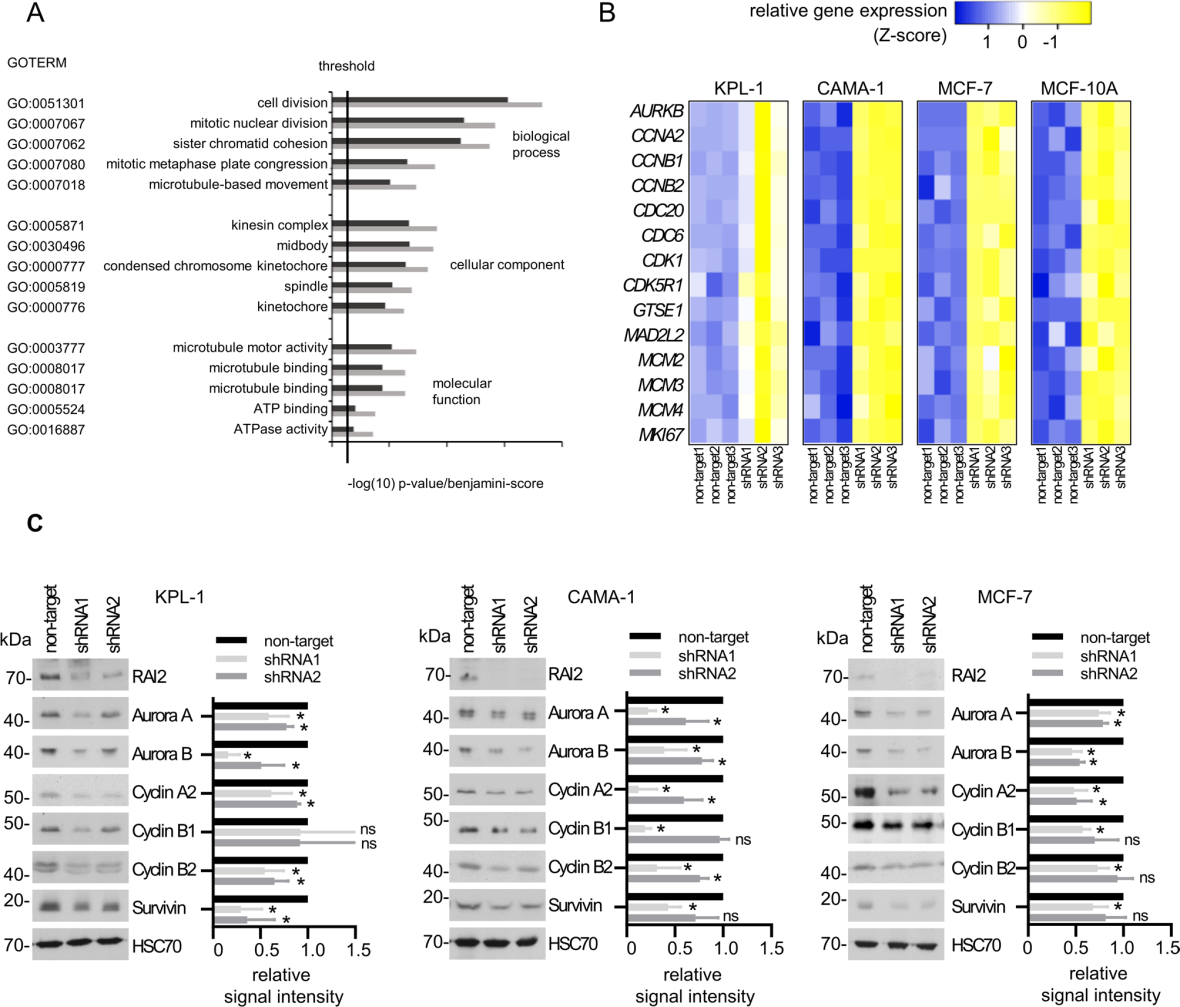
RAI2 depletion in human breast cancer cells causes deregulation of cell cycle-related genes and proteins. **(A)** Pathway analysis results of gene expression profiling showing the five most significantly enriched biological themes (GO terms) of molecular function, cellular component and biological process found in KPL-1 cells expressing RAI2-specific shRNA (dark gray). Plots show the negative decadic logarithm of Benjamini scores (black) and p-values (dark gray). The threshold indicates a negative decadic logarithm cut-off of 0.05. **(B)** Gene expression analysis of cell cycle related genes in RAI2-depleted cell lines using Human Cell Cycle RT² Profiler™ PCR Arrays. Fold change (FC) expression is shown relative to B2M expression and non-target shRNA-transduced control cells. **(C)** Western blot analysis and quantification of protein expression signals of RAI2 and previously identified cell cycle markers in whole cell extracts of the indicated cell lines after transduction with two independent RAI2-specific or non-target shRNAs. Detection of HSC70 protein is used as a loading control. * indicate a p-value below the significance threshold of 0.05 calculated by a two-sided t-test

To analyze whether loss of RAI2 protein function causes genomic instability, we first performed gene expression profiling by microarray analysis of the luminal breast cancer cell line KPL-1 after shRNA mediated RAI2 depletion, which has previously been shown to be a suitable cell line model to study RAI2 protein function [11]. Functional annotation revealed that the significantly deregulated genes are associated with the cell cycle, including deregulation of genes involved in microtubule motor activity, mitosis, and spindle apparatus (Fig. 2A and Supplementary Table S3 and S4). Therefore, the dysregulation of cell cycle-related genes was analyzed in more detail in additional RAI2-depleted cell lines (KPL-1, CAMA-1, MCF-7 and MCF-10 A) using pathway-specific arrays (Cell Cycle RT^2^ Profiler PCR Arrays). As shown in Fig. 2B and Supplementary Table S5, RAI2 depletion leads to decreased gene expression of *AURKB*,* CCNA2*,* CCNB1*,* CCNB2*,* CDC20*,* CDC6*,* CDK1*,* CDK5R1*,* GTSE1*,* MAD2L2*,* MCM2*,* MCM3*,* MCM4* and *MKI67* in all tested cell lines. The downregulation of selected gene products was validated by Western blot using two independent RAI2-specific shRNA sequences (Fig. 2C). We found reduced protein expression of Aurora A and B, Cyclin A2 in all three RAI2-depleted cell lines, and Cyclin B2 was significantly reduced only in KPL-1 and CAMA-1 cells. Since all these genes and proteins are known to be periodically regulated within the cell cycle [19], we tested whether the observed changes in gene expression and protein abundance correlated with changes in cell cycle distributions. In the cell lines tested, we found neither changes in the subpopulations in G1, S or G2 (Fig. 5A) nor consistent changes in the mitotic cell fractions of phospho-histone H3(S10)-positive cells (Figure S5B) or cell proliferation (Fig. 5C). Thus, we conclude that the observed deregulation of a key protein of the G2/M transition cannot be explained by changes in the overall cell cycle distribution, but rather by an effect on mitotic progression.

### RAI2 maintains mitotic accuracy

**Fig. 3:**
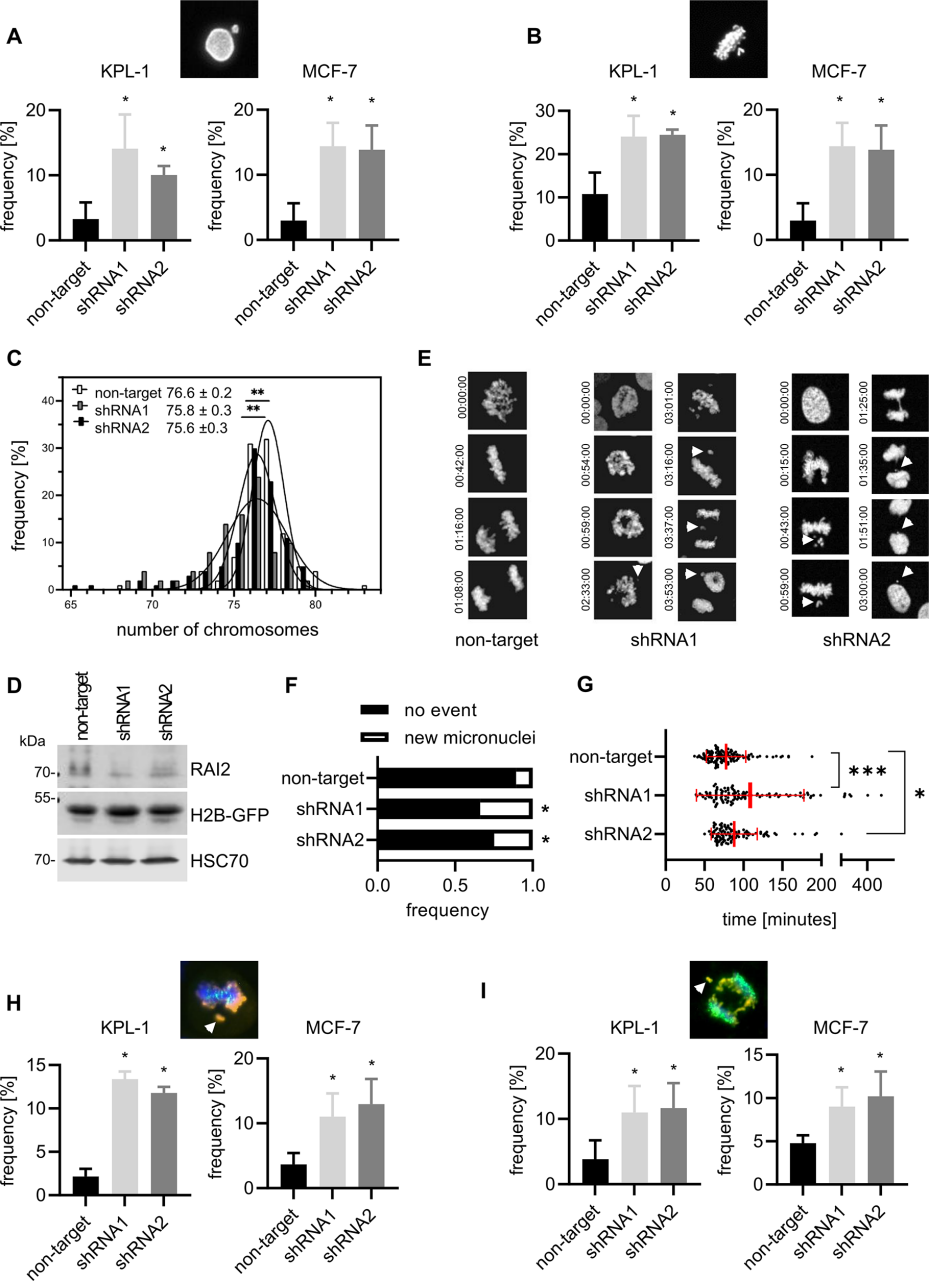
RAI2 depletion induces mis-segregation of acentric chromosomes and micronuclei formation in luminal breast cancer cells. **(A)** Analysis of the frequency of micronuclei during prophase and **(B)** frequency of incomplete metaphases in RAI2-depleted KPL-1 cells. The images show examples of scored events of immunofluorescence-stained cells with a phospho-histone H3 phosphorylation (S10) antibody. Frequencies are the mean of three independent experiments. * indicate a p-value below the significance threshold of 0.05 calculated by a two-sided t-test **(C)** Metaphase spreading of exponentially growing KPL-1 cells 10 days after transduction with non-target shRNA and RAI2-specific shRNA1. * indicate a p-value below the significance threshold of 0.05 calculated by a two-sided t-test. **(D)** Validation of RAI2 kockdown and over expression of H2B-GFP in whole cell extracts of the indicated cell lines after transduction with two independent RAI2-specific or non-target shRNAs. Detection of HSC70 protein is used as a loading control. **(E)** Examples of recorded cell divisions of control and RAI2-depleted KPL-1 cells with stable expression of the eYFP-Histone 2B fusion protein. Time in mitosis is indicated in hours: minutes: seconds and unaligned chromosome/chromosome fragments and micronuclei are indicated by arrows. **(F)** *De novo* micronuclei formation as assessed by live cell imaging of KPL-1 non-target and RAI2-depleted cells. **(G)** Total duration of individual cell divisions assessed from at least 100 cells of each cell line. *** indicate a p-value below the significance threshold of 0.001, * a p-value below 0.05 by a two-sided t-test. **(H)** acentric chromosome frequency during metaphase; and **(I)** anaphase. The images show examples of scored events of immunofluorescence-stained cells for DNA (blue), phospho-histone H3 phosphorylation (S10) (orange) and human centrosomes (green). Values shown are the mean frequency of three independent experiments. Student’s t-test was used for significant testing; * indicate a p-value below the significance threshold of 0.05 calculated by a two-sided t-test

To test whether RAI2 depletion influences mitotic progression, we analyzed individual phospho-histone H3(S10)-positive KPL-1 and MCF-7 cells and first assessed whether these cells exhibit macroscopic changes. In both cell lines, after RAI2 depletion, we found an increase in cells with micronuclei during prophase (Fig. 3A) as well as an increased frequency of metaphases with unaligned chromosomes (Fig. 3B), confirming that RAI2 depletion affects mitotic fidelity. Consistent with this, karyotyping of KPL-1 cells revealed a significantly reduced number of chromosomes in RAI2-depleted cells (Fig. 3C). To enable the analysis of individual cell divisions in real time, we established KPL-1 cells with stable expression of the eYFP-Histone 2B fusion protein and monitored these cells by live cell imaging after RAI2 depletion (Fig. 3D). By analyzing individual cell divisions (Fig. 3E), we observed increased *de novo* micronuclei formation in these cells (Fig. 3F) and found that the total mitotic time was longer in RAI2-depleted cells (Fig. 3G). To better characterize the observed chromosome segregation aberrations, we co-stained RAI2-depleted KPL-1 and MCF-7 cells with antibodies specific for phosphorylated histone H3 (S10) and centromeres. We found that the frequency of acentric fragments was increased during metaphase and anaphase in both cell lines (Fig. 3G-H) but did not observe relevant differences in the frequency of lagging chromosomes or an increase in chromosome bridges during metaphase and anaphase (Supplementary Fig. S6A-C). In conclusion, we found that RAI2 depletion impairs mitotic fidelity, probably by causing unrepaired DNA double strand breaks (DSB) that lead to chromosome segregation errors during mitosis.

### RAI2 depletion is synergistically cytotoxic with topoisomerase I and Aurora A inhibitors

**Fig. 4:**
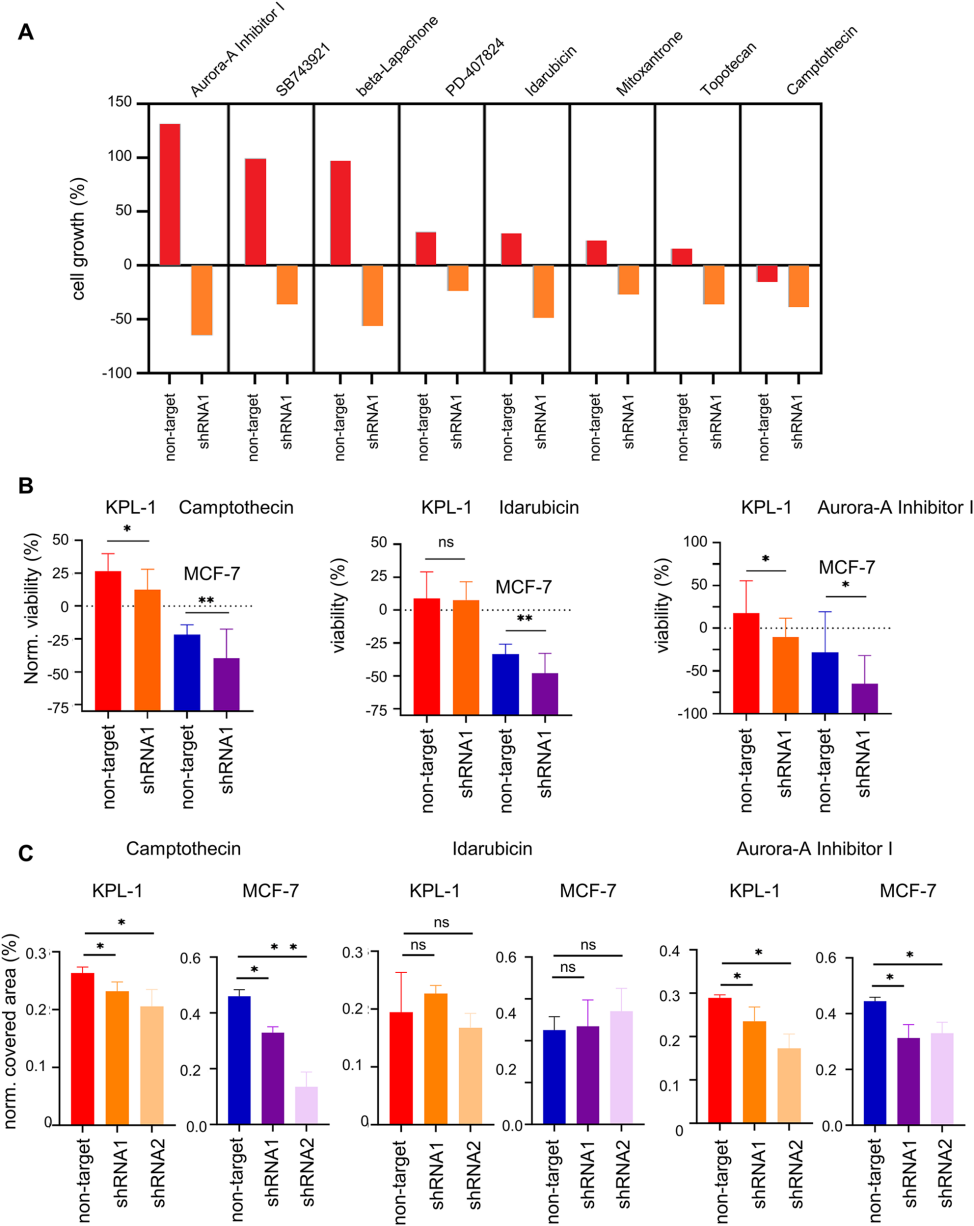
RAI2-depleted cells show increased sensitivity for topoisomerase I and Aurora-A inhibitors. **(A)** Analysis of cell viability of RAI2-depleted KPL-1 cells in the presence of indicated compounds from the 1280 Compound Containing LOPAC collection using the CellTiter-Glo assay using the CellTiter-Glo assay **(B)** Validation of increased sensitivity to treatment with Aurora A (300 nM), Camptothecin (30 nM) Idarubicin (100 nM) **(C)** Analysis of cell proliferation of RAI2-depleted KPL-1 and MCF-7 cells in the presence of indicated compounds. Data represent the mean of three independent experiments. For significant testing Student’s t-test was applied; * indicate a p-value below the significance threshold of 0.05 calculated by a two-sided t-test; n.s.: non-significant difference

To determine whether RAI2 depletion sensitizes cells to specific drug treatment, we performed a cytotoxicity screening using a library of 1280 pharmacologically active compounds. In the first screening phase, we tested and compared all components of the library to inhibit cell growth of control and RAI2-depleted KPL-1 cells. To select the best candidates for further validation, a threshold was set as < 80% cell growth of RAI2-depleted cells and > 80% cell growth of non-target shRNA-expressing cells after 48 h of incubation. Using these thresholds, we identified 27 compounds that inhibited cell growth in RAI2-depleted KPL-1 cells more efficiently than in control cells (Supplementary Table S6, Fig. 4A). For subsequent validation using a cell proliferation assay and a second cell line we selected camptothecin (topoisomerase I inhibitor), Idarubicin (topoisomerase II inhibitor) and Aurora-A inhibitor I. Significant differences were individually confirmed in three independent experiments for Aurora A at a concentration of 300 nM, for camptothecin at a concentration of 30 nM in both cell lines and for idarubicin at a concentration of 100 nM in MCF-7 cells only (Fig. 4B).

To confirm these findings, we performed confluence assays using the two independent shRNA sequences for RAI2 depletion and a longer incubation time of six days. We were able to confirm that cell confluence of KPL-1 RAI2-depleted cells is reduced by treatment with Aurora inhibitor I at a concentration of 300 nM and camptothecin at a concentration of 30 nM. For MCF-7 cells, we found differences for Aurora inhibitor I at a concentration of 100 nM and for Camptothecin at a concentration of 3 nM (Fig. 4C). For idarubicin, no differences between control and RAI2-depleted cells were found in this assay (Fig. 4C). In conclusion, we have shown that RAI2 depletion sensitizes KPL-1 and MCF-7 breast cancer cells to treatment with topoisomerase I and Aurora A inhibitors, indicating an additive effect with RAI2 inactivation. This suggests that chromosomal instability caused by RAI2 inactivation sensitizes cells to DNA-damaging agents.

### RAI2 protein is associated with the DNA damage response and poly-ADP ribosylation

**Fig. 5:**
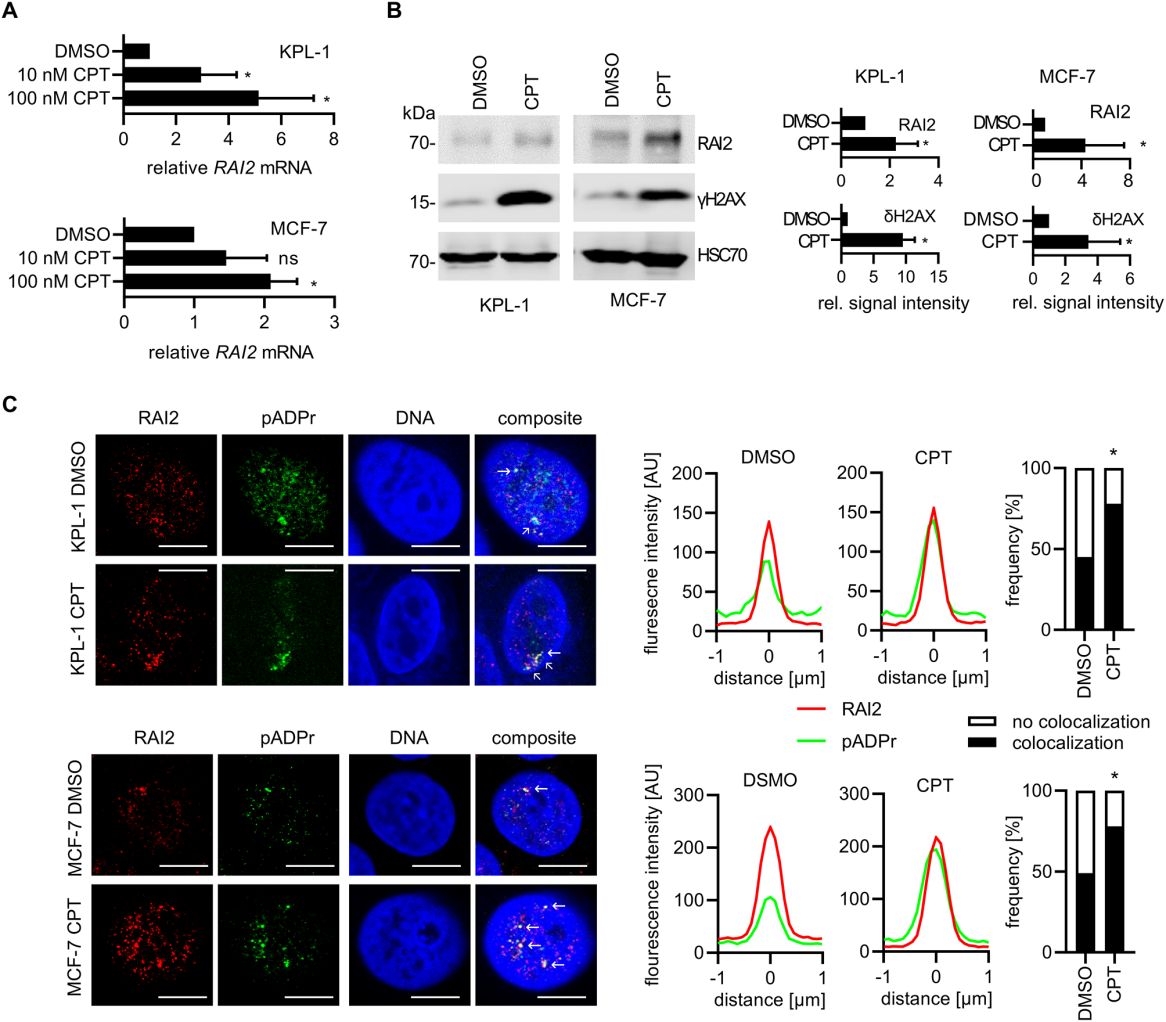
The RAI2 protein associates with DNA damage response and pADPr. **(A)** Quantitative RT-PCR analysis of DMSO- or camptothecin (CPT) treated KPL-1 and MCF-7 cells. RAI2 gene expression is expressed as the average fold change normalized to RPLP0 and DMSO-treated cells. Values are the average of three replicates. * indicate a p-value below the significance threshold of 0.05 calculated by a two-sided t-test, ns; not significant. **(B)** Western blot analysis and signal quantification of RAI2 and γH2AX in whole cell extracts of DMSO or CPT (100 nM) treated KPL-1 and MCF-7 cells. HSC70 was detected as a loading control. * indicate a p-value below the significance threshold of 0.05 calculated by a two-sided t-test. **(C)** Co-localization of RAI2 (red) with pADPr (green) and DNA (blue) in DMSO or CPT (100 nM) treated KPL-1 and MCF-7 cells. Quantification of fluorescence intensity profiles of overlay images by line scanning of 100 foci images of 2 μm distance each. Scale bars: 10 μm. White arrows indicate colocalization of RAI2 with pADPr

Based on these results, we analyzed RAI2 in cells treated with camptothecin. This genotoxic treatment resulted in an upregulation of *RAI2* gene expression (Fig. 5A) and protein levels, which correlated with an increase in DNA damage as indicated by increased γH2AX signal (Fig. 5B). To further investigate the molecular function of the uncharacterized RAI2 protein, we sought to identify interacting proteins. To this end, we overexpressed HA-tagged RAI2 in HEK293T cells and performed co-immune precipitation. Protein labeling was performed by stable isotope labeling of amino acids in cell culture (SILAC) followed by quantitative mass spectrometric bottom-up proteomics for protein identification and quantification. In this model we identified nine possible interaction partners of the RAI2 protein with high confidence (CtBP1, CLTC, HSPA8, NONO, HSPA9, HSPA1A, TNRC6B, UPF1 and AP2B1). In addition, we found eleven probable interactions with low confidence (HSPA5, PARP1, SFPQ, NUDT21, KRT8, DDX24, FXR1, HSPB1, COL17A1, SLC25A11, and FARSA) (Supplementary Table S7, Supplementary Figure S7). The highest SILAC ratio was found for CtBP1, which we previously described as a RAI2-interacting protein [11] and which has been shown to maintain mitotic fidelity [20, 21]. Also, the low-confidence interacting protein PARP1 represents a key molecule in response to various forms of DNA damage [22]. Interestingly, 75% of the other identified RAI2-interacting proteins, including PARP1 itself, are known substrates of PARP1 and are thus directly affected by post-translational poly-ADP-ribosylation (Supplementary Table S7) [23–29]. Therefore, we hypothesized that RAI2 may have a general affinity poly-(ADP-ribose) itself. To prove possible molecular interactions, we performed a colocalization analysis of the RAI2 protein with either CtBP1 or poly-(ADP-ribose) in untreated and camptothecin-treated KPL-1 and MCF-7 cells. As previously reported, RAI2 protein localizes to distinct nuclear foci [11]. In both untreated cell lines, the majority of these RAI2 foci colocalize with poly-(ADP-ribose) and the number of foci is further increased in the presence of camptothecin but does not affect the degree of colocalization in the tested cell lines (Fig. 5C; high resolution images are provided in Supplementary Figure S8). For CtBP1, we found colocalization only for some of the larger RAI2 foci in KPL-1 cells, whereas any colocalization was barely seen in MCF-7 cells but and was increased by genotoxic treatment (Figure S9). Taken together, we provide first evidence for a molecular function of the RAI2 protein within the DNA damage response associated with the presence of poly-(ADP-ribose). Since poly-ADP-ribosylation has multiple roles in different DNA repair pathways [22], we hypothesized that the previously observed phenotype of loss of mitotic fidelity might be the result of incomplete DNA repair prior to mitosis. To investigate a potential link to DNA repair, we performed another colocalization analysis of the RAI2 protein with γH2AX and 53BP1 foci, which are part of the early cellular response to the induction of DNA double-strand breaks [30], or actively promote DSB repair via non-homologous end joining (NHEJ) [31, 32]. We found that some of the γH2AX and 53PB1 foci did indeed colocalize with the RAI2 protein. In these cases, the RAI2 protein colocalizes in small areas of the larger γH2AX and 53PB1 foci; this effect is pronounced in cells treated with camptothecin (Supplementary Figure S10).

### Loss of RAI2 associated with failure of DNA repair

**Fig. 6:**
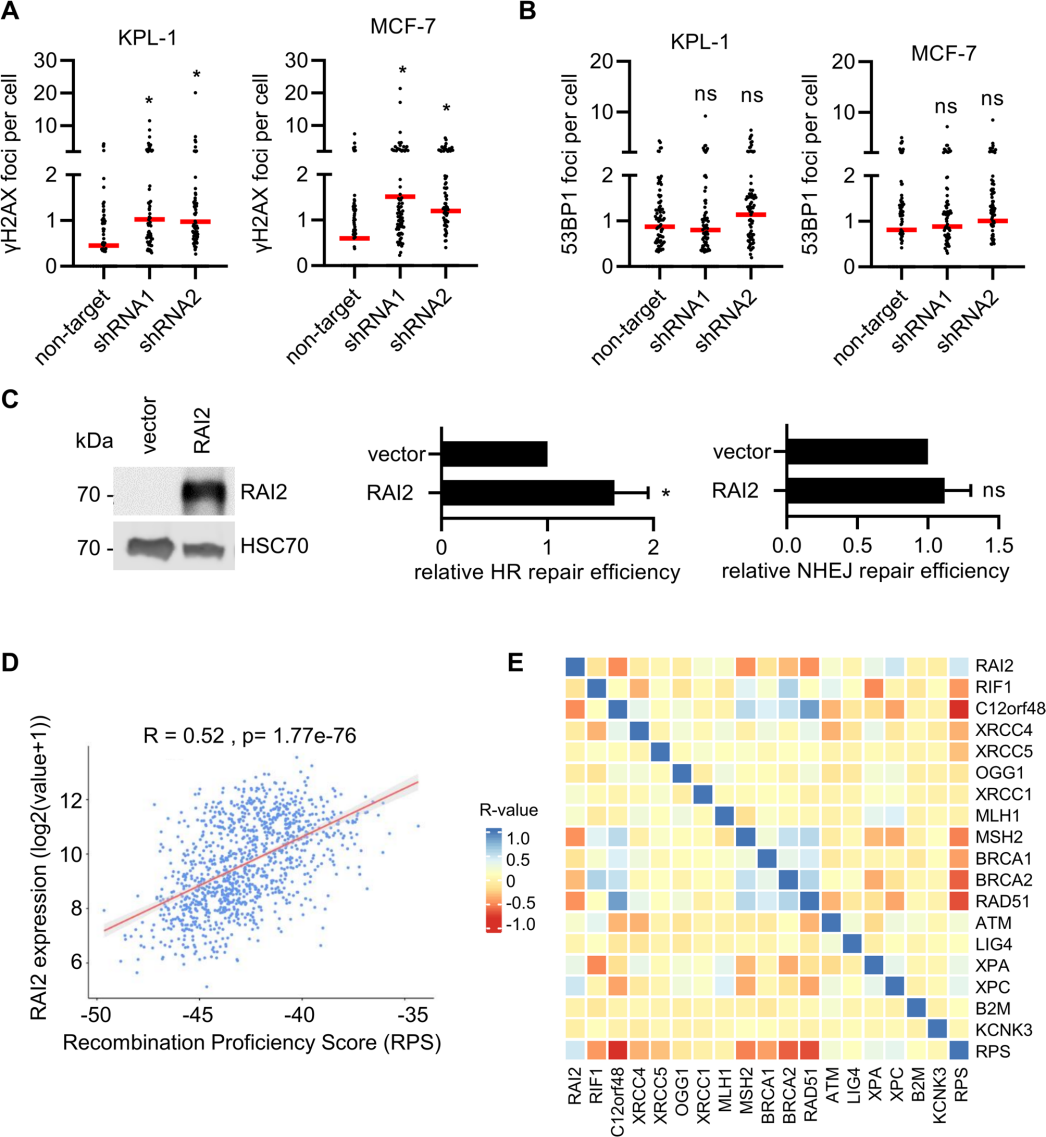
RAI2 contributes to efficient DNA repair. **(A)** Assessment of γH2AX foci in RAI2 depleted KPL-1 and MCF-7 cells. Cells were stained with anti γH2AX antibody and analyzed by confocal laser-scanning microscopy. The number of γH2AX foci in at least 100 individual cells were determined by image analysis software. * indicate a p-value below the significance threshold of 0.05 calculated by a two-sided t-test, ns; not significant. **(B)** Assessment of 53BP1 foci in RAI2 depleted KPL-1 and MCF-7 cells. Cells were stained with anti 53BP1 antibody and analyzed by confocal laser-scanning microscopy. The number of 53BP1 foci in at least 100 individual cells were determined by image analysis software.* indicate a p-value below the significance threshold of 0.05 calculated by a two-sided t-test, ns: not significant. **(C)** Traffic Light Reporter Assay analyzing relative efficiency of non-homologous end-joining (NHEJ) and homologous recombination (HR). Validation of RAI2-overexpressing HEK293T cells by Western blot analysis (left panel). Assessment of relative HR and NHEJ repair efficiency determined by flow cytometry. HR/NHEJ capacity was normalized to empty vector control. Values the mean of three biological replicates. * indicate a p-value below the significance threshold of 0.05 calculated by a two-sided t-test, ns; not significant. **(D)** Recombinant Proficiency Score- a gene expression-based score was used to quantify the DNA repair efficiency and correlation of RAI2 gene expression in TCGA-BRCA tumor samples. **(E)** Correlation of RAI2 gene expression with individual genes of HR deficiency signature in breast cancer. The heatmap displays the correlation values (r) computed using Pearson’s correlation. The heatmap legend on the left displays the r values, with a deeper red color indicating a negative and blue indicating a positive correlation. The gene expression of *B2M* and *KCNK3* serve as negative controls

Next, we investigated the effect of altered RAI2 expression on DNA DSB repair and the two major DSB DNA repair pathways, HR and NHEJ. First, we used confocal laser scanning microscopy to determine the number of γH2AX foci. As expected, we found that RAI2 depletion caused an increase in γH2AX foci in KPL-1 and MCF-7, indicating an overall increase in DSB lesions caused by RAI2 depletion (Fig. 6A). In contrast, we did not observe changes in 53BP1 foci in either cell line (Fig. 6B). To assess whether the increase in DSBs in RAI2-depleted cells could be caused by defects in DNA synthesis, we analyzed DNA replication by DNA fiber assay in RAI2-depleted KPL-1 and MCF-7 cells. We did not observe differences in DNA replication fork speed in RAI2-depleted cells compared to parental cells, either untreated or after hydroxyurea treatment (Supplementary Figure S7). To investigate potential effects of RAI2 on HR and NHEJ pathway choice, we used the plasmid-based traffic light reporter (TLR) assay [33] in HEK293T with transient RAI2 overexpression (Fig. 6C). We showed that forced RAI2 protein expression resulted in higher HR, whereas no relevant changes in NHEJ were observed in this setting (Fig. 6D). Taken together, these results indicate that inactivation of the RAI2 protein is likely to lead to an accumulation of unresolved DSBs. To validate a possible association of *RAI2* gene expression with altered DNA repair capacity in clinical samples, we calculated the recombination proficiency score (RPS), which predicts DNA DSB repair efficiency and sensitivity to DNA-damaging chemotherapy in breast cancer [34], for all TCGA samples and correlated the RPS score with *RAI2* gene expression. We found a positive correlation (Pearson correlation coefficient *r* = 0.52) between RAI2 expression and the RPS score and most of the individual genes that make up the RPS score (Fig. 6D-E). As with *RAI2*, the correlation with additional control genes representing different DNA repair pathways showed that *XPC*, *XPA* and *ATM* also have a positive correlation with the RPS score (Fig. 6E).

Thus, we could confirm that primary breast tumors with putative DNA DSB repair deficiency have low *RAI2* gene expression. To validate whether recomplementation of RAI2 expression could revert the phenotype of mitotic fidelity, sensitivity to camptothecin treatment and increased DNA damage, we repeated Western analysis, drug sensitivity testing and quantification of γH2AX foci in KPL-1 cells four weeks after initial RAI2 knockdown, which was previously shown to be transient effect [11]. We confirmed that RAI2 protein is fully restored four weeks after initial knockdown and that the effects of RAI2 depletion on these processes are reversed (Supplementary Figure S11).

## Discussion

When DNA repair mechanism are impaired or defective, several problems can arise that affect the accumulation of unrepaired DNA damage and premature mitotic entry [35]. We discovered three distinct related phenotypes in RAI2-depleted breast cancer cells that are indicative of such premature entry into mitosis [13, 36]: (i) *de novo* micronuclei formation, (ii) prolonged mitosis, and (iii) mis-segregation of acentric chromosomes. Since cells with persistent DNA damage and defective DNA repair are more susceptible to genomic instability as a hallmark of carcinogenesis [37], we conclude that defective DNA damage repair is the underlying mechanism for the observed phenotypes in RAI2-depleted breast cancer cells and demonstrate that low *RAI2* gene expression is a hallmark of genomically unstable breast tumors.

In addition to affecting primary tumor development, genomic instability is known to contribute to the critical progression from MRD to overt metastasis [38]. Because RAI2 was originally described as a potential metastasis suppressor that inhibits the hematogenous dissemination of estrogen receptor-positive breast cancer to the bone marrow [11], we conjecture that low *RAI2* gene expression in these studies was indicative of genomic instability contributing to the early onset of metastatic progression. Interestingly, although our previous finding showed that *RAI2* gene expression is strongly associated with the progression of ER-positive breast cancer [11], here we found that the functional association of loss of *RAI2* gene expression with genomic instability is seen in all molecular breast cancer subtypes. Thus, combined RAI2 and CIN assessment seems to characterize a new class of high-risk patients who may deserve a different form of therapy.

Despite the striking correlation of *RAI2* gene expression with clinical outcomes, the molecular function of the corresponding RAI2 protein is largely unknown. The in silico validation revealed that *RAI2* expression in primary breast tumors is correlated with expression of *XPC*, *XPA* and *ATM*. While the XPC protein serves as damage sensors and XPA as a mediator protein in nucleotide excision repair [17], the ATM kinase is responsible for the global orchestration of cellular responses to DSBs [39]. These findings may help to better inform to unravel the molecular function of the RAI2 protein. RAI2 is predicted to be an intrinsically disordered protein, lacking a defined three-dimensional structure [40], and likely prone to tolerate flexible binding partners and promote biomolecular condensate formation in a regulated manner [41, 42]. In this study, we provide the first evidence that the RAI2 protein, in association with poly-(ADP-ribose), is part of the cellular response to genotoxic stress and contributes to efficient HR. Since poly-(ADP-ribose) has multiple roles in DNA repair and chromatin remodeling [22, 43], including early recruitment of DNA repair proteins to DSB sites [44] and regulation of biomolecular condensate formation [45, 46], investigating whether and how the RAI2 protein is involved in the regulation of either of these mechanisms is an exciting prospect for the future.

Interestingly, we found that RAI2 depletion increased the number of γH2AX foci, indicating an increased basal level of DNA damage even in the absence of genotoxic treatment, but did not affect the number of 53BP1 foci. Based on the available data and the superficial knowledge of the underlying molecular mechanism, we can only speculate as to the reason for this observation. But it is likely that RAI2 inactivation leads to an accumulation of unresolved DSBs, as reflected by increased γH2AX foci. 53BP1 foci are known to be involved in repair pathway selection and function, particularly in the context of NHEJ [31, 32]. 53BP1 foci may remain constant because they are part of an early or initial response that is unaffected by RAI2 inactivation, regardless of repair failure. In other words, RAI2 inactivation may disrupt downstream repair processes after 53BP1 recruitment. The colocalization of RAI2 with pADPr suggests that it may be involved in related processes such as chromatin remodeling and modulation of DNA repair pathway choice and transition [43]. Since we found that forced RAI2 overexpression favors HR over NHEJ, we conclude that it may indeed play a potential role in DNA repair pathway choice or transition and that RAI2 inactivation may reduce the efficiency of DNA repair pathways, resulting in the accumulation of unresolved DSBs.

Although we were able to validate that RAI2 maintains genomic stability at various levels of evidence, there are potential limitations to this study. Although we have found evidence for a molecular interaction of the RAI2 protein with pADPr and an association with the DNA damage response, the exact molecular mechanism remains elusive. Also, little is known about the regulation of *RAI2* gene expression, and an open question is what controls the induction of *RAI2* expression after genotoxic exposure. Yan et al. provided initial evidence that hypermethylation of the *RAI2* gene is common in colon cancer [12], and potential binding sites of the AP-2 transcription factor family have been identified in the proximal promoter region of *RAI2* [47]. Since in this study we have found by multivariable linear regression analysis that low *RAI2* gene expression is not independent of *TP53* gene mutation status, we propose that regardless of the active role of RAI2 in response to genotoxic stress, low *RAI2* gene expression in tumor tissue may indicate a generalized DNA repair deficiency. However, the combination of correlative and causative effects may explain the strong value of low *RAI2* gene expression as a prognostic marker and as a biomarker of genomic instability.

## Conclusions

We provide first evidence for an active role of RAI2 in maintaining genomic stability and DNA repair. The exact underlying molecular mechanisms remains elusive but could be exploited to improve patient diagnosis and treatment. As the unbiased drug screening approach has revealed a synthetic sick interaction of RAI2 inactivation with topoisomerase I inhibitors, which are approved for the treatment of colorectal [48], ovarian [49] and small cell lung cancers [50], we envision that determining *RAI2* gene expression could potentially be used to improve chemotherapeutic outcomes in these cancers or may itself represent a target structure for a new pharmacological approach. Taken together, this study provides the first evidence for RAI2 protein in maintaining genomic stability and suggest a role in DNA repair paving the way for further functional and translational investigations.

## Electronic supplementary material

Below is the link to the electronic supplementary material.

Supplementary Material 1

Supplementary Material 2

Supplementary Material 3

Supplementary Material 4

Supplementary Material 5

Supplementary Material 6

Supplementary Material 7

Supplementary Material 8

Supplementary Material 9

Supplementary Material 10

Supplementary Material 11

Supplementary Material 12

Supplementary Material 13

Supplementary Material 14

Supplementary Material 15

Supplementary Material 16

Supplementary Material 17

Supplementary Material 18

Supplementary Material 19

## Data Availability

The microarray data are available via ArrayExpress with the primary accession code E-MTAB-7071. The mass spectrometry proteomics data are available via ProteomeXchange with the primary accession code PXD047189.

## Abbreviations

53BP1: Tumor suppressor p53-binding protein 1
ADP: Adenosine diphosphate
BFP: Blue fluorescent protein
BRCA: Breast cancer
CIN: Chromosomal instability
CtBP1: C-terminal-binding protein 1
DNA: Deoxyribonucleic acid
DSB: DNA double strand breaks
DTC: Disseminated tumor cell
ER: Oestrogen receptor alpha
eYFP: Enhanced yellow fluorescent protein
FACS: Fluorescence-activated cell sorting
FDR: False discovery rate
HER2: Human epidermal growth factor receptor 2
HR: Homologous recombination
MRD: Minimal residual disease
NHEJ: Non-homologous end joining
PAM50: Prediction analysis of microarray 50
PBS: Phosphate-buffered saline
PM: Point mutations
PVDF: Polyvinylidene fluoride
qRT-PCR: Quantitative reverse transcription polymerase chain reaction
RAI2: Retinoic acid-induced 2
RNA: Ribonucleic acid
RPS: Recombination proficiency score
SCNA: Somatic copy number variation
SDS: Sodium dodecyl sulfate
shRNA: Small hairpin RNA
SILAC: Stable isotope labeling of amino acids in cell culture
TCGA: The cancer genome atlas
TLR: Traffic light reporter
TP53: Tumor protein P53
wGII: Weighted genome instability index
γH2AX: Phosphorylated form of H2A histone family member X
