# Supplementary material for: Retinoic acid-induced 2 deficiency impairs genomic stability in breast cancer
Source: Breast Cancer Res. 2025 Jul 22;27:137. doi: 10.1186/s13058-025-02085-8 (PMC12285165; doi:10.1186/s13058-025-02085-8)
Supplement: Supplementary file 1 — Supplementary Material 1 [file 13058_2025_2085_MOESM1_ESM.pdf]

Retinoic Acid-Induced 2 Deficiency Impairs Genomic Stability in Breast Cancer

Supplementary Materials and Methods

Clinical *in silico* validation

We used the Bioconductor package TCGAbiolinks (RRID:SCR_006442, version 2.16.4) to download TCGA-BRCA transcriptome profiles, copy number segments and clinical data. Normalized gene expression data for *RAI2* and other genes of interest in the METABRIC dataset (1) were obtained from the cBioPortal (2, 3). Normalized *RAI2* gene expression was correlated with the expression of individual genes that have been previously been shown to be indicative of CIN by Carter and colleagues (4). Additionally, to assess chromosomal instability defined by somatic copy number alterations, we calculated the weighted genome instability index (wGII) as defined in (5). For each of the tumor sample, copy number segments were used to determine the weighted median integer copy number, with weights equal to the lengths of the copy number segments. The wGII of the tumor was calculated as the average fraction of aberrations across all 22 chromosomes, such that all chromosomes influence the score equally regardless of size.

The CIN70 score was calculated according to Birkbak et al. (6) by summing the expression values of all the CIN70 genes. P-values were calculated by Student's t-test. Multivariable regression analysis was performed to evaluate the association between CIN70 score and *RAI2* gene expression, adjusting for other covariates like ER status and PAM50 subtype and p53 status. For linear regression, *RAI2* values were log-transformed. For the multivariable analysis, the Cox regression model was used including those histopathologic factors that were clinically significant in the univariable survival analysis into the multivariable analysis (tumor stage, ER status, HER2 status, grade and molecular subtype) and presented as hazard ratio with 95% confidence interval. Due to the exploratory design of the study, adjustment for multiple testing was not performed and thus all p-values should be interpreted descriptively.

To assess whether RAI2 gene expression correlates with patterns of genomic instability characterized by the accumulation of somatic copy number alterations (SCNAs) and point mutations (PMs), TCGA exonic PM and SCNA data were examined. Briefly, copy number alteration burden (total number of SCNAs per tumor type) and PM burden were summed. Spearman correlations were performed to compare mRNA abundances and somatic variant burden on a per-gene basis as described previously. (7). P-values were corrected using the Benjamini-Hochberg method to obtain false discovery rates (FDRs).

For survival analysis, samples from the METABRIC dataset were divided by the median of RAI2 gene expression and CIN score. Differences in five-year overall survival between these groups were determined by Kaplan-Meier analysis using the log-rank test.

Recombination Proficiency Score (RPS) was calculated for each tumor sample using normalized expression values for four signature genes involved in the DNA repair pathway (RIF1, PARI, RAD51, and KU80). High expression of these genes indicates low RPS. (8). The score was calculated as follows:

$$RPS = -1 \times (RIF1 + PARPBP + RAD51 + XRCC5)$$

Cell culture

Cell line authentication was performed using short tandem repeat profiling to exclude cross-contamination between cell lines. Cells were grown as monolayers under standard conditions in either DMEM or RPMI supplemented with 10% FBS and 2 mM L-glutamine at 37°C in a humidified atmosphere containing 10% CO2 or 5% CO2, respectively. MCF-10A cells were cultured in (1:1) DMEM:F12 medium supplemented with 5% horse serum, 100 ng/mL cholera toxin, 20 ng/mL EGF, 500 ng/mL hydrocortisone, and 10 µg/mL insulin. To establish KPL-1 cells with constitutive expression of the eYFP-H2B fusion protein, the pH2B-eYFP plasmid (gift of Rusty Lansford, Addgene, MA, USA, plasmid #51002) was transfected using Lipofectamine 2000 (Invitrogen, MA, USA) according to the manufacturer's protocol. After 72 hours, eYFP-positive cells were enriched by fluorescence-activated cell sorting. This procedure was repeated after 10 days to enrich cells with stable expression of the eYFP-H2B fusion protein.

Plasmid construction and viral transduction

Plasmid construction for overexpression of wild-type RAI2 protein, as well as viral production and transduction procedures, have been described previously (9). For depletion of RAI2 expression lentiviral pLKO.1 shRNA vectors targeted against human RAI2 (shRNA1 TRCN0000139927 (GAGCTCAATCCCGAATGGCAAT) and shRNA2 TRCN0000441623 (TTGCCTGTGCCAGTCCCTATT)) were obtained from the RNAi Consortium (http://www.broadinstitute.org/rnai/trc/lib).

Gene expression profiling

500 ng total RNA from KPL-1 breast cancer cells was hybridized to the Illumina HT-12 Array v4 BeadChip (Illumina, San Diego, CA, USA) according to the manufacturer's protocols. The Illumina TotalPrep RNA Amplification Kit (Thermo Fisher Scientific, MA, USA) was used to generate single-stranded cRNA from input amounts of 500 ng total RNA (label: biotin, Cy3-streptavidin). 750 ng of cRNA was hybridized on the Illumina HumanHT-12 v4 BeadChip for 17 hours at 58°C. Gene expression microarrays were scanned using the Illumina iScan scanner according to the Illumina standard scanning protocol. Bead-level data was aggregated using BeadStudio and normalized using the quantile method. Differential expression on normalized expression data was determined using the samr package (R version 3.4.2) with an unpaired t-test with a delta of 0.06 and a minimum twofold change in gene expression. Functional annotation of differentially expressed genes was performed using the gene functional classification tool in DAVID Bioinformatics Resources 6.8 (RRID:SCR_001881).

Quantitative real-time RT-PCR Analysis (qRT-PCR)

RNA was extracted from cultured cells during the exponential growth phase using the Nucleospin RNA Kit (Macherey Nagel, Germany) according to the standard protocol. 1000 ng of RNA from each sample was transcribed using the First Strand cDNA Synthesis Kit (Thermo Scientific, MA, USA) and random hexamers. Human Cell Cycle RT^2 Profiler™ PCR Arrays and reagents (Eppendorf, Germany) were used for cell cycle-focused gene expression analyses. The qRT-PCR reactions were performed in triplicate using the Mastercycler Eppendorf

Realplex thermal cycler (Eppendorf, Germany). Data analysis and significance testing were performed using QIAGEN's web-based software for cataloged arrays (RRID:SCR_021211).

Western blotting

Whole cell extracts from cultured cells were prepared by direct lysis and sonication of cells in 2% SDS sample buffer containing phosphatase and protease inhibitors. Cell extracts were separated on 8% to 15% denaturing polyacrylamide gels and blotted onto nitrocellulose or PVDF membranes. Protein detection was performed by incubation with the following specific antibodies RAI2 (RRID:AB_2800292), Aurora A (RRID:AB_2665504), Aurora B (RRID:AB_10695307), Cyclin A2 (RRID:AB_627334), Cyclin B1 (RRID:AB_2783553), Cyclin B2 (RRID:AB_2072392) Survivin (RRID:AB_2063948) and γH2AX (RRID:AB_2118009). HSC-70 (RRID:AB_627761) served as loading control. For detection we used HRP conjugated anti-rabbit IgG, (RRID:AB_2099233) and (RRID:AB_330924) or infrared-dye labeled anti-rabbit IgG, (RRID:AB_621843) and anti-mouse IgG (RRID:AB_10956588). For protein detection we used either the Curix 60 processor (Agfa HealthCare, Belgium) on Super RX films (Fujifilm, Japan) or the Odyssey® CLx Imaging System (Li-Cor Biosciences, NE, USA). Differences between signal intensities in different cell lines and results of three independent experiments were evaluated by two-tailed Student's t-test.

Cell cycle analysis

The cell cycle profiles were assessed by quantifying DNA content using flow cytometry. Cells were grown to 70-80% confluence, detached from the flask by trypsinization and collected by centrifugation at 1200 xg for 2 minutes. Cells were resuspended in 500 µL 1x PBS, centrifuged at 800 xg for 3 minutes, and the supernatant carefully removed without shedding cells. Cells were fixed in 4% formaldehyde for 10 minutes at 37°C for 1 minute and placed on ice. The cells were centrifuged at 800 xg for 3 minutes, the supernatant was discarded, and the cells were resuspended in ice-cold 90% methanol and incubated on ice for 30 minutes. Methanol was removed by centrifugation at 800 xg for 3 minutes, and the cell pellet was resuspended in 1 mL 0.5% BSA/PBS. Cells were washed twice with 0.5% BSA/PBS by centrifugation at 800

xg for 3 minutes. After the last wash, the cells were resuspended in 500 µL 0.5% BSA/PBS, and 10 µL RNase A and 5 µL propidium iodide solution were added for at least 30 minutes at RT. Samples were stored at 4°C until flow cytometric analysis using a NovoCyte Quanteon (Agilent, CA, USA) flow cytometer. A minimum of 20,000 cells were collected for analysis. After doublet discrimination, cell cycle profiles were automatically calculated using NovoExpress software (Agilent, CA, USA). The Watson model was used for cell cycle fitting. Three independent biological replicates were used to calculate the percentage of each cell cycle phase and the standard deviation. Difference was tested by two-tailed t-test.

To determine the mitotic fraction, flow cytometry was performed using a FACS CantoII (Becton Dickinson, NJ, USA) equipped with FACSDiva software (RRID:SCR_001456). For combined analysis of DNA content and H3 phosphorylation, cells were fixed in 4% formaldehyde for 10 minutes at 37°C. Cells were then cooled on ice, spun down at 850xg, resuspended in ice-cold 90% methanol, and incubated on ice for 30 minutes. For immunostaining, the methanol was removed by centrifugation and the cells were incubated in 0.5% BSA (w/v) in PBS containing P-H3(S10) primary antibody (1:500; CST, RRID:AB_1549592) for 1 hour at room temperature, followed by incubation with Alexa-Fluor-488-conjugated secondary antibody (RRID:AB_143165) for 30 minutes at room temperature. DNA was stained with propidium iodide buffer for 30 minutes at room temperature. Flow cytometry was performed using a FACS CantoII (Becton Dickinson NJ, USA) equipped with FACSDiva software (RRID:SCR_001456).

Immunofluorescence staining

Cells were fixed in 4% paraformaldehyde in PBS for 10 minutes, washed three times with PBS, and permeabilized with 0.2% Triton X-100 in PBS for 10 minutes. After incubation with 1% nonfat dry milk (w/v) in PBS for 30 minutes, the cells were further incubated with primary antibodies P-H3(S10) (1:500; RRID:AB_1549592) and human anti-centrosome (1:100; RRID:AB_212756) diluted in 1% nonfat dry milk (w/v) in PBS for 1 hour. For co-staining of RAI2 (1:250, RRID:AB_2800292), CtBP1 (1:250, RRID:AB_399429), pADPr (1:250, RRID:AB_785249), and 53BP1 (1:250, RRID:AB_2921289) antibodies were diluted in 1% BSA

(w/v) in PBS. After three washes with PBS, specific antibody binding was visualized with Alexa Fluor 488 goat anti-rabbit IgG (RRID:AB_143165) and Alexa Fluor 546 goat anti-mouse IgG (RRID:AB_2534093) in 1% nonfat dry milk (w/v) in PBS. For γH2AX staining, fixation and permeabilization were performed as described above. Cells were blocked with 3% BSA/PBS for 1 hour and incubated with γH2AX antibody (1:800, RRID:AB_2118009) followed by incubation with Alexa Fluor 488 goat anti-rabbit IgG (H+L) (Life Technologies. CA, USA). After three washes with PBS, nuclei were stained with DAPI and mounted in Mowiol (Sigma-Aldrich, MO, USA) according to the manufacturer's instructions. Confocal laser scanning microscopy was performed using a Leica TCS SP5 microscope (Leica, Germany) and imaging software. Imaris imaging software (RRID:SCR_007370) was used to identify and measure the number of γH2AX and 53BP1 foci. For this purpose, we applied uniform background subtraction and a lower volume cut-off for foci of 0.5 μm^3 in statistical analysis by two-tailed Student's t-test. Colocalization of RAI2 foci with pADPr or CtBP1 was verified using fluorescence intensity profiles measured along lines manually drawn on the microscopy images using ImageJ software (RRID:SCR_003070) (10). 100 individual RAI2 foci of each condition were identified macroscopically in single image stacks. Lines were selected to intersect foci observed on channels corresponding to pADPr or CtBP1, ignoring any other signal. This procedure resulted in 100 intensity profiles with two channels for each of the four conditions. The profiles were aligned by applying a manual horizontal shift maximizing in the RAI2 channel. Shifts were applied to a second channel of each profile prior to calculation of mean intensities and plotting.

Metaphase spread analysis

For metaphase spreads, exponentially growing KPL-1 cells were treated with Colcemid (0.02 µg/ml) overnight, incubated with 0.0075 M KCl, fixed with methanol/acetic acid (3:1), dropped onto slides, stained with 5% Giemsa and mounted with Entellan before imaging with a Zeiss Axioplan 2 microscope. 100 metaphases per experiment were counted in three independent experiments.

Live cell imaging

KPL-1 cells stably expressing eYFP-H2B were transduced with RAI2-specific and non-target control shRNAs and after 7 days the cells were transferred to chamber culture slides (Ibidi, Germany #80426). To maintain cell viability, cells were kept in a humidified environmental chamber supplied with 5% CO2 at 37°C. Cell divisions were imaged using a Visitron SpinningDisk microscope (Olympus, Germany) and low-power 488 nm laser excitation with a 40x/NA1.3 Plan Fluor oil objective and a high-sensitivity EM-CCD camera for detection. 20 µm z-stacks with 5 µm spacing were acquired as a time-lapse series at 1 min intervals. This acquisition setup allowed for high-resolution data to be obtained and critically reduced phototoxic effects that would otherwise interfere with cell cycle progression. As an additional control, cells were cultured in an external incubator for 24 hours and images were acquired at 0 hour and 24 hours to assess the frequency of micronuclei formation and thus the impact of continuously absorbed radiation on mitotic fidelity. Images were processed in FIJI (11) by applying maximum intensity projection, background correction, and noise reduction. In two independent experiments, mitotic cells were analyzed by scanning 5 to 10 positions in the chamber slide over 24 hours. At least 100 cell divisions per cell line were recorded and used for evaluation. The total duration (in minutes) of mitosis and of individual mitotic stages, as well as de novo micronuclei formation, were measured.

Cytotoxicity profiling

The assay is described in all detail in Ellinger et al. (12). Briefly, 1500 cells per well of KPL-1 cells expressing either RAI2-specific or non-targeted shRNA were seeded at 20 µL per well in white, 384-well, PS, Cellstar plates (Greiner Bio-One, Germany) and incubated at 37°C in the presence of 5% carbon dioxide. At 24 hours post seeding, baseline growth was assessed using a control plate and CellTiter-Glo (CTG) reagent (Promega, WI, USA). Briefly, 20 µL of CTG detection mix was added to each well and plates were analyzed on an EnVision Multimode reader (PerkinElmer, MA, USA) after 10 min incubation in the dark. In parallel, four assay plates were dosed with compounds from the 1280 Compound Containing LOPAC collection

(Sigma Aldrich, MO, USA) at a final concentration of 10 µM in singlicates. Plates were analyzed using CellTiter-Glo as described after incubation for 48 hours at 37°C in the presence of 5% carbon dioxide. Raw data were normalized to percent cell growth using the baseline growth and the corresponding high control (C) containing only the solvent DMSO (Carl Roth, Germany). The measured luminescence signal of a specific sample (S) was converted into percent cell growth compared to the average signal of the baseline control (B). In case of a sample signal higher than the average baseline, the following formula was used: percent effect=(S-B)/(C-B)x100. If the sample signal was lower than the average baseline, the following formula was used: percent effect=(S-B)/Bx100. This relative growth was used to identify 112 compounds that were analyzed in triplicate using the above assay. The eight most promising compounds were analyzed in four KPL-1 and MCF-7 cell lines with the previously described genetic backgrounds using 4000 cells per well in 11 pt dose response.

Immunoprecipitation and protein analysis by quantitative mass spectrometric proteomics

The SILAC protein quantification kit (Pierce Biotechnology; MA, USA) was used for metabolic labeling. HEK293T cells were grown in DMEM medium supplemented with 10% dialyzed FBS, 2 mM L-glutamine and proline 55 mg/l. For heavy isotope labeling, 13C6-Arginine-HCl (50 mg/l) and 13C6-Lysine-2HCl (25 mg/l) were added to the medium after sterile filtration resulting in complete SILAC medium. For preparative labeling, cells were cultured in 75 cm2 cell culture flasks containing SILAC medium under standard conditions for 14 days. Cells were transfected with 4 µg of phCMV3 expression plasmids containing either wild-type or mutant RAI2 cDNA sequence using Lipofectamine 2000 (Life Technologies, CA, USA). After 48 hours, the cells were lysed in buffer containing 50 mM Tris/HCl, pH 8, 0.4% NP-40, 300 mM NaCl and 10 mM MgCl2 plus phosphatase and protease inhibitors. Cell nuclei were pelleted by centrifugation and equal volumes of dilution buffer containing 50 mM Tris/HCl, pH 8 and 0.4% NP-40 were added to each sample. Protein complexes were precipitated with 1 mg of total protein from each sample and 40 µL of anti-HA agarose slurry (Abcam, ab214758) for 16 hours at 4°C. Washing was performed in Pierce® spin columns (Pierce Biotechnology; MA, USA) with buffer

containing 50 mM Tris/HCl, pH 8, 0.4% NP-40, 150 mM NaCl, and 5 mM MgCl2. Finally, protein samples were boiled in 2x SDS sample buffer. Protein concentration was determined using the Pierce BCA Protein Assay Kit (Pierce Biotechnology, MA, USA) according to the manufacturer's instructions using BSA as a standard. A 1:1 mixture of preparations from control and RAI2-transfected cells was then separated by SDS-polyacrylamide gel electrophoresis (PAGE). Protein separation was performed on a Novex XCell Sure-Lock mini system (Invitrogen, MA, USA) using 10% polyacrylamide separating gels and a Laemmli buffer system. Samples were diluted in SDS sample buffer, heat denatured at 95°C for 5 minutes, and loaded onto the gels. The molecular size standard was the peqGOLD protein marker V (Peqlab, Germany). Protein visualization was performed by colloidal Coomassie staining of SDS gels according to Neuhoff. (13). Coomassie-stained lanes from three independent experiments were cut into ten pieces each. In-gel reduction, alkylation with iodoacetamide, tryptic digestion, and extraction of peptides were performed as described (14).

Peptides were dried in vacuo and resuspended in buffer A (0.1% FA) before injection into a nano-ultra-pressure liquid chromatography system (Ultimate 3000 RSLCnano, Thermo Fisher, MA, USA) coupled to a tandem mass spectrometer (Orbitrap Fusion, Thermo Fisher, MA, USA) with a nano-electrospray ionization (nano-ESI) source. Peptides were trapped on a reversed-phase trap column (2 cm x 75 µm ID; Acclaim PepMap trap column packed with 3 µm beads, Thermo Fisher, MA, USA) and separated on a reversed-phase column (25 cm x 75 µm ID, Acclaim PepMap, 3 µm beads, Thermo Fisher, MA, USA). The column temperature was maintained at 45ºC. Each sample was separated using a two-step 70-minute gradient starting with 3% buffer B (99.9% ACN, 0.1% FA) to 28% in 35 minutes, increasing to 35% in 5 minutes, followed by a ramp to 90% for 10 minutes and equilibration to 3% for 20 minutes at a flow rate of 300 nL/min. Eluting peptides were transferred to the mass spectrometer via the nano-ESI source. Data were acquired in data dependent acquisition mode using the top speed setting for precursor selection for fragmentation using higher energy collisional dissociation. Full scan spectra were acquired in the Orbitrap at a resolution of 120000 with a scan range of 300-1500 m/z. The automatic gain control target was set to 2 x 105 with a maximum injection time of 120

ms. A dynamic exclusion list of 30 seconds was used. Fragment ion spectra were acquired in the ion trap with the scan rate set to fast. The AGC target was set to 1*104 with a maximum injection time of 35 ms. Data analysis was performed using MaxQuant software (version 1.5.8.3) (15). Data were loaded into the software and labeled as fractions corresponding to the experiment (ten fractions for each replicate). Spectra were searched against the human FASTA database, which was obtained from Uniprot in October 2014 and contains 20193 entries. An additional contamination database was used (provided by the software). Cysteine carbamidomethylation was set as fixed modification, oxidation on methionine and N-terminal acetylation were set as variable modifications. Arg6 and Lys6 were set as heavy labeled amino acids. Trypsin was selected as the enzyme; two missed cleavages were allowed. The minimum peptide length was set to six amino acids. Identified peptides were filtered to a false discovery rate of 1%. Default parameters were used for all other settings. For protein identification, only those peptide sequences determined from the mass spectrometry data that corresponded to a single individual protein (unique peptides) were considered. To identify interacting proteins with high confidence, SILAC sample/mock ratios were first converted to log2 value ratios, and the frequency distribution was calculated. The data were then used to calculate a 95% confidence limit (24). Proteins that were found to be significantly up-regulated in at least two of the three independent replicates were considered to be interacting proteins with high confidence, whereas proteins that were found to be up-regulated in only one replicate were considered to be interacting partners with low confidence.

Fiber assay

Exponentially growing cells were pulse-labeled with 25 µM CldU (Sigma-Aldrich, MO, USA) and 250 µM IdU (Sigma-Aldrich, MO, USA) for 20 minutes and treated with hydroxyurea (HU) for 4 hours in one experiment. After labeling, cells were harvested, and fiber spreads were prepared from 5 × 105 cells/ml. Cells were fixed on slides in MeOH/AcOH and incubated in 2.5 M HCl for 90 minutes. Slides were incubated for 1 hour in blocking buffer (2% BSA [w/v] in PBS with 0.1% Tween) and then stained for 1 hour at 37°C with rat monoclonal anti-BrdU antibody (abD Serotec, clone BU1/75, 1:1000 in blocking buffer) to detect CldU and mouse

monoclonal anti-BrdU antibody (BD Bioscience, clone B44, 1:1000 in blocking buffer) to detect IdU. Goat anti-rat AlexaFluor 555 and goat anti-mouse AlexaFluor 488 (both Invitrogen, 1:500 in blocking buffer) were used as secondary antibodies and incubated for 2 hours at RT. Slides were mounted in Immuno-Fluor mounting medium (MP Biomedicals; CA, USA). Fiber tracts were examined by fluorescence microscopy, images were taken from randomly selected fields with untangled fibers and analyzed using ImageJ software. Replication fork velocities of CldU and IdU were measured, and micrometer values were converted to kilobases. A conversion factor for the length of a labeled lane of 1 μm=2.59 kb was used. A minimum of 100 individual fibers were analyzed for each experiment.

Traffic light reporter assay

The BFP-TLR-SceI plasmid (Addgene MA, USA, plasmid #31481) (16) was digested with SceI (NEB) for 4 hours, run in an agarose gel and cleaned up (NucleoSpin Gel and PCR Clean-up, Macherey Nagel, Germany) before being used for transfection. HEK293T cells were seeded and shRNA mediated RAI2 knockdown was performed as describes above. 24 h after induction of the RAI2 knock-down, cells were transfected with 500 ng cut BFP-TLR-SceI and GFP donor plasmid (Addgene plasmid #31475) (16) using OPTIMEM (Thermo Fisher, MA, USA) and Lipofectamin2000 (Invitrogen, MA, USA). 48 hours post-transfection trypsinized cells were quenched with media and mCherry, eGFP and BFP fluorescence signal was analyzed by flow cytometry (LSR Fortessa, Becton Dickinson, NJ, USA) using 561 nm, 488 nm and 405 nm laser. The percentage of mCherry (for NHEJ-events) - and eGFP (for HR-events)-positive cells in the BFP-positive cell fraction was used for analysis.

Reference List

1. Pereira B, Chin SF, Rueda OM, Vollan HK, Provenzano E, Bardwell HA, et al. The somatic mutation profiles of 2,433 breast cancers refines their genomic and transcriptomic landscapes. Nature communications. 2016;7:11479.
2. Cerami E, Gao J, Dogrusoz U, Gross BE, Sumer SO, Aksoy BA, et al. The cBio cancer genomics portal: an open platform for exploring multidimensional cancer genomics data. Cancer discovery. 2012;2(5):401-4.
3. Gao J, Aksoy BA, Dogrusoz U, Dresdner G, Gross B, Sumer SO, et al. Integrative analysis of complex cancer genomics and clinical profiles using the cBioPortal. Science signaling. 2013;6(269):pl1.
4. Carter SL, Eklund AC, Kohane IS, Harris LN, Szallasi Z. A signature of chromosomal instability inferred from gene expression profiles predicts clinical outcome in multiple human cancers. Nature genetics. 2006;38(9):1043-8.
5. Burrell RA, McClelland SE, Endesfelder D, Groth P, Weller MC, Shaikh N, et al. Replication stress links structural and numerical cancer chromosomal instability. Nature. 2013;494(7438):492-6.
6. Birkbak NJ, Eklund AC, Li Q, McClelland SE, Endesfelder D, Tan P, et al. Paradoxical relationship between chromosomal instability and survival outcome in cancer. Cancer research. 2011;71(10):3447-52.
7. Buccitelli C, Salgueiro L, Rowald K, Sotillo R, Mardin BR, Korbel JO. Pan-cancer analysis distinguishes transcriptional changes of aneuploidy from proliferation. Genome research. 2017;27(4):501-11.
8. Pitroda SP, Bao R, Andrade J, Weichselbaum RR, Connell PP. Low Recombination Proficiency Score (RPS) Predicts Heightened Sensitivity to DNA-Damaging Chemotherapy in Breast Cancer. Clin Cancer Res. 2017;23(15):4493-500.
9. Werner S, Brors B, Eick J, Marques E, Pogenberg V, Parret A, et al. Suppression of early hematogenous dissemination of human breast cancer cells to bone marrow by retinoic Acid-induced 2. Cancer discovery. 2015;5(5):506-19.
10. Schneider CA, Rasband WS, Eliceiri KW. NIH Image to ImageJ: 25 years of image analysis. Nature methods. 2012;9(7):671-5.
11. Schindelin J, Arganda-Carreras I, Frise E, Kaynig V, Longair M, Pietzsch T, et al. Fiji: an open-source platform for biological-image analysis. Nature methods. 2012;9(7):676-82.
12. Ellinger B, Silber J, Prashar A, Landskron J, Weber J, Rehermann S, et al. A phenotypic screening approach to identify anticancer compounds derived from marine fungi. Assay Drug Dev Technol. 2014;12(3):162-75.
13. Neuhoff V, Arold N, Taube D, Ehrhardt W. Improved staining of proteins in polyacrylamide gels including isoelectric focusing gels with clear background at nanogram sensitivity using Coomassie Brilliant Blue G-250 and R-250. Electrophoresis. 1988;9(6):255-62.
14. Shevchenko A, Wilm M, Vorm O, Mann M. Mass spectrometric sequencing of proteins silver-stained polyacrylamide gels. Analytical chemistry. 1996;68(5):850-8.
15. Tyanova S, Temu T, Cox J. The MaxQuant computational platform for mass spectrometry-based shotgun proteomics. Nat Protoc. 2016;11(12):2301-19.
16. Certo MT, Ryu BY, Annis JE, Garibov M, Jarjour J, Rawlings DJ, et al. Tracking genome engineering outcome at individual DNA breakpoints. Nature methods. 2011;8(8):671-6.
