# Supplementary material for: Retinoic acid-induced 2 deficiency impairs genomic stability in breast cancer
Source: Breast Cancer Res. 2025 Jul 22;27:137. doi: 10.1186/s13058-025-02085-8 (PMC12285165; doi:10.1186/s13058-025-02085-8)
Supplement: Supplementary file 2 — Supplementary Material 2 [file 13058_2025_2085_MOESM2_ESM.pdf]

Supplementary Table S1: Rigor Adherence Table

**Ethics**
For clinical validation we used two breast cancer data sets provided by the cBIO portal for cancer genomics. TCGA Ethics and Policies was originally published by the National Cancer Institute.
**Inclusion and Exclusion Criteria**
Not required.
**Attrition**
Not required.
**Sex as a biological variable**
This is a study of breast cancer, which primarily affects women. The METABRIC data do not include data from male subjects. The TCGA dataset (n=1085) analyzed includes data from 12 male individuals. Gender was not considered as a biological variable in this study.
**Subject Demographics**
Not required.
**Randomization**
Not required.
**Blinding**
Not required.
**Power Analysis**
Not required.
**Replication**
All results from experiments performed in preclinical cell line models represent the mean of three independent biological replicates. Image analysis is based on single experiments and includes evaluation of at least 100 relevant cells or events.
**Cell Line Authentication**
Cell line authentication was performed using short tandem repeat profiling to exclude cross-contamination between cell lines. Cell lines were tested monthly for mycoplasma contamination.
**Data Information**
Microarray data are available at ArrayExpress accession no. E-MTAB-7071
