# Supplementary material for: Retinoic acid-induced 2 deficiency impairs genomic stability in breast cancer
Source: Breast Cancer Res. 2025 Jul 22;27:137. doi: 10.1186/s13058-025-02085-8 (PMC12285165; doi:10.1186/s13058-025-02085-8)
Supplement: Supplementary file 3 — Supplementary Material 3 [file 13058_2025_2085_MOESM3_ESM.pdf]

Supplementary Table S2: Key Resources Table

Reagent or Resource	Source	Identifier
**Antibodies**		
RAI2	Cell Signaling Technology	RRID:AB_2800292
Aurora A	Cell Signaling Technology	RRID:AB_2665504
Aurora B	Cell Signaling Technology	RRID:AB_10695307
Cyclin A2	Cell Signaling Technology	RRID:AB_627334
Cyclin B1	Cell Signaling Technology	RRID:AB_2783553
Cyclin B2	Santa Cruz Biotechnologies	RRID:AB_2072392
Survivin	Cell Signaling Technology	RRID:AB_2063948
HSC-70	Santa Cruz Biotechnologies	RRID:AB_627761
P-H3(S10)	Cell Signaling Technology	RRID:AB_1549592
CtBP1	BD Bioscience	RRID:AB_399429
Poly-(ADP-ribose)	Santa Cruz Biotechnologies	RRID:AB_785249
γH2AX	Cell Signaling Technology	RRID:AB_2118009
53BP1	Santa Cruz Biotechnologies	RRID:AB_2921289
Anti-centrosome	Millipore	RRID:AB_212756
Anti-rabbit IgG, HRP-linked	Cell Signaling Technology	RRID:AB_2099233
Anti-mouse IgG, HRP-linked	Cell Signaling Technology	RRID:AB_330924
Anti-rabbit IgG, IRDye 800CW	Licor	RRID:AB_621843
Anti-mouse IgG, IRDye 680RD	Licor	RRID:AB_10956588
Alexa Fluor 488 goat anti-rabbit IgG	Invitrogen	RRID:AB_143165
Alexa Fluor 546 goat anti-mouse IgG	Invitrogen	RRID:AB_2534093
**Experimental Models: Cell Lines**		
MCF-7	ATCC	RRID:CVCL_0031
KPL-1	DSMZ	RRID:CVCL_2094
CAMA-1	ATCC	RRID:CVCL_1115
MCF-10A	ATCC	RRID:CVCL_0598
293T	ATCC	CRL-3216
**Recombinant DNA**		
Non-target shRNA	Sigma-Aldrich	SHC016
shRNA1	Sigma-Aldrich	TRCN0000139927
shRNA2	Sigma-Aldrich	TRCN0000441623
pH2B-eYFP	Addgene	Plasmid #51002
phCMV3-RAI2-HA	Self-made	PMID:25716347
BFP-TLR-SceI	Addgene	Plasmid #31481
GFP donor	Addgene	Plasmid #31475
**Software and Algorithms**		
Bioconductor		RRID:SCR_006442
DAVID		RRID:SCR_001881
FACSDiva	BD Bioscience	RRID:SCR_001456
Image Studio Lite	Licor	RRID:SCR_013715
ImageJ		RRID:SCR_003070
MaxQuant software		RRID:SCR_014485
QIAGEN GeneGlobe Data Analysis Center	Qiagen	RRID:SCR_021211
Imaris imaging software	Oxford Instruments	RRID:SCR_007370
