# Supplementary material for: Retinoic acid-induced 2 deficiency impairs genomic stability in breast cancer
Source: Breast Cancer Res. 2025 Jul 22;27:137. doi: 10.1186/s13058-025-02085-8 (PMC12285165; doi:10.1186/s13058-025-02085-8)
Supplement: Supplementary file 4 — Supplementary Material 4 [file 13058_2025_2085_MOESM4_ESM.pdf]

Supplementary Table S4: Results of gene ontology enrichment analysis of significantly deregulated genes in RAI2-depleted KPL-1 cells

**GO-TERMS Biological Process**												
Category	Term	Count	%	PValue	Genes	List Total	Pop Hits	Pop Total	Fold Enrichment	Bonferroni	Benjamini	FDR
GOTERM_BP_DIRECT	GO:0051301~cell division	29	17,90123	5,08E-19	KIFC1, CKS1B, PTTG1, SPC24, CCNE2, FAM83D, KIF2C, CDCA8, OIP5, NCAPG, SEH1L, BUB1, FBXO5, CCNA2, CDCA5, HELLS, CDCA3, KIF14, KIF11, CDK6, BIRC5, CDC20, CENPE, TACC3, UBE2C, CCNB2, MAD2L1, ZWINT, KIF20B	151	350	16792	9,214153	5,16E-16	5,16E-16	8,04E-16
GOTERM_BP_DIRECT	GO:0007067~mitotic nuclear division	22	13,58025	6,43E-15	KIF22, KIF11, BIRC5, CDC20, ANLN, PBK, PTTG1, AURKB, CEP55, SPC24, FAM83D, KIF2C, CCNB2, OIP5, BUB1, KIF20B, FBXO5, CDCA5, CCNA2, ASPM, HELLS, CDCA3	151	248	16792	9,864986	6,54E-12	3,27E-12	1,02E-11
GOTERM_BP_DIRECT	GO:0007062~sister chromatid cohesion	16	9,876543	1,89E-14	KIF22, CENPM, BIRC5, CDC20, CENPE, AURKB, CENPK, SPC24, KIF2C, CDCA8, MAD2L1, SEH1L, CENPA, ZWINT, BUB1, CDCA5	151	103	16792	17,27461	1,92E-11	6,39E-12	2,99E-11
GOTERM_BP_DIRECT	GO:0007080~mitotic metaphase plate congression	9	5,555556	1,04E-09	KIF14, KIFC1, KIF22, KIF2C, CDCA8, SEH1L, CENPE, CEP55, CDCA5	151	37	16792	27,04994	1,06E-06	2,64E-07	1,65E-06
GOTERM_BP_DIRECT	GO:0007018~microtubule-based movement	10	6,17284	4,29E-08	KIF23, KIF14, KIFC1, KIF22, KIF2C, KIF4A, KIF11, KIF20B, CENPE, KIF20A	151	81	16792	13,72905	4,36E-05	8,72E-06	6,8E-05
GOTERM_BP_DIRECT	GO:0006890~retrograde vesicle-mediated transport, Golgi to ER	7	4,320988	9,2E-05	KIF23, KIF22, KIF2C, KIF4A, KIF11, TMED10, CENPE	151	82	16792	9,493135	0,089202	0,010328	0,145612
GOTERM_BP_DIRECT	GO:0051310~metaphase plate congression	4	2,469136	0,000145	FAM83D, KIF22, KIF2C, CENPE	151	12	16792	37,06843	0,136765	0,014599	0,229101
GOTERM_BP_DIRECT	GO:0019886~antigen processing and presentation of	7	4,320988	0,000174	KIF23, KIF22, KIF2C, KIF4A, KIF11, CTSD, CENPE	151	92	16792	8,461273	0,162102	0,015949	0,275444

	exogenous peptide antigen via MHC class II											
GOTERM_BP_DIRECT	GO:0000731~DNA synthesis involved in DNA repair	4	2,469136	0,003709	EXO1, RFC3, RAD51AP1, BARD1	151	35	16792	12,70918	0,97698	0,164387	5,712111
GOTERM_BP_DIRECT	GO:0006281~DNA repair	8	4,938272	0,005219	EXO1, KIF22, UHRF1, RAD51AP1, BTG2, FANCI, FOXM1, PTTG1	151	235	16792	3,785712	0,995064	0,206199	7,949483
GOTERM_BP_DIRECT	GO:0031145~anaphase-promoting complex-dependent catabolic process	5	3,08642	0,005472	MAD2L1, CDC20, PTTG1, AURKB, UBE2C	151	79	16792	7,03831	0,996188	0,207105	8,31956
GOTERM_BP_DIRECT	GO:0042787~protein ubiquitination involved in ubiquitin-dependent protein catabolic process	6	3,703704	0,012117	UHRF1, MAD2L1, CDC20, PTTG1, AURKB, UBE2C	151	153	16792	4,360992	0,999996	0,357198	17,54992
GOTERM_BP_DIRECT	GO:0006260~DNA replication	6	3,703704	0,012758	EXO1, GINS2, RFC3, KIAA0101, BARD1, DUT	151	155	16792	4,304721	0,999998	0,361986	18,39269
GOTERM_BP_DIRECT	GO:0000082~G1/S transition of mitotic cell cycle	5	3,08642	0,01323	CCNE2, FBXO5, CDK6, CDCA5, ITGB1	151	102	16792	5,45124	0,999999	0,362748	19,0079
GOTERM_BP_DIRECT	GO:0097503~sialylation	3	1,851852	0,013554	ST3GAL1, ST6GAL1, ST3GAL5	151	20	16792	16,68079	0,999999	0,360331	19,42774
GOTERM_BP_DIRECT	GO:0051439~regulation of ubiquitin-protein ligase activity involved in mitotic cell cycle	3	1,851852	0,017736	FBXO5, CDC20, UBE2C	151	23	16792	14,50504	1	0,413867	24,66815
GOTERM_BP_DIRECT	GO:0009311~oligosaccharide metabolic process	3	1,851852	0,020788	ST3GAL1, ST6GAL1, ST3GAL5	151	25	16792	13,34464	1	0,45621	28,28919
GOTERM_BP_DIRECT	GO:0051436~negative regulation of ubiquitin-protein ligase activity involved in mitotic cell cycle	4	2,469136	0,025673	MAD2L1, FBXO5, CDC20, UBE2C	151	71	16792	6,265087	1	0,491798	33,74763
GOTERM_BP_DIRECT	GO:0051437~positive regulation of ubiquitin-protein ligase activity involved in regulation of mitotic cell cycle transition	4	2,469136	0,030584	MAD2L1, FBXO5, CDC20, UBE2C	151	76	16792	5,85291	1	0,545325	38,84053
GOTERM_BP_DIRECT	GO:0018279~protein N-linked glycosylation via asparagine	3	1,851852	0,049568	ST3GAL1, ST6GAL1, ST3GAL5	151	40	16792	8,340397	1	0,698814	55,28043
GOTERM_BP_DIRECT	GO:0006486~protein glycosylation	4	2,469136	0,080587	ST3GAL1, ST6GAL1, GALNT10, ST3GAL5	151	113	16792	3,936471	1	0,837074	73,55228

GOTERM_BP_DIRECT	GO:0006334~nucleosome assembly	4	2,469136	0,090768	HMGB2, HIST1H2BK, CENPA, HIST1H4C	151	119	16792	3,737993	1	0,860692	77,82654
GOTERM_BP_DIRECT	GO:1901796~regulation of signal transduction by p53 class mediator	4	2,469136	0,099641	EXO1, RFC3, AURKB, BARD1	151	124	16792	3,587268	1	0,881249	81,01483
GOTERM_BP_DIRECT	GO:0016266~O-glycan processing	3	1,851852	0,100378	ST3GAL1, ST6GAL1, GALNT10	151	60	16792	5,560265	1	0,878182	81,25917
GOTERM_BP_DIRECT	GO:0048208~COPII vesicle coating	3	1,851852	0,103196	CD59, TMED10, SERPINA1	151	61	16792	5,469113	1	0,880685	82,16727
GOTERM_BP_DIRECT	GO:0006468~protein phosphorylation	8	4,938272	0,114637	ST3GAL1, PLK4, MAPK4, BUB1, BIRC5, CDK6, PBK, AURKB	151	456	16792	1,95097	1	0,898591	85,44714
GOTERM_BP_DIRECT	GO:0046777~protein autophosphorylation	4	2,469136	0,199107	EPHA4, TTK, AURKB, MELK	151	172	16792	2,58617	1	0,976622	97,02415
GOTERM_BP_DIRECT	GO:0043161~proteasome-mediated ubiquitin-dependent protein catabolic process	4	2,469136	0,272432	KIF14, MAD2L1, CDC20, UBE2C	151	203	16792	2,191237	1	0,992488	99,34912
GOTERM_BP_DIRECT	GO:0016567~protein ubiquitination	5	3,08642	0,399691	BIRC5, FBXO4, UBE2C, CDCA3, BARD1	151	359	16792	1,54882	1	0,999321	99,96897
GOTERM_BP_DIRECT	GO:0006888~ER to Golgi vesicle-mediated transport	3	1,851852	0,419423	CD59, TMED10, SERPINA1	151	160	16792	2,085099	1	0,999479	99,98172
GOTERM_BP_DIRECT	GO:0098609~cell-cell adhesion	4	2,469136	0,437081	S100P, CCNB2, ANLN, RDX	151	271	16792	1,641407	1	0,999622	99,98879
GOTERM_BP_DIRECT	GO:0000122~negative regulation of transcription from RNA polymerase II promoter	8	4,938272	0,464454	N4BP2L2, UHRF1, BTG2, FOXM1, HOPX, NR4A2, FHL2, AURKB	151	720	16792	1,235614	1	0,999761	99,99491
GOTERM_BP_DIRECT	GO:0006508~proteolysis	5	3,08642	0,656904	PRSS8, KLK5, MME, CTSD, PRSS22	151	500	16792	1,112053	1	0,999998	100
GOTERM_BP_DIRECT	GO:0015031~protein transport	4	2,469136	0,688953	SEH1L, ZMAT3, MVP, KIF20A	151	395	16792	1,12613	1	0,999999	100
GOTERM_BP_DIRECT	GO:0045944~positive regulation of transcription from RNA polymerase II promoter	6	3,703704	0,942637	UHRF1, HMGB2, EPAS1, FOXM1, NR4A2, TOP2A	151	981	16792	0,680155	1	1	100
GOTERM_BP_DIRECT	GO:0006351~transcription, DNA-templated	12	7,407407	0,969232	UHRF1, HMGB2, BTG2, EPAS1, FOXM1, HOPX, NR4A2, FHL2, RORC, BIRC5, TP53INP2, HELLS	151	1955	16792	0,68259	1	1	100
**GO-TERMS Cellular Compartment**												
Category	Term	Count	%	PValue	Genes	List Total	Pop Hits	Pop Total	Fold Enrichment	Bonferroni	Benjamini	FDR
GOTERM_CC_DIRECT	GO:0005871~kinesin complex	10	6,172839506	7,44731E-10	KIF23, KIF14, KIFC1, KIF22, KIF2C, KIF4A,	160	53	18224	21,49056604	1,83948E-07	1,83948E-07	9,65639E-07

					KIF11, KIF20B, CENPE, KIF20A							
GOTERM_CC_DIRECT	GO:0030496~midbody	13	8,024691358	1,3911E-09	KIF23, KIF14, KIF4A, CDCA8, PRC1, KIF20B, BIRC5, RDX, CENPE, CEP55, AURKB, ASPM, KIF20A	160	129	18224	11,47829457	3,43601E-07	1,71801E-07	1,80374E-06
GOTERM_CC_DIRECT	GO:0000777~condensed chromosome kinetochore	11	6,790123457	4,32338E-09	SPC24, KIF2C, CENPM, MAD2L1, SEH1L, HJURP, ZWINT, BUB1, CENPE, BIRC5, CENPK	160	87	18224	14,40114943	1,06787E-06	3,55958E-07	5,60582E-06
GOTERM_CC_DIRECT	GO:0005819~spindle	11	6,790123457	1,06121E-07	KIF23, KIFC1, KIF11, PRC1, NUSAP1, FBXO5, TTK, BIRC5, CDC20, AURKB, KIF20A	160	121	18224	10,35454545	2,62114E-05	5,24234E-06	0,000137599
GOTERM_CC_DIRECT	GO:0000776~kinetochore	9	5,555555556	5,27574E-07	KIF22, KIF2C, MAD2L1, SEH1L, ZWINT, BUB1, TTK, CENPE, AURKB	160	81	18224	12,65555556	0,000130302	2,17182E-05	0,000684066
GOTERM_CC_DIRECT	GO:0005874~microtubule	13	8,024691358	1,87949E-05	KIF14, KIF23, KIF22, KIFC1, KIF4A, KIF11, NUSAP1, CENPE, BIRC5, KIF2C, KIF20B, TUBA4A, KIF20A	160	311	18224	4,761093248	0,004631616	0,000515686	0,02436721
GOTERM_CC_DIRECT	GO:0005876~spindle microtubule	6	3,703703704	3,91651E-05	KIF4A, KIF11, PRC1, NUSAP1, BIRC5, AURKB	160	44	18224	15,53181818	0,009627328	0,000879065	0,050770681
GOTERM_CC_DIRECT	GO:0005777~peroxisome	4	2,469135802	0,062757819	ACOX2, SERHL2, PXMP4, CROT	160	104	18224	4,380769231	0,999999888	0,486774325	56,84582211
GOTERM_CC_DIRECT	GO:0005615~extracellular space	18	11,11111111	0,079402181	ACTB, HMGB2, ENPP1, MSMB, KLK5, RDX, TIMP2, PRSS8, HIST1H2BK, CPE, ADM, GSN, CD59, CTSD, HBEGF, SERPINA1, PCSK6, AGR2	160	1347	18224	1,522048998	0,999999999	0,493970478	65,79254626
GOTERM_CC_DIRECT	GO:0030173~integral component of Golgi membrane	3	1,851851852	0,091130056	ST3GAL1, ST6GAL1, ST3GAL5	160	58	18224	5,89137931	1	0,510906574	71,03177404
GOTERM_CC_DIRECT	GO:0033116~endoplasmic reticulum-Golgi intermediate compartment membrane	3	1,851851852	0,107520702	CD59, TMED10, SERPINA1	160	64	18224	5,3390625	1	0,541807025	77,12075354
GOTERM_CC_DIRECT	GO:0000139~Golgi membrane	9	5,555555556	0,145896098	ST3GAL1, ST6GAL1, GALNT10, MALL, ST3GAL5, CD59, TMED10, HS6ST2, SERPINA1	160	591	18224	1,734517766	1	0,631672222	87,05957531
GOTERM_CC_DIRECT	GO:0000786~nucleosome	3	1,851851852	0,197994367	HIST1H2BK, CENPA, HIST1H4C	160	94	18224	3,635106383	1	0,702120582	94,27813743

GOTERM_CC_DIRECT	GO:0005913~cell-cell adherens junction	5	3,086419753	0,311522748	S100P, CCNB2, ANLN, RDX, ITGB1	160	323	18224	1,763157895	1	0,841811612	99,20926522
GOTERM_CC_DIRECT	GO:0016021~integral component of membrane	32	19,75308642	0,996073838	ENPP5, C15ORF48, CYB5R1, MPZL2, ENPP1, VTCN1, MME, ITM2A, PRSS8, ST3GAL1, GALNT10, MALL, ST3GAL5, SHISA2, PXMP4, PEMT, HS6ST2, TMED10, FNDC3B, SASH1, SPNS2, HERPUD1, ST6GAL1, OSTM1, EPHA4, KCNN4, TLCD1, UCP2, ATP2A3, MS4A10, ATP6V0A4, SSR2	160	5163	18224	0,705946155	1	0,999999997	100
Category	Term	Count	%	PValue	Genes	List Total	Pop Hits	Pop Total	Fold Enrichment	Bonferroni	Benjamini	FDR
GOTERM_CC_DIRECT	GO:0005871~kinesin complex	10	6,172839506	7,44731E-10	KIF23, KIF14, KIFC1, KIF22, KIF2C, KIF4A, KIF11, KIF20B, CENPE, KIF20A	160	53	18224	21,49056604	1,83948E-07	1,83948E-07	9,65639E-07
**GO-TERMS Molecular Function**												
Category	Term	Count	%	PValue	Genes	List Total	Pop Hits	Pop Total	Fold Enrichment	Bonferroni	Benjamini	FDR
GOTERM_MF_DIRECT	GO:0003777~microtubule motor activity	10	6,172839506	4,36706E-08	KIF23, KIF14, KIFC1, KIF22, KIF2C, KIF4A, KIF11, KIF20B, CENPE, KIF20A	154	80	16881	13,70211039	1,2184E-05	6,09204E-06	5,7711E-05
GOTERM_MF_DIRECT	GO:0008017~microtubule binding	13	8,024691358	4,30463E-07	KIF23, KIF14, FAM83D, KIFC1, KIF22, KIF4A, KIF11, PRC1, KIF20B, NUSAP1, BIRC5, CENPE, KIF20A	154	208	16881	6,851055195	0,000120092	4,00323E-05	0,000568858
GOTERM_MF_DIRECT	GO:0008017~microtubule binding	13	8,024691358	4,30463E-07	KIF23, KIF14, FAM83D, KIFC1, KIF22, KIF4A, KIF11, PRC1, KIF20B, NUSAP1, BIRC5, CENPE, KIF20A	154	208	16881	6,851055195	0,000120092	4,00323E-05	0,000568858
GOTERM_MF_DIRECT	GO:0005524~ATP binding	29	17,90123457	0,00016859	KIF23, KIFC1, KIF22, KIF4A, ENPP1, TTK, AURKB, TK1, TPK1, KIF2C, BUB1, TOP2A, HELLS, TRIP13, ACTB, KIF14, KIF11, CDK6, CENPE, PBK, ACACB, UBE2C,	154	1495	16881	2,126347565	0,045951416	0,009364018	0,222563905

					EPHA4, PLK4, ATP2A3, MAPK4, KIF20B, MELK, KIF20A							
GOTERM_MF_DIRECT	GO:0016887~ATPase activity	9	5,555555556	0,000266052	KIF23, KIF14, KIFC1, KIF22, KIF2C, RFC3, KIF20B, CENPE, KIF20A	154	183	16881	5,390994252	0,071549781	0,012296854	0,351019132
GOTERM_MF_DIRECT	GO:0008574~ATP-dependent microtubule motor activity, plus-end-directed	4	2,469135802	0,000452218	KIF14, KIF4A, KIF11, KIF20B	154	17	16881	25,79220779	0,118559129	0,017866658	0,595960925
GOTERM_MF_DIRECT	GO:0008373~sialyltransferase activity	3	1,851851852	0,012613866	ST3GAL1, ST6GAL1, ST3GAL5	154	19	16881	17,30792891	0,971034588	0,325320141	15,44375193
GOTERM_MF_DIRECT	GO:0008236~serine-type peptidase activity	3	1,851851852	0,111546344	PRSS8, KLK5, PRSS22	154	63	16881	5,219851577	1	0,905296829	79,04895769
GOTERM_MF_DIRECT	GO:0004674~protein serine/threonine kinase activity	7	4,320987654	0,127233049	PLK4, MAPK4, BUB1, TTK, PBK, AURKB, MELK	154	376	16881	2,040739845	1	0,920437533	83,44353865
GOTERM_MF_DIRECT	GO:0004252~serine-type endopeptidase activity	5	3,086419753	0,201192031	PRSS8, KLK5, CTSD, PCSK6, PRSS22	154	255	16881	2,149350649	1	0,969249157	94,86227881
GOTERM_MF_DIRECT	GO:0004672~protein kinase activity	6	3,703703704	0,226927955	EPHA4, HSPB8, BUB1, PBK, AURKB, MELK	154	359	16881	1,832037044	1	0,977165132	96,66711417
GOTERM_MF_DIRECT	GO:0098641~cadherin binding involved in cell-cell adhesion	5	3,086419753	0,269085625	S100P, CCNB2, ANLN, RDX, ITGB1	154	290	16881	1,889946261	1	0,981225009	98,41149723
GOTERM_MF_DIRECT	GO:0004842~ubiquitin-protein transferase activity	5	3,086419753	0,348619228	UHRF1, BIRC5, FBXO4, UBE2C, BARD1	154	329	16881	1,665910078	1	0,989946769	99,6534085
GOTERM_MF_DIRECT	GO:0003779~actin binding	4	2,469135802	0,462905987	GSN, ANLN, RDX, ITGB1	154	278	16881	1,57722134	1	0,997471297	99,97292308
GOTERM_MF_DIRECT	GO:0008270~zinc ion binding	12	7,407407407	0,493924262	PEG10, UHRF1, CPE, ENPP1, ZMAT3, NR4A2, FHL2, MME, RORC, BIRC5, TK1, BARD1	154	1169	16881	1,125237466	1	0,997822593	99,98766353
GOTERM_MF_DIRECT	GO:0043565~sequence-specific DNA binding	4	2,469135802	0,852605847	EPAS1, FOXM1, NR4A2, RORC	154	518	16881	0,846462418	1	0,999998874	100
