# Supplementary material for: Retinoic acid-induced 2 deficiency impairs genomic stability in breast cancer
Source: Breast Cancer Res. 2025 Jul 22;27:137. doi: 10.1186/s13058-025-02085-8 (PMC12285165; doi:10.1186/s13058-025-02085-8)
Supplement: Supplementary file 5 — Supplementary Material 5 [file 13058_2025_2085_MOESM5_ESM.pdf]

Supplementary Table S5: Validation of significantly deregulated genes in RAI2-depleted breast cancer cell lines by multiplex qRT-PCR analysis. FC: fold change

Gene Symbol	FC KPL-1	P-value KPL-1	FC CAMA-1	P-value CAMA-1	FC MCF-7	P-value MCF-7	FC MCF-10A	P-value MCF-10A
**ABL1**	0,38510	0,04021	0,79740	0,08384	0,45690	0,00001	0,83120	0,15034
**ANAPC2**	0,61130	0,29736	0,76490	0,05884	0,84090	0,02812	0,78280	0,16110
**ATM**	0,68300	0,06562	0,65370	0,02773	0,54150	0,01179	0,76840	0,05035
**ATR**	1,35970	0,93193	0,72530	0,00402	0,65750	0,00862	0,64770	0,02919
**AURKA**	0,21810	0,00874	0,37280	0,00533	0,74230	0,00917	0,52360	0,00326
**AURKB**	0,06180	0,00688	0,31060	0,00236	0,55290	0,00002	0,48300	0,00180
**BCCIP**	1,76130	0,96773	0,72700	0,00078	0,70710	0,00075	0,59190	0,00200
**BCL2**	0,45690	0,02820	0,73540	0,04010	0,50870	0,00120	0,63880	0,04105
**BIRC5**	0,12940	0,01191	0,41080	0,01257	0,51410	0,08929	0,50230	0,00448
**BRCA1**	0,15040	0,00416	0,36520	0,00192	0,81790	0,00054	0,65520	0,00819
**BRCA2**	0,15110	0,00734	0,40050	0,00316	0,52300	0,00001	0,58100	0,04884
**CASP3**	0,44650	0,09347	0,89920	0,40219	0,62630	0,00087	0,93950	0,62201
**CCNA2**	0,10340	0,00499	0,40240	0,00006	0,69260	0,00553	0,47080	0,00272
**CCNB1**	0,21320	0,00833	0,34550	0,00005	0,65290	0,00067	0,46980	0,00095
**CCNB2**	0,11580	0,00551	0,41660	0,00005	0,67830	0,00973	0,48860	0,00052
**CCNC**	0,70710	0,03283	0,63000	0,00013	0,53590	0,00171	0,64470	0,06049
**CCND1**	1,25700	0,52859	0,48300	0,01606	0,63070	0,00347	0,87260	0,29172
**CCND2**	1,27750	0,34342	0,87460	0,07131	0,40710	0,00000	0,75090	0,11944
**CCND3**	0,36270	0,01726	0,57300	0,02079	0,76840	0,01295	0,74230	0,10282
**CCNE1**	0,46540	0,05566	0,57570	0,00318	0,83510	0,07074	0,57830	0,00462
**CCNF**	0,18470	0,01047	0,40900	0,01595	0,83800	0,02542	0,56120	0,02884
**CCNG1**	1,01630	0,86003	0,58100	0,00451	0,73970	0,00245	0,73880	0,00507
**CCNG2**	0,69900	0,22113	1,27460	0,00548	0,49310	0,00007	0,85460	0,16864
**CCNH**	0,76310	0,19944	0,68620	0,00186	0,53960	0,00391	0,57430	0,00464
**CCNT1**	0,49650	0,04124	0,83120	0,05818	0,90750	0,02336	0,79920	0,05844
**CDC16**	0,54840	0,01776	0,79190	0,00219	0,56060	0,00001	0,73880	0,02730
**CDC20**	0,09150	0,00871	0,30990	0,00079	0,59870	0,00023	0,44540	0,00089
**CDC25A**	0,20540	0,01330	0,43530	0,01095	0,71450	0,03953	0,51760	0,00068
**CDC25C**	0,08920	0,00861	0,37540	0,00011	0,64620	0,02535	0,58370	0,01035
**CDC34**	0,38510	0,02042	0,78820	0,33381	0,65980	0,00182	0,86250	0,08561
**CDC6**	0,13210	0,00434	0,30920	0,00046	0,63290	0,00125	0,44750	0,00046
**CDK1**	0,12190	0,03212	0,36860	0,00010	0,52490	0,00012	0,45900	0,00167
**CDK2**	0,30430	0,01069	0,44240	0,00461	0,68540	0,00107	0,67520	0,00475
**CDK4**	0,23820	0,00738	0,50700	0,00656	0,71200	0,00166	0,52850	0,00142
**CDK5R1**	0,41850	0,03527	0,48750	0,00272	0,41180	0,00118	0,79550	0,02278
**CDK5RAP1**	0,45380	0,04117	0,60850	0,00128	0,61130	0,00015	0,65820	0,02574
**CDK6**	1,04010	0,78868	7,96310	0,00017	1,52100	0,01507	0,93740	0,53447
**CDK7**	0,70060	0,07730	0,82360	0,06218	0,71950	0,00005	0,67670	0,01190
**CDK8**	0,68140	0,11731	0,78460	0,03719	0,69740	0,11614	0,70870	0,00650
**CDKN1A**	1,82340	0,33593	7,07800	0,00001	0,59870	0,00257	0,91380	0,50676
**CDKN1B**	0,45270	0,05184	0,85860	0,36498	0,71950	0,00770	0,78280	0,11308
**CDKN2A**	0,86060	0,94112	0,78640	0,11585	0,39230	0,00000	1,03770	0,79996

**CDKN2B**	0,75440	0,06541	3,53080	0,00152	0,37370	0,00031	1,03770	0,79996
**CDKN3**	0,24710	0,01295	0,48630	0,00078	0,91380	0,01016	0,57430	0,00103
**CHEK1**	0,20030	0,02956	0,44140	0,00052	0,65980	0,00165	0,67670	0,00910
**CHEK2**	0,80110	0,42438	0,39590	0,00240	0,79830	0,00003	0,63290	0,00595
**CKS1B**	0,26610	0,00314	0,41660	0,00001	0,55290	0,00000	0,61420	0,00108
**CKS2**	0,42340	0,05262	0,32990	0,00061	0,54340	0,00021	0,57430	0,01154
**CUL1**	0,45590	0,00942	0,72700	0,01241	0,77380	0,00005	0,75790	0,05759
**CUL2**	0,65670	0,00388	0,61840	0,00006	0,52670	0,00009	0,65220	0,00220
**CUL3**	0,81410	0,18109	0,65370	0,00021	0,61130	0,00003	0,71530	0,00331
**E2F1**	0,12790	0,00356	0,46330	0,01145	0,61770	0,00000	0,60850	0,00209
**E2F4**	0,19800	0,00167	0,41950	0,01700	0,56060	0,00030	0,58240	0,01191
**GADD45A**	0,56120	0,09585	1,41420	0,06541	0,85260	0,08812	0,73200	0,00283
**GTSE1**	0,10110	0,00630	0,33290	0,00143	0,47800	0,00285	0,44750	0,00002
**HUS1**	0,59320	0,10086	0,77380	0,25345	0,81230	0,00862	0,75790	0,01150
**KNTC1**	0,32840	0,06750	0,43930	0,00014	0,68540	0,00006	0,66590	0,01056
**KPNA2**	0,30150	0,00794	0,50460	0,01238	0,71700	0,00380	0,59050	0,00097
**MAD2L1**	3,23400	0,28431	0,33450	0,00036	0,53220	0,00000	0,37280	0,00125
**MAD2L2**	0,39780	0,00506	0,46440	0,00454	0,47630	0,00009	0,81980	0,01233
**MCM2**	0,11800	0,00922	0,31790	0,00233	0,70710	0,02997	0,42730	0,00663
**MCM3**	0,21920	0,00564	0,36100	0,00172	0,55290	0,00757	0,48190	0,00061
**MCM4**	0,17800	0,00575	0,49430	0,01832	0,64390	0,00026	0,39050	0,00610
**MCM5**	0,21070	0,15630	0,41750	0,00171	0,81510	0,02068	0,60010	0,01457
**MDM2**	0,91170	0,53846	0,54590	0,00195	0,88580	0,10800	0,87860	0,31499
**MKI67**	0,11210	0,01884	0,28260	0,00076	0,65520	0,00209	0,45900	0,00073
**MNAT1**	0,71040	0,09788	0,78820	0,01175	0,46490	0,00008	0,60290	0,01543
**MRE11A**	1,25410	0,53814	0,60710	0,00755	0,70470	0,00444	0,66280	0,00631
**NBN**	0,49430	0,00387	0,70060	0,00720	0,61990	0,00000	0,63000	0,00433
**RAD1**	0,52850	0,00161	0,60150	0,00218	0,55100	0,00003	0,58510	0,00114
**RAD17**	0,74050	0,01377	0,65520	0,00686	0,62200	0,00000	0,81230	0,02573
**RAD51**	0,14160	0,00967	0,30920	0,00066	0,51580	0,00033	0,54970	0,01709
**RAD9A**	0,27800	0,02287	0,50000	0,01781	1,10570	0,03732	0,76310	0,01671
**RB1**	0,44240	0,00288	0,48750	0,00004	0,60290	0,00184	0,60430	0,00041
**RBBP8**	0,35930	0,00170	0,55990	0,00061	0,45690	0,00087	0,54090	0,01173
**RBL1**	0,20490	0,00729	0,39140	0,00218	0,80390	0,02414	0,58370	0,02027
**RBL2**	0,58370	0,04991	0,77560	0,04600	0,64620	0,00846	0,83700	0,05434
**SERTAD1**	0,52000	0,09123	0,97270	0,90253	0,42340	0,00476	0,81230	0,17915
**SKP2**	0,70870	0,36514	0,55990	0,00576	0,68540	0,00546	0,54840	0,01058
**STMN1**	0,20310	0,00277	0,42830	0,00007	0,52850	0,00051	0,55350	0,00311
**TFDP1**	0,27480	0,02731	0,39410	0,00259	0,68070	0,00028	0,53840	0,00114
**TFDP2**	0,55220	0,01449	0,84090	0,03123	0,71200	0,00184	0,74400	0,02065
**TP53**	0,27800	0,02940	0,60290	0,01780	0,63290	0,00000	0,49310	0,00262
**WEE1**	0,71200	0,28491	0,73040	0,02895	0,72950	0,00035	0,61700	0,02418
**ACTB**	0,41270	0,06515	0,95260	0,70495	1,16070	0,04867	0,73040	0,03640
**B2M**	1,00000	0,00000	1,00000	0,00000	1,00000	0,00000	1,00000	0,00000
