# Supplementary material for: Retinoic acid-induced 2 deficiency impairs genomic stability in breast cancer
Source: Breast Cancer Res. 2025 Jul 22;27:137. doi: 10.1186/s13058-025-02085-8 (PMC12285165; doi:10.1186/s13058-025-02085-8)
Supplement: Supplementary file 6 — Supplementary Material 6 [file 13058_2025_2085_MOESM6_ESM.pdf]

Supplementary Table S6: Results of cytotoxicity screening with a library of 1280 pharmacologically active compounds

Compound Id	growth [%], non target	growth [%], shRNA median	Objd ID	Name	Class	Action	Selectivity	Description
**LOP_0001342**	164,09	-73,72	LOP_0001342	Apomorphine hydrochloride hemihydrate	Dopamine	Agonist		Non-selective dopamine receptor agonist
**LOP_0001548**	131,82	-65,14	LOP_0001548	Aurora-A Inhibitor I	kinase phosphotase	Inhibitor	Aurora kinase A	Aurora-A Inhibitor I is a potent inhibitor of Aurora kinase A with an IC50 of 3.4 nM and 1000-fold selectivity for Aurora A over Aurora B
**LOP_0000699**	121,62	-22,98	LOP_0000699	Paroxetine hydrochloride hemihydrate (MW = 374.83)	Serotonin	Inhibitor	Reuptake	Selective serotonin reuptake inhibitor; antidepressant<br>Sold with permission of GlaxoSmithKline.<br>Actual molecular weight = 374.83 to account for 1/2 mole of water.
**LOP_0001377**	99,63	-36,13	LOP_0001377	SB743921 hydrochloride	Cell Cycle	inhibitor	KSP	SB743921 is an inhibitor of kinesin spindle protein (KSP) with Ki of 0.1 nM
**LOP_0001004**	97,67	-56,69	LOP_0001004	beta-Lapachone	Apoptosis	Activator		Induces apoptosis in HL-60 cells; anticancer agent
**LOP_0001484**	83,07	-20,26	LOP_0001484	SB 674042	Cytokines	Antagonist	OX1R	SB-674042 is potent and selective OX1R antagonist
**LOP_0000522**	81,49	-21,52	LOP_0000522	Metergoline	Serotonin	Antagonist	5-HT2/5-HT1D	5-HT1/5-HT2 Serotonin receptor antagonist; analgesic; antipyretic
**LOP_0001003**	78,1	-47,32	LOP_0001003	CyPPA	K+ Channels	Modulator	SK3 and SK2	CyPPA is a positive modulator of small conductance Ca2+-activated K2+ channels; selective for SK3 and SK2.
**LOP_0001401**	65,52	-28,25	LOP_0001401	Piperlongumine	Apoptosis	inhibitor	GSTP1	Piperlongumine selectively kills cancer cells regardless of p53 status without harming normal cells. It binds to and inihbits proteins known to regulate oxidative stress
**LOP_0001016**	62,85	-40,67	LOP_0001016	NNC 55-0396	Ca2+ Channel	Inhibitor	T-type	Selective T-type calcium channel inhibitor.
**LOP_0001060**	50,12	-24,3	LOP_0001060	MDL 28170	Cell Cycle	Inhibitor	Calpain I / II	Cell permeable calpain I and II inhibitor
**LOP_0001188**	40,57	-22,29	LOP_0001188	Rottlerin				
**LOP_0001084**	31,15	-24,09	LOP_0001084	PD-407824	Kinase	Inhibitor	Wee1/Chk1	PD-407824 is a Wee1/Chk1 inhibitor
**LOP_0000975**	29,66	-48,66	LOP_0000975	Idarubicin	DNA Metabolism	Inhibitor		Antineoplastic
**LOP_0001057**	23,07	-27,45	LOP_0001057	Mitoxantrone	DNA Metabolism	Inhibitor		DNA synthesis inhibitor
**LOP_0000881**	21,76	-28,89	LOP_0000881	Emetine dihydrochloride hydrate	Apoptosis	Activator		Apoptosis inducer; RNA-Protein translation inhibitor
**LOP_0001121**	15,69	-36,42	LOP_0001121	Topotecan hydrochloride hydrate	Apoptosis and Cell Cycle	Inhibitor	topoisomerase I	Topetecan is a topoisomerase I inhibitor and an apoptosis inducer. It is a potent antineoplastic agent
**LOP_0001392**	10,89	-40,72	LOP_0001392	TIC10 angular	Gene Regulation	Antagonist	Akt and ERK	TIC10 angular is a potent blood brain barrier penetrant and orally active inducer of TRAIL production that induces death of multiple human cancers
**LOP_0001373**	3,24	-42,16	LOP_0001373	Torin2	Kinase/Phosphotase	Inhibitor	mTOR	Torin2 is a highly potent and selective ATP-competitive mTOR inhibitor. Torin2 has an IC50 of 0.25 nM and 800-fold greater selectivity for mTOR than PI3K.
**LOP_0000458**	-3,07	-29,94	LOP_0000458	Gossypol	Apoptosis	Inducer		Natural product from cotton seeds with a variety of cell biological activities. Proapoptotic, antimalarial, PKC inhibition.
**LOP_0001215**	-12,03	-21,37	LOP_0001215	Stattic	Gene Regulation	Inhibitor	STAT3	Irreversible STAT3 activation inhibitor.
**LOP_0000629**	-15,39	-36,57	LOP_0000629	Quinacrine dihydrochloride	Neurotransmission	Inhibitor	MAO	Monoamine oxidase (MAO) inhibitor; antimalarial
**LOP_0001454**	-15,75	-38,87	LOP_0001454	(S)-(+)-Camptothecin	Apoptosis	Inhibitor	TopoI	DNA topoisomerase I inhibitor
**LOP_0001276**	-15,93	-34,13	LOP_0001276	Thapsigargin	Intracellular Calcium	Releaser		Potent, cell-permeable, IP3-independent intracellular calcium releaser
**LOP_0000124**	-16,97	-64,59	LOP_0000124	SB-525334	Phosphorylation	Inhibitor	Alk5	A potent activin receptor-like kinase (ALK5)/ type I TGFß-receptor kinase inhibitor .
**LOP_0000432**	-17,65	-48,79	LOP_0000432	Forskolin	Cyclic Nucleotides	Activator	Adenylate cyclase	Activates adenylate cyclase; antihypertensive and vasodilator; isolated from Coleus forskohlii
**LOP_0000818**	-19,06	-29,78	LOP_0000818	Dihydroouabain	Ion Pump	Inhibitor	Na+/K+ Pump	Sodium-potassium pump inhibitor
