# Supplementary material for: Retinoic acid-induced 2 deficiency impairs genomic stability in breast cancer
Source: Breast Cancer Res. 2025 Jul 22;27:137. doi: 10.1186/s13058-025-02085-8 (PMC12285165; doi:10.1186/s13058-025-02085-8)
Supplement: Supplementary file 7 — Supplementary Material 7 [file 13058_2025_2085_MOESM7_ESM.pdf]

Supplementary Table S7: Results of SILAC-based immunoprecipitation and protein analysis by quantitative mass spectrometric proteomics

Accession Number	Protein name	Gene symbol	Mean SILAC ratio (RAI2/vector)	Unique peptides	Classification of protein/ protein interaction*	Evidence for PARP substrate
Q13363	C-terminal-binding protein 1	CTBP1	16.35	4	High confidence	(22, 23, 26)
Q9Y5P3	Retinoic acid-induced protein 2	RAI2	16.12	18	Bait protein	-
P05787	Keratin, type II cytoskeletal 8	KRT8	4.53	4	Low confidence	-
Q00610	Clathrin heavy chain 1	CLTC	2.80	27	High confidence	(24-26)
P38646	Stress-70 protein, mitochondrial	HSPA9	2.72	22	High confidence	(24, 26)
P0DMV8	Heat shock 70 kDa protein 1A	HSPA1A	2.66	20	High confidence	(24, 26)
Q9Y285	Phenylalanine--tRNA ligase alpha subunit	FARSA	2.52	2	Low confidence	-
Q9UPQ9	Trinucleotide repeat-containing gene 6B protein	TNRC6B	2.51	12	High confidence	-
Q92900	Regulator of nonsense transcripts 1	UPF1	2.21	7	High confidence	(24, 26)
Q9GZR7	ATP-dependent RNA helicase DDX24	DDX24	1.89	3	Low confidence	(26, 27)
Q9UMD9	Collagen alpha-1(XVII) chain;120 kDa linear IgA disease antigen;97 kDa linear IgA disease antigen	COL17A1	1.81	2	Low confidence	-
P04792	Heat shock protein beta-1	HSPB1	1.81	3	Low confidence	(24, 26, 28)
P63010	AP-2 complex subunit beta	AP2B1	1.77	3	High confidence	(24, 26)
P51114/P51116	Fragile X mental retardation syndrome-related protein 1	FXR1	1.76	3	Low confidence	(24, 26)
P11021	78 kDa glucose-regulated protein	HSPA5	1.72	23	Low confidence	(24, 26)
P09874	Poly [ADP-ribose] polymerase 1	PARP1	1.70	20	Low confidence	(24, 26, 28)
Q15233	Non-POU domain-containing octamer-binding protein	NONO	1.60	24	High confidence	(24, 26, 28)
O43809	Cleavage and polyadenylation specificity factor subunit 5	NUDT21	1.47	7	Low confidence	(24, 26)
Q02978	Mitochondrial 2-oxoglutarate/malate carrier protein	SLC25A11	1.37	2	Low confidence	(24, 26)
P23246	Splicing factor, proline- and glutamine-rich	SFPQ	1.36	14	Low confidence	(24, 26, 28)
P11142	Heat shock cognate 71 kDa protein	HSPA8	1.35	25	High confidence	(24, 26, 28)

High confidence: significant in 2 or 3 biological replicates; low confidence: significant in one biological replicate
