# Supplementary material for: Retinoic acid-induced 2 deficiency impairs genomic stability in breast cancer
Source: Breast Cancer Res. 2025 Jul 22;27:137. doi: 10.1186/s13058-025-02085-8 (PMC12285165; doi:10.1186/s13058-025-02085-8)
Supplement: Supplementary file 8 — Supplementary Material 8 [file 13058_2025_2085_MOESM8_ESM.pdf]

Supplementary Figure S1: Correlation of *RAI2* gene expression with genes of CIN70 signature in the METABRIC data set.

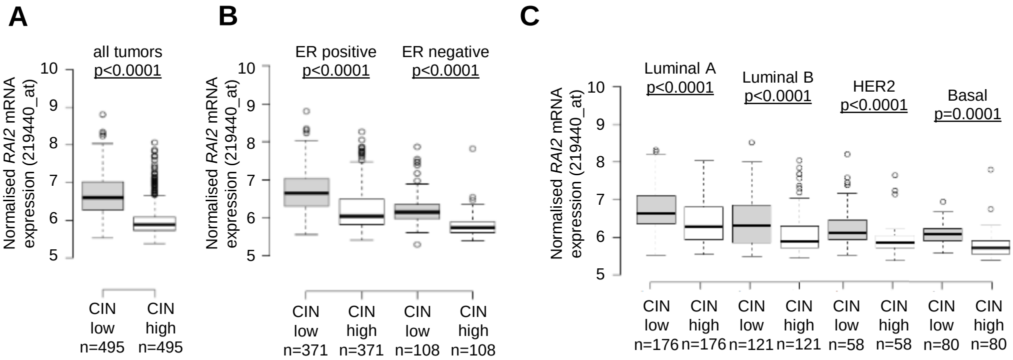

D

	Estimate	LL	UL	p-value
(Intercept)	2.386	2.239	2.533	2,79E-140
CIN70	-0.0009685	-0.001244	-0.0006933	1,02E-08
ER_IHC_status pos	0.000212	-0.02626	0.02669	0.9875
Pam50SubtypeBasal	-0.05196	-0.08561	-0.01831	0.002516
Pam50SubtypeHer2	-0.0637	-0.09389	-0.0335	3,84E-02
Pam50SubtypeLumB	-0.01103	-0.02798	0.00593	0.2022
Pam50SubtypeNormal	-0.04271	-0.06701	-0.01841	0.00059
P53_mutation_statusMUT	-0.007415	-0.0251	0.01027	0.4107

RAI2 gene expression is shown in all samples of the data set (A) or after subgrouping according to estrogen receptor (ER) α-status (B) or according to the molecular subtypes of breast cancer (C) (Luminal A and B, HER2, basal). For calculation of p-values Student's t-test was applied. D) Results of the multivariable linear regression analysis between the CIN70 score and low RAI2 gene expression in the METABRIC data set.
