# Supplementary material for: Retinoic acid-induced 2 deficiency impairs genomic stability in breast cancer
Source: Breast Cancer Res. 2025 Jul 22;27:137. doi: 10.1186/s13058-025-02085-8 (PMC12285165; doi:10.1186/s13058-025-02085-8)

Supplementary Figure S2: Correlations of gene expression values of *RAI2*, genes of the CIN70 and known DNA repair genes

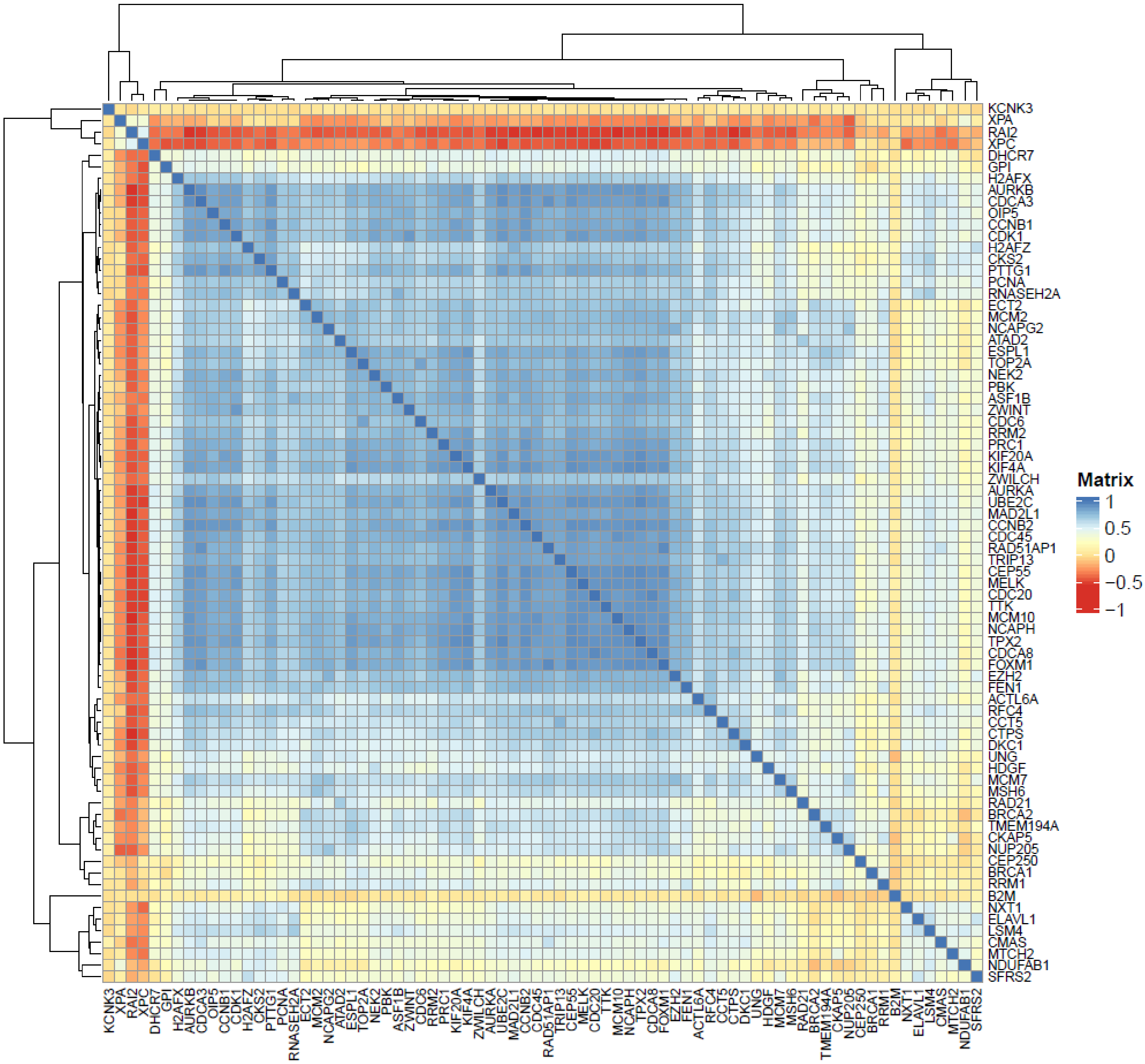

Supplement: Supplementary file 9 — Supplementary Material 9 [file 13058_2025_2085_MOESM9_ESM.pdf]
