# Supplementary material for: Retinoic acid-induced 2 deficiency impairs genomic stability in breast cancer
Source: Breast Cancer Res. 2025 Jul 22;27:137. doi: 10.1186/s13058-025-02085-8 (PMC12285165; doi:10.1186/s13058-025-02085-8)
Supplement: Supplementary file 10 — Supplementary Material 10 [file 13058_2025_2085_MOESM10_ESM.pdf]

Supplementary Figure S3: Correlations of gene expression values with CIN70 and wGII scores. Genes whose gene products are involved in DNA repair and negative controls were selected

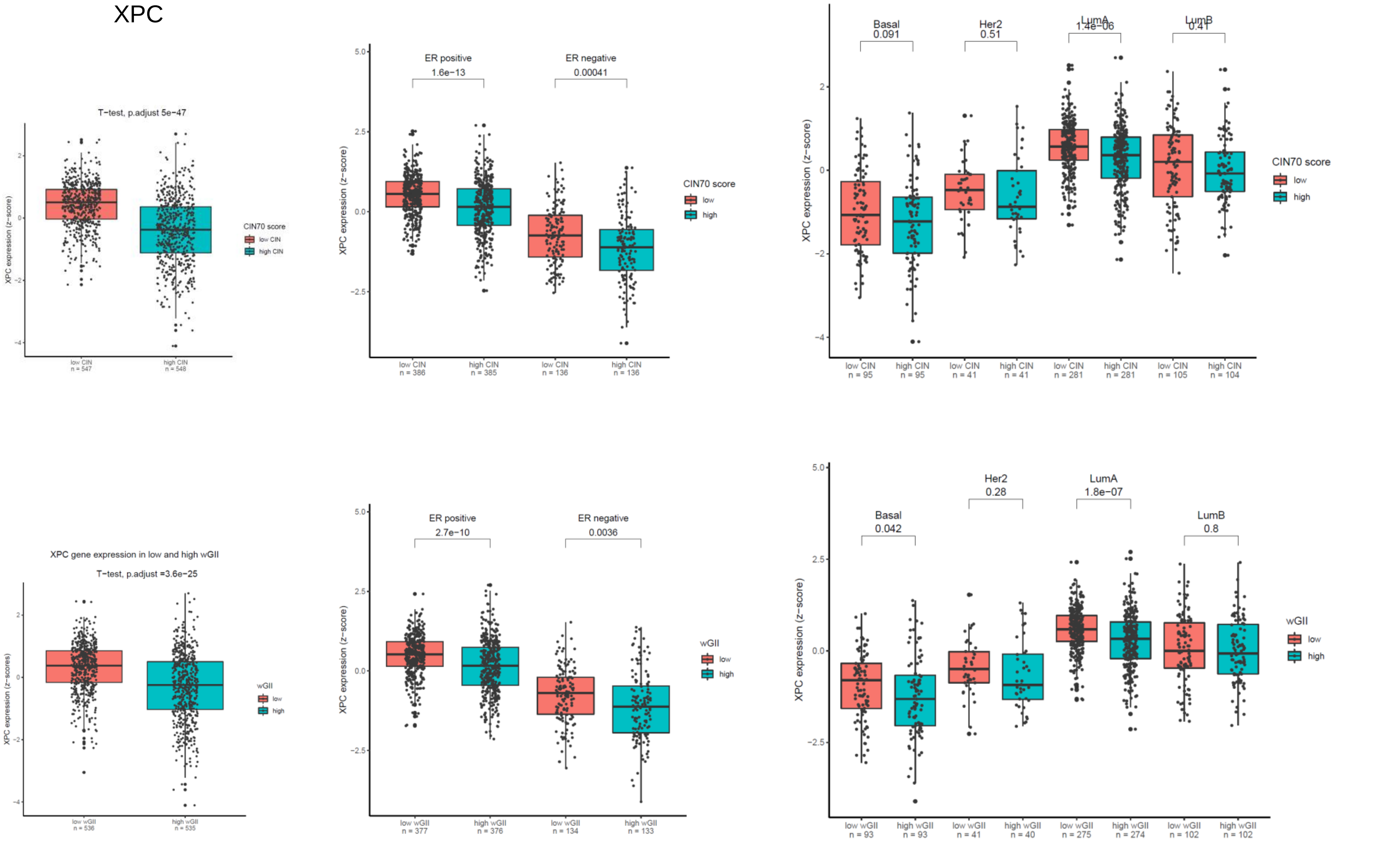

XPA

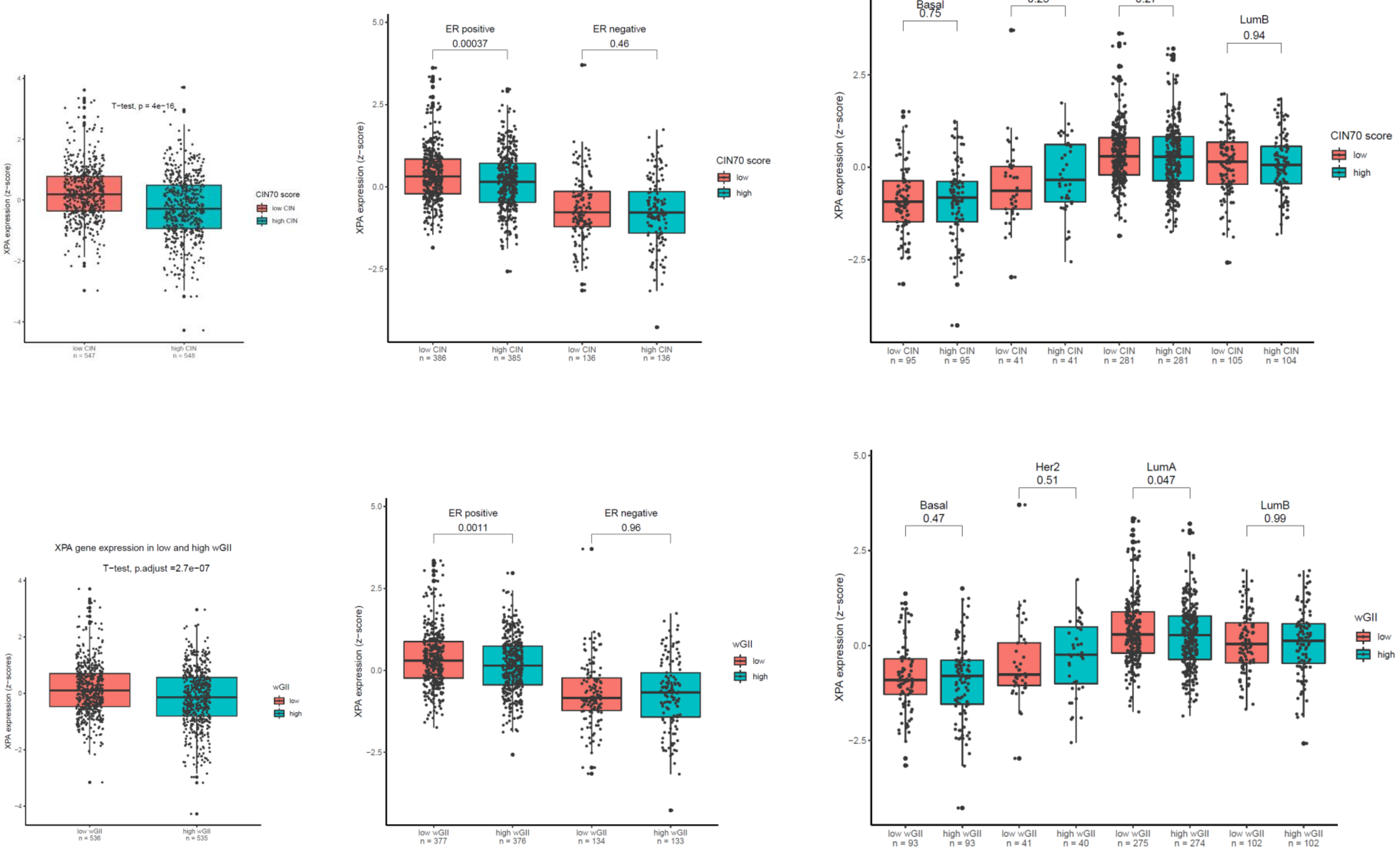

T−test, p = 4e−16
XPA expression (z−score)
CIN70 score
low CIN
high CIN
low CIN n = 547
high CIN n = 548
ER positive
0.00037
ER negative
0.46
XPA expression (z−score)
CIN70 score
low
high
low CIN n = 386
high CIN n = 385
low CIN n = 136
high CIN n = 136
Basal
0.75
Her2
0.25
LumA
0.27
LumB
0.94
XPA expression (z−score)
CIN70 score
low
high
low CIN n = 95
high CIN n = 95
low CIN n = 41
high CIN n = 41
low CIN n = 281
high CIN n = 281
low CIN n = 105
high CIN n = 104
XPA gene expression in low and high wGII
T−test, p.adjust =2.7e−07
XPA expression (z−scores)
wGII
low
high
low wGII n = 536
high wGII n = 535
ER positive
0.0011
ER negative
0.96
XPA expression (z−score)
wGII
low
high
low wGII n = 377
high wGII n = 376
low wGII n = 134
high wGII n = 133
Basal
0.47
Her2
0.51
LumA
0.047
LumB
0.99
XPA expression (z−score)
wGII
low
high
low wGII n = 93
high wGII n = 93
low wGII n = 41
high wGII n = 40
low wGII n = 275
high wGII n = 274
low wGII n = 102
high wGII n = 102

BRCA1

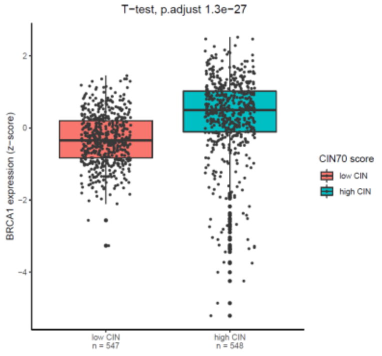
T−test, p.adjust 1.3e−27
BRCA1 expression (z−score)
low CIN
n = 547
high CIN
n = 548
CIN70 score
low CIN
high CIN

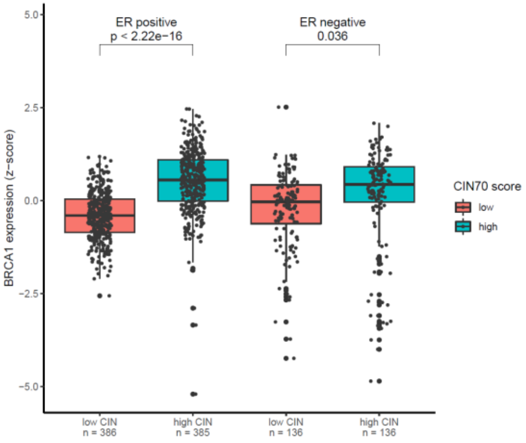
ER positive
p < 2.22e−16
ER negative
0.036
BRCA1 expression (z−score)
low CIN
n = 386
high CIN
n = 385
low CIN
n = 136
high CIN
n = 136
CIN70 score
low
high

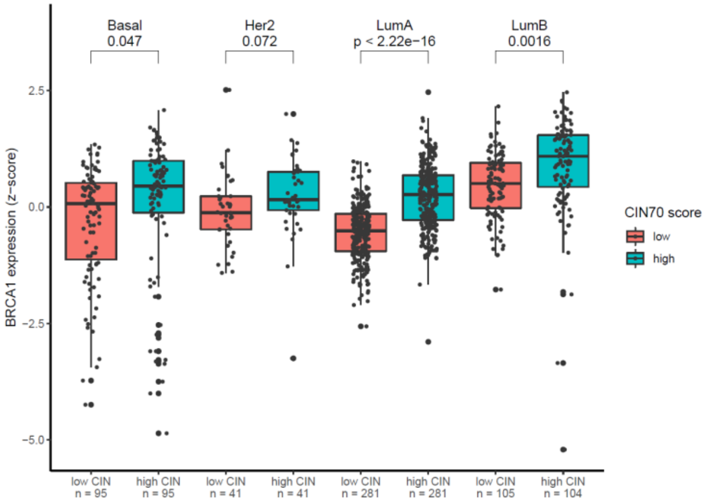
Basal
0.047
Her2
0.072
LumA
p < 2.22e−16
LumB
0.0016
BRCA1 expression (z−score)
low CIN
n = 95
high CIN
n = 95
low CIN
n = 41
high CIN
n = 41
low CIN
n = 281
high CIN
n = 281
low CIN
n = 105
high CIN
n = 104
CIN70 score
low
high

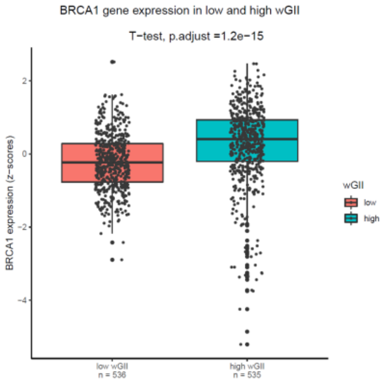
BRCA1 gene expression in low and high wGII
T−test, p.adjust =1.2e−15
BRCA1 expression (z−scores)
low wGII
n = 536
high wGII
n = 535
wGII
low
high

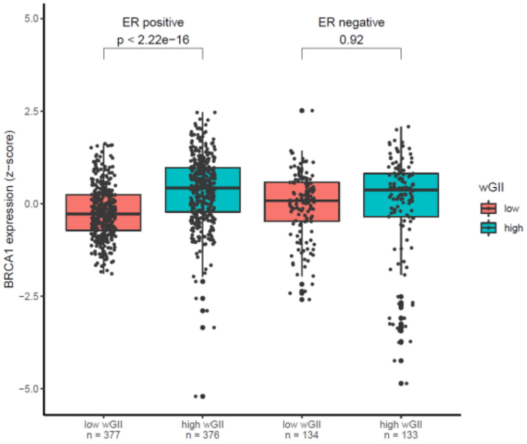
ER positive
p < 2.22e−16
ER negative
0.92
BRCA1 expression (z−score)
low wGII
n = 377
high wGII
n = 376
low wGII
n = 134
high wGII
n = 133
wGII
low
high

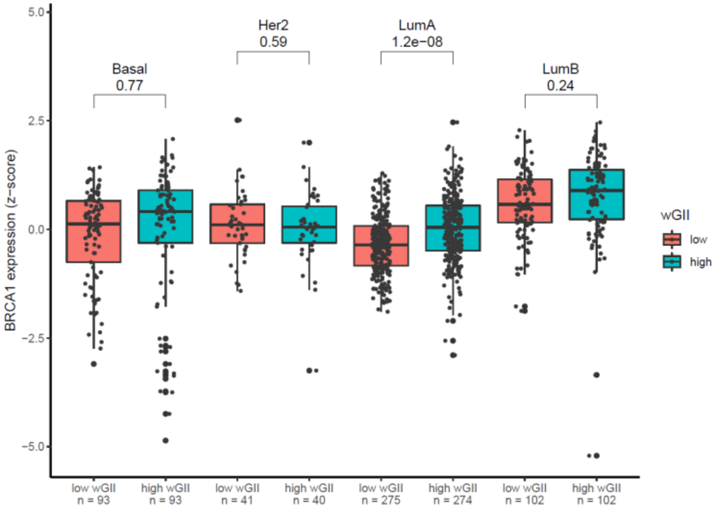
Basal
0.77
Her2
0.59
LumA
1.2e−08
LumB
0.24
BRCA1 expression (z−score)
low wGII
n = 93
high wGII
n = 93
low wGII
n = 41
high wGII
n = 40
low wGII
n = 275
high wGII
n = 274
low wGII
n = 102
high wGII
n = 102
wGII
low
high

BRCA2

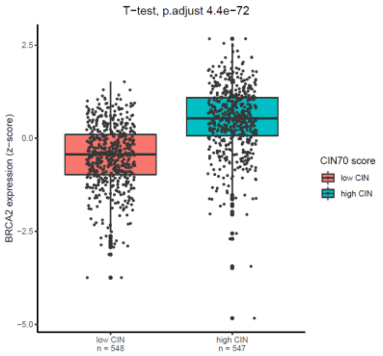

T−test, p.adjust 4.4e−72
BRCA2 expression (z−score)
CIN70 score
low CIN
high CIN
low CIN
n = 548
high CIN
n = 547

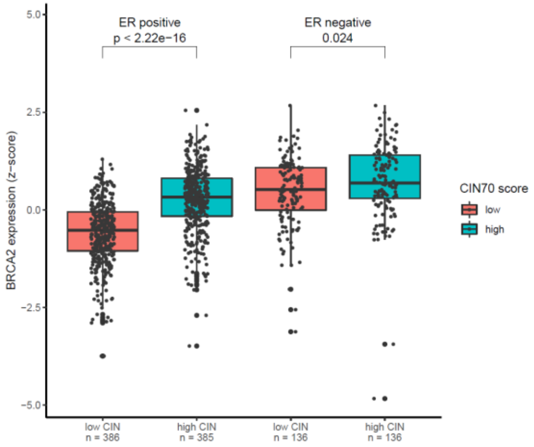

ER positive
p < 2.22e−16
ER negative
0.024
BRCA2 expression (z−score)
CIN70 score
low
high
low CIN
n = 386
high CIN
n = 385
low CIN
n = 136
high CIN
n = 136

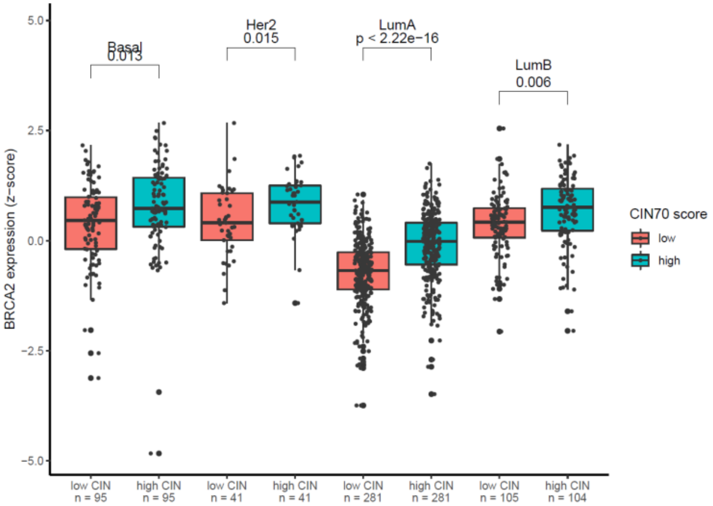

Basal
0.013
Her2
0.015
LumA
p < 2.22e−16
LumB
0.006
BRCA2 expression (z−score)
CIN70 score
low
high
low CIN
n = 95
high CIN
n = 95
low CIN
n = 41
high CIN
n = 41
low CIN
n = 281
high CIN
n = 281
low CIN
n = 105
high CIN
n = 104

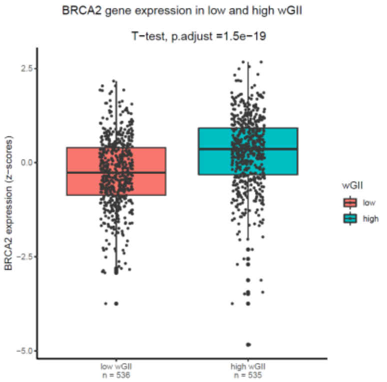

BRCA2 gene expression in low and high wGII
T−test, p.adjust =1.5e−19
BRCA2 expression (z−scores)
wGII
low
high
low wGII
n = 536
high wGII
n = 535

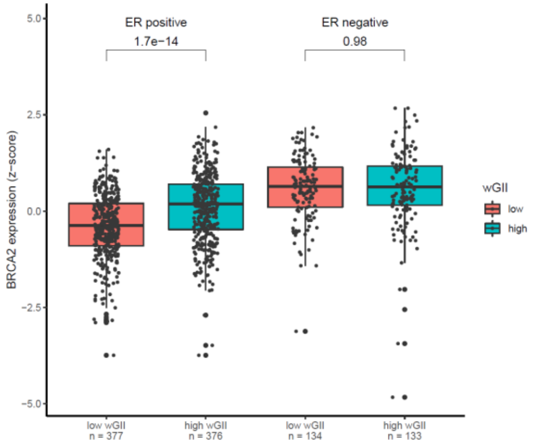

ER positive
1.7e−14
ER negative
0.98
BRCA2 expression (z−score)
wGII
low
high
low wGII
n = 377
high wGII
n = 376
low wGII
n = 134
high wGII
n = 133

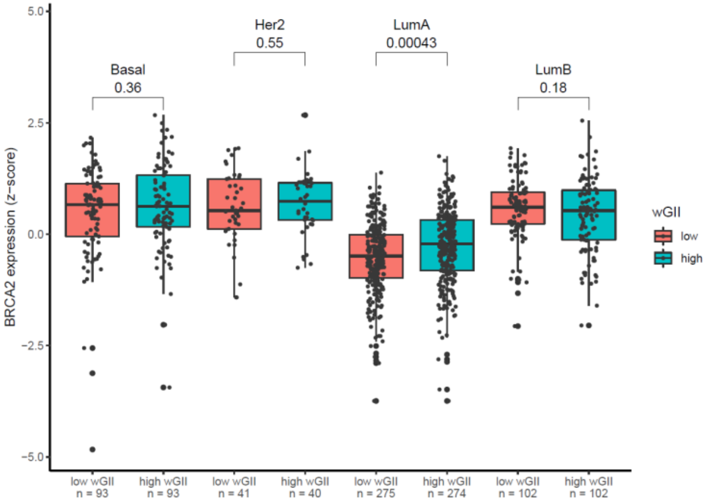

Basal
0.36
Her2
0.55
LumA
0.00043
LumB
0.18
BRCA2 expression (z−score)
wGII
low
high
low wGII
n = 93
high wGII
n = 93
low wGII
n = 41
high wGII
n = 40
low wGII
n = 275
high wGII
n = 274
low wGII
n = 102
high wGII
n = 102

B2M

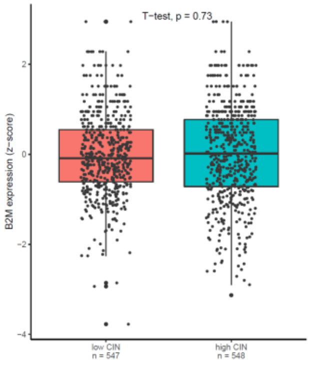

T−test, p = 0.73
B2M expression (z−score)
low CIN
n = 547
high CIN
n = 548
CIN70 score
low CIN
high CIN

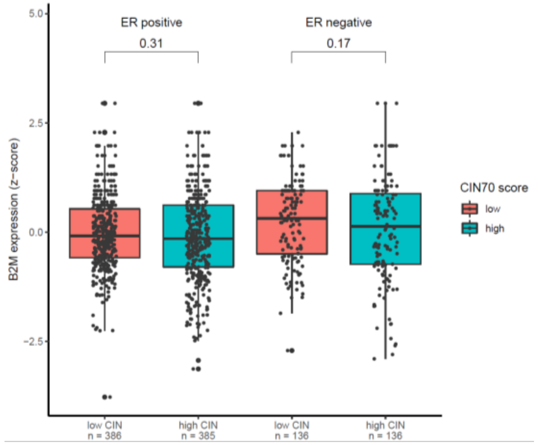

ER positive
0.31
ER negative
0.17
B2M expression (z−score)
low CIN
n = 386
high CIN
n = 385
low CIN
n = 136
high CIN
n = 136
CIN70 score
low
high

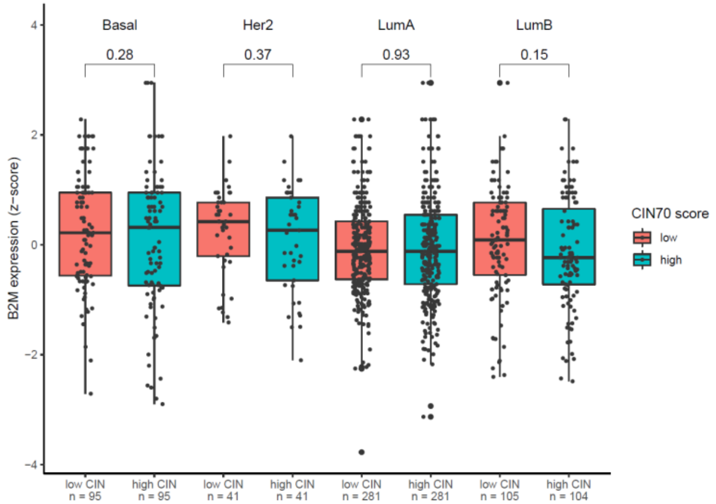

Basal
0.28
Her2
0.37
LumA
0.93
LumB
0.15
B2M expression (z−score)
low CIN
n = 95
high CIN
n = 95
low CIN
n = 41
high CIN
n = 41
low CIN
n = 281
high CIN
n = 281
low CIN
n = 105
high CIN
n = 104
CIN70 score
low
high

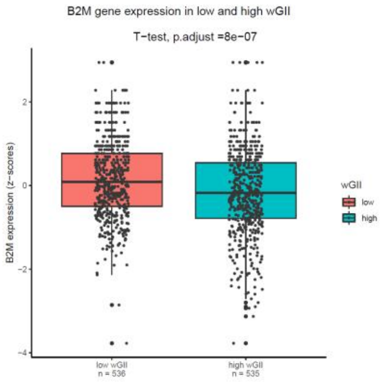

B2M gene expression in low and high wGII
T−test, p.adjust =8e−07
B2M expression (z−scores)
low wGII
n = 536
high wGII
n = 535
wGII
low
high

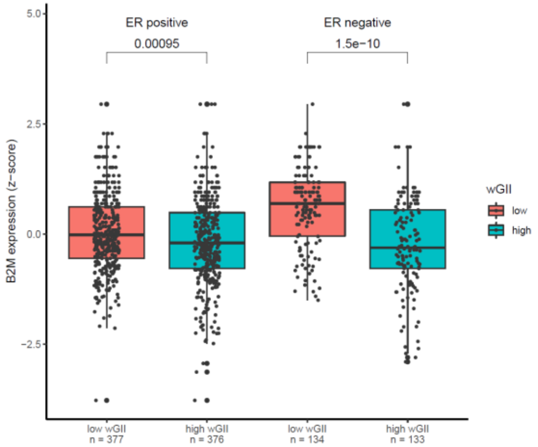

ER positive
0.00095
ER negative
1.5e−10
B2M expression (z−score)
low wGII
n = 377
high wGII
n = 376
low wGII
n = 134
high wGII
n = 133
wGII
low
high

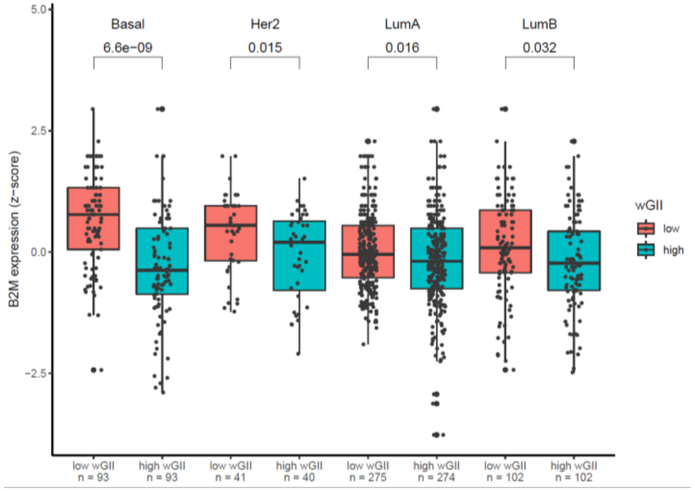

Basal
6.6e−09
Her2
0.015
LumA
0.016
LumB
0.032
B2M expression (z−score)
low wGII
n = 93
high wGII
n = 93
low wGII
n = 41
high wGII
n = 40
low wGII
n = 275
high wGII
n = 274
low wGII
n = 102
high wGII
n = 102
wGII
low
high

KCNK3

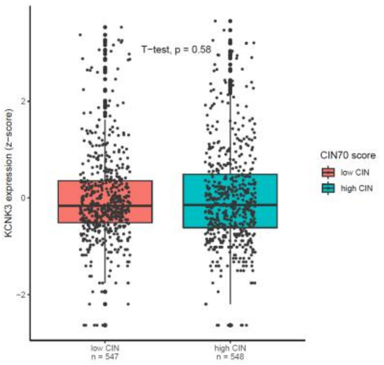

T−test, p = 0.58
KCNK3 expression (z−scores)
2
0
−2
low CIN
n = 547
high CIN
n = 548
CIN70 score
low CIN
high CIN

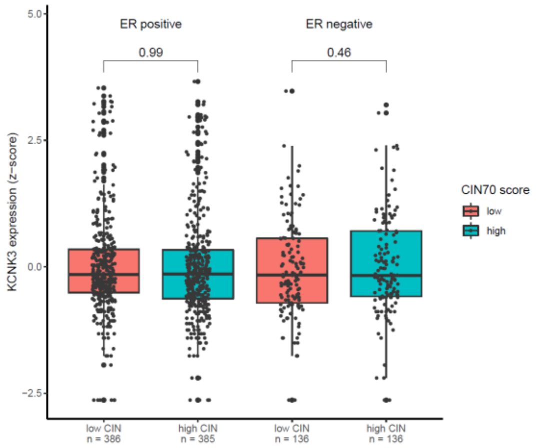

5.0
2.5
0.0
−2.5
KCNK3 expression (z−score)
ER positive
ER negative
0.99
0.46
low CIN
n = 386
high CIN
n = 385
low CIN
n = 136
high CIN
n = 136
CIN70 score
low
high

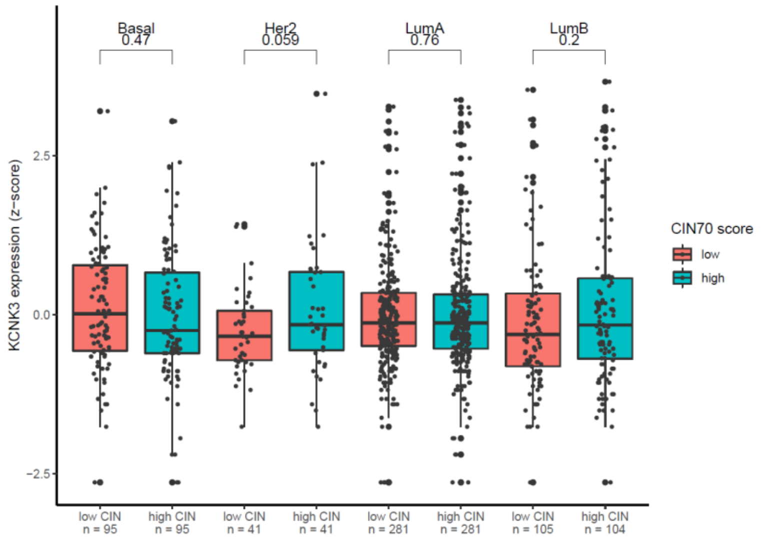

Basal
0.47
Her2
0.059
LumA
0.76
LumB
0.2
2.5
0.0
−2.5
KCNK3 expression (z−score)
low CIN
n = 95
high CIN
n = 95
low CIN
n = 41
high CIN
n = 41
low CIN
n = 281
high CIN
n = 281
low CIN
n = 105
high CIN
n = 104
CIN70 score
low
high

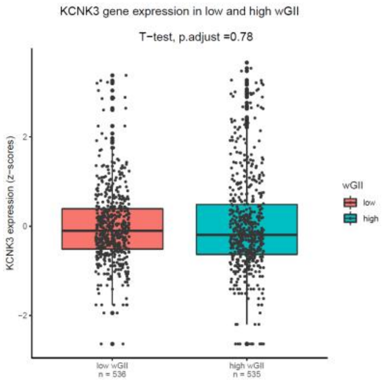

KCNK3 gene expression in low and high wGII
T−test, p.adjust =0.78
KCNK3 expression (z−scores)
2
0
−2
low wGII
n = 536
high wGII
n = 535
wGII
low
high

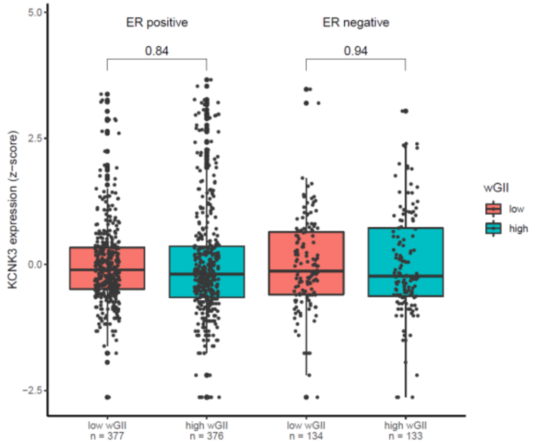

5.0
2.5
0.0
−2.5
KCNK3 expression (z−score)
ER positive
ER negative
0.84
0.94
low wGII
n = 377
high wGII
n = 376
low wGII
n = 134
high wGII
n = 133
wGII
low
high

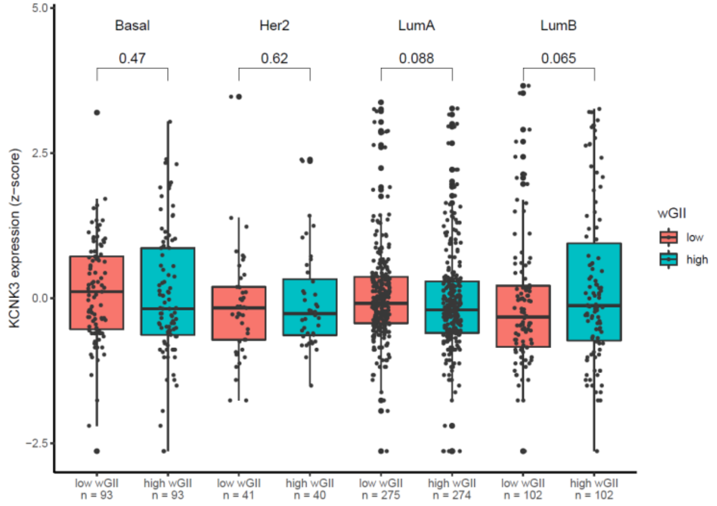

5.0
2.5
0.0
−2.5
KCNK3 expression (z−score)
Basal
Her2
LumA
LumB
0.47
0.62
0.088
0.065
low wGII
n = 93
high wGII
n = 93
low wGII
n = 41
high wGII
n = 40
low wGII
n = 275
high wGII
n = 274
low wGII
n = 102
high wGII
n = 102
wGII
low
high
