# Supplementary material for: Retinoic acid-induced 2 deficiency impairs genomic stability in breast cancer
Source: Breast Cancer Res. 2025 Jul 22;27:137. doi: 10.1186/s13058-025-02085-8 (PMC12285165; doi:10.1186/s13058-025-02085-8)

Supplementary Figure 4: Kaplan-Meier estimation of overall survival in breast cancer patients from the METABRIC dataset, stratified by median, CIN score, and RAI2 gene expression in ER-positive and negative, and molecular subtypes.

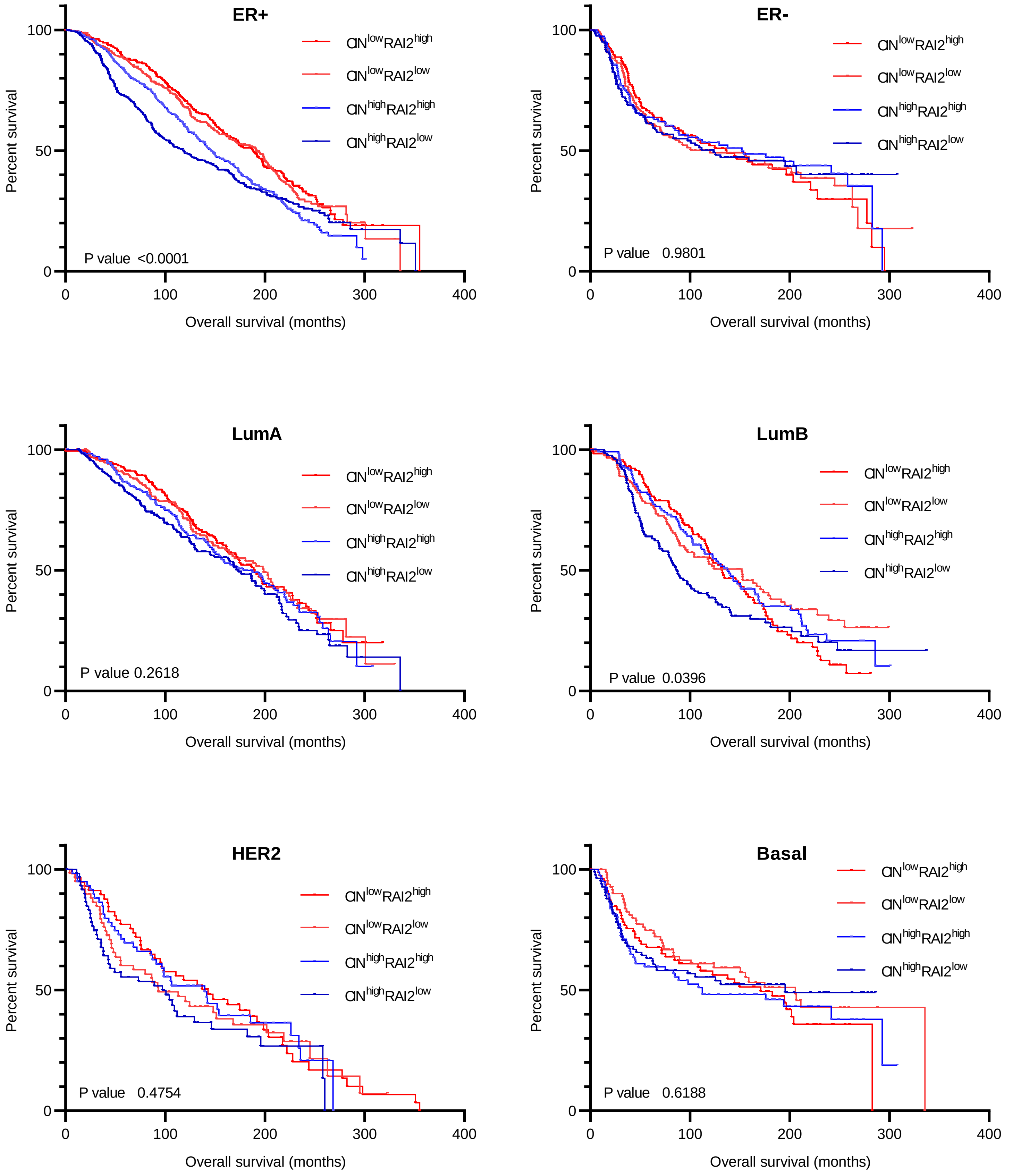

Supplement: Supplementary file 11 — Supplementary Material 11 [file 13058_2025_2085_MOESM11_ESM.pdf]
