# Supplementary material for: Retinoic acid-induced 2 deficiency impairs genomic stability in breast cancer
Source: Breast Cancer Res. 2025 Jul 22;27:137. doi: 10.1186/s13058-025-02085-8 (PMC12285165; doi:10.1186/s13058-025-02085-8)

Supplementary Figure S6: Assessment of chromosome segregation errors in RAI2-depleted breast cancer cell lines. **A)** Frequencies of lagging chromosomes with centromere during metaphase **B)** Frequencies of lagging chromosomes with centromere during anaphase. **C)** Frequencies of chromosome bridges during anaphase. Values shown are the mean frequency of three independent experiments. * Indicates a p-value below the significance threshold of 0.05 calculated by a two-tailed t-test. Ns: no significant difference.

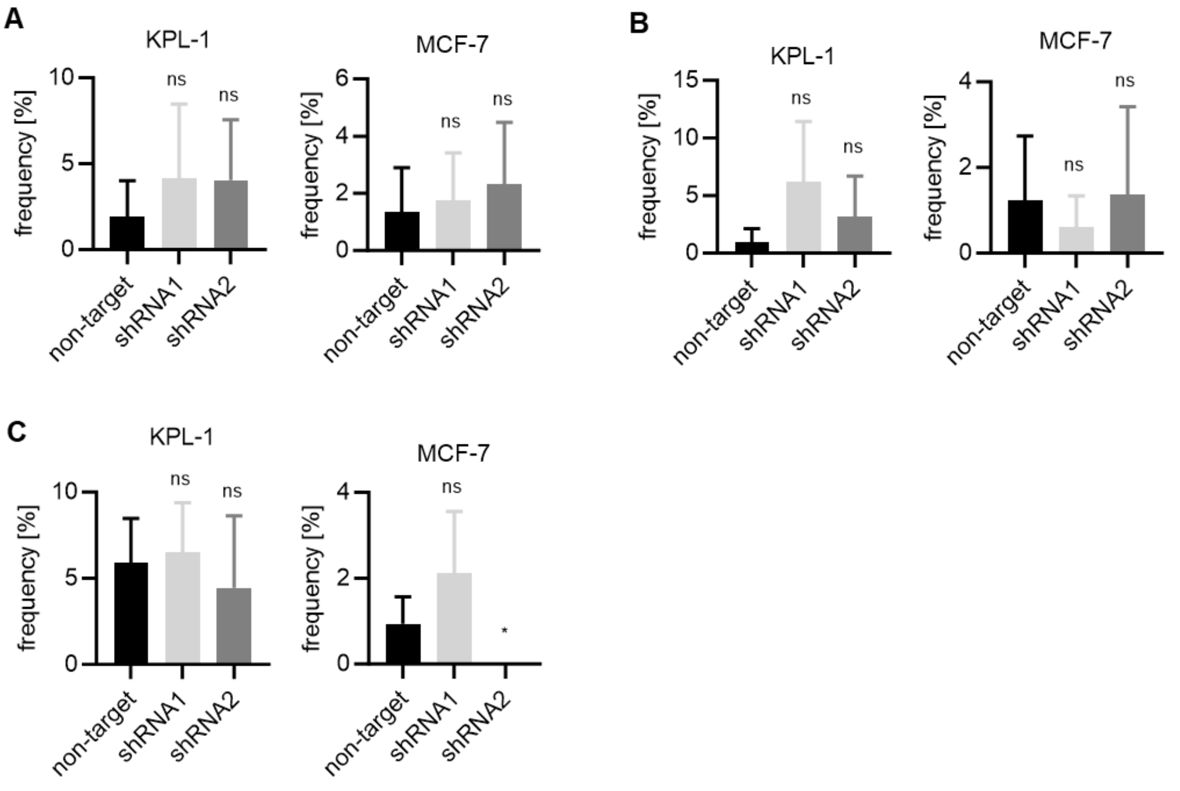

Supplement: Supplementary file 13 — Supplementary Material 13 [file 13058_2025_2085_MOESM13_ESM.pdf]
