# Supplementary material for: Retinoic acid-induced 2 deficiency impairs genomic stability in breast cancer
Source: Breast Cancer Res. 2025 Jul 22;27:137. doi: 10.1186/s13058-025-02085-8 (PMC12285165; doi:10.1186/s13058-025-02085-8)

Supplementary Figure S7: Validation of binding partners of RAI2 found in SILAC analysis by combined immunoprecipitation (IP) and Western blot analysis

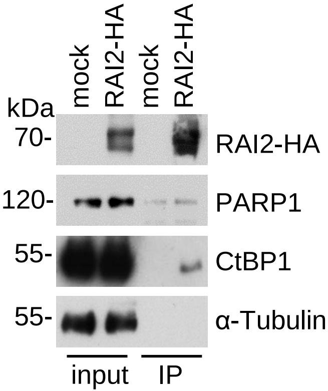

Supplement: Supplementary file 14 — Supplementary Material 14 [file 13058_2025_2085_MOESM14_ESM.pdf]
