# Supplementary material for: Retinoic acid-induced 2 deficiency impairs genomic stability in breast cancer
Source: Breast Cancer Res. 2025 Jul 22;27:137. doi: 10.1186/s13058-025-02085-8 (PMC12285165; doi:10.1186/s13058-025-02085-8)

Supplementary Figure S9: Colocalization analysis of RAI2 and CtBP1 in DMSO and CPT treated KPL-1 and MCF-7 cells

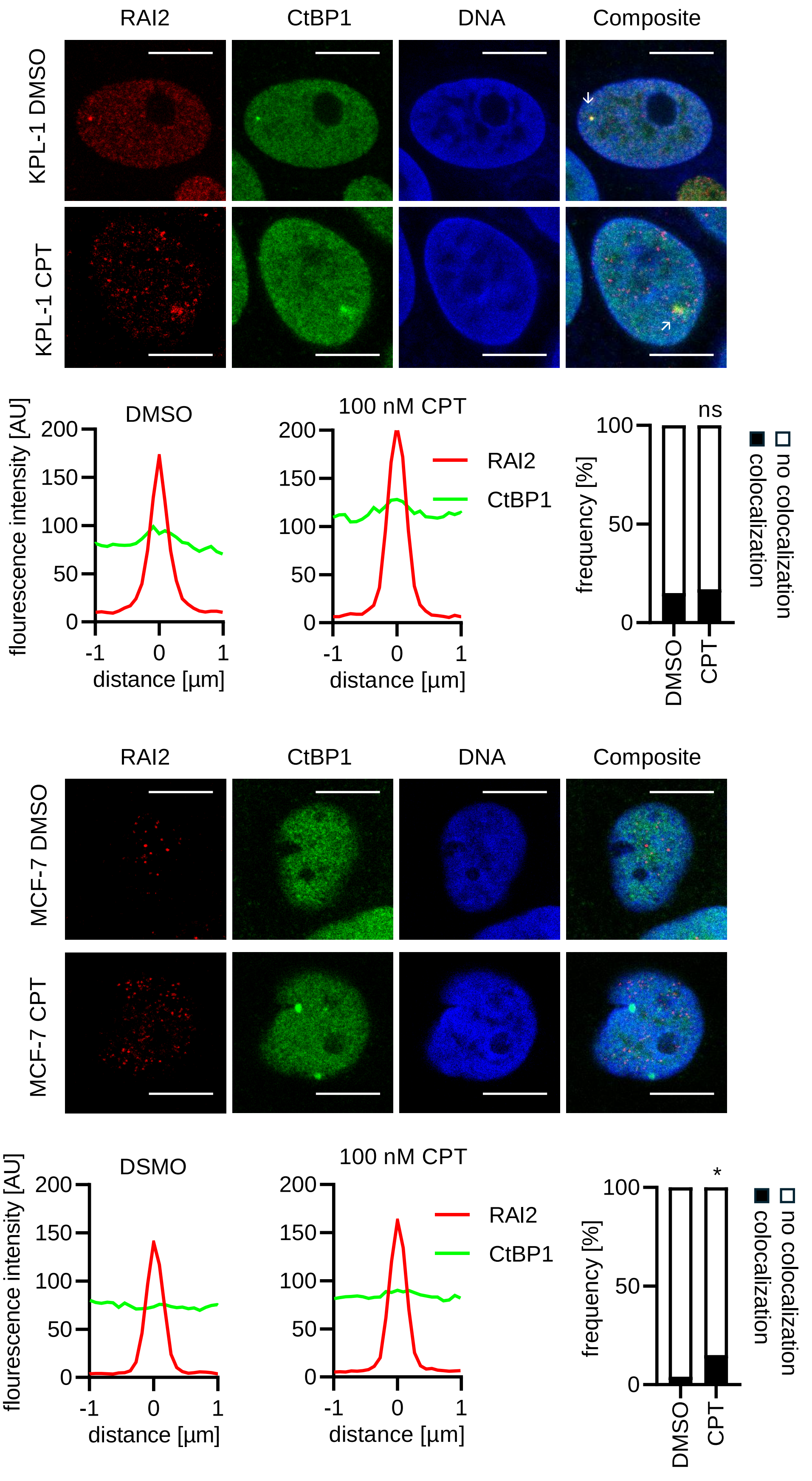

Supplement: Supplementary file 16 — Supplementary Material 16 [file 13058_2025_2085_MOESM16_ESM.pdf]
