# Supplementary material for: Retinoic acid-induced 2 deficiency impairs genomic stability in breast cancer
Source: Breast Cancer Res. 2025 Jul 22;27:137. doi: 10.1186/s13058-025-02085-8 (PMC12285165; doi:10.1186/s13058-025-02085-8)

Supplementary Figure S11: Analysis of phenotypic features after reconstitution of RAI2 at physiological level in KPL-1 cells. **(A)** Western blot analysis of cell G2/M associated proteins, **(B)** proliferation analysis, **(C)** detection of double stranded DNA breaks

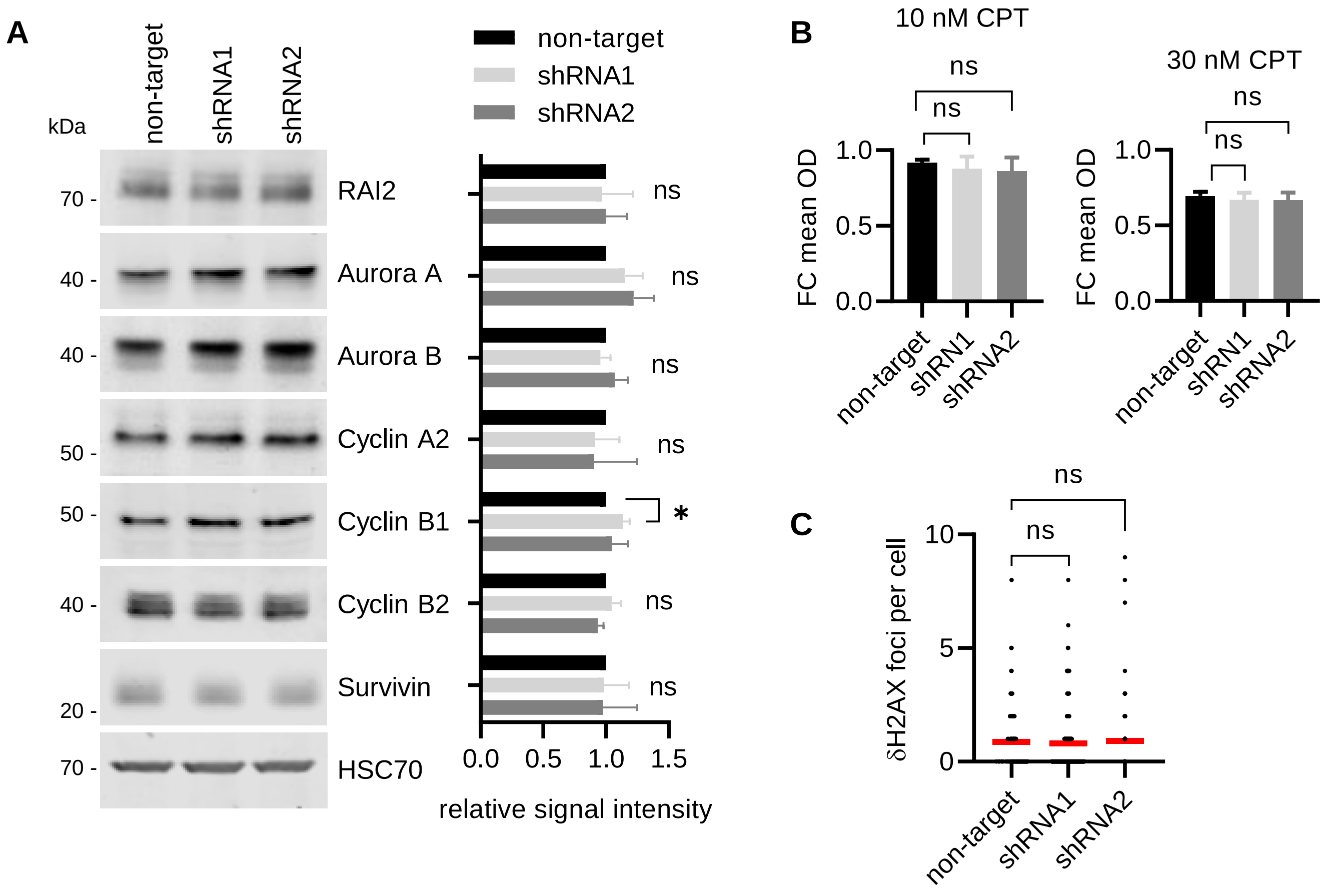

Supplement: Supplementary file 18 — Supplementary Material 18 [file 13058_2025_2085_MOESM18_ESM.pdf]
