# Supplementary material for: Retinoic acid-induced 2 deficiency impairs genomic stability in breast cancer
Source: Breast Cancer Res. 2025 Jul 22;27:137. doi: 10.1186/s13058-025-02085-8 (PMC12285165; doi:10.1186/s13058-025-02085-8)

# Western blot analysis of cell cycle-related proteins in RAI2-depleted KPL-1 cells (Figure 2C)

To enable multiplex analysis, some membranes are cut after electro-transfer or membranes were reprobed.
Thus, different blots can share the same loading control.

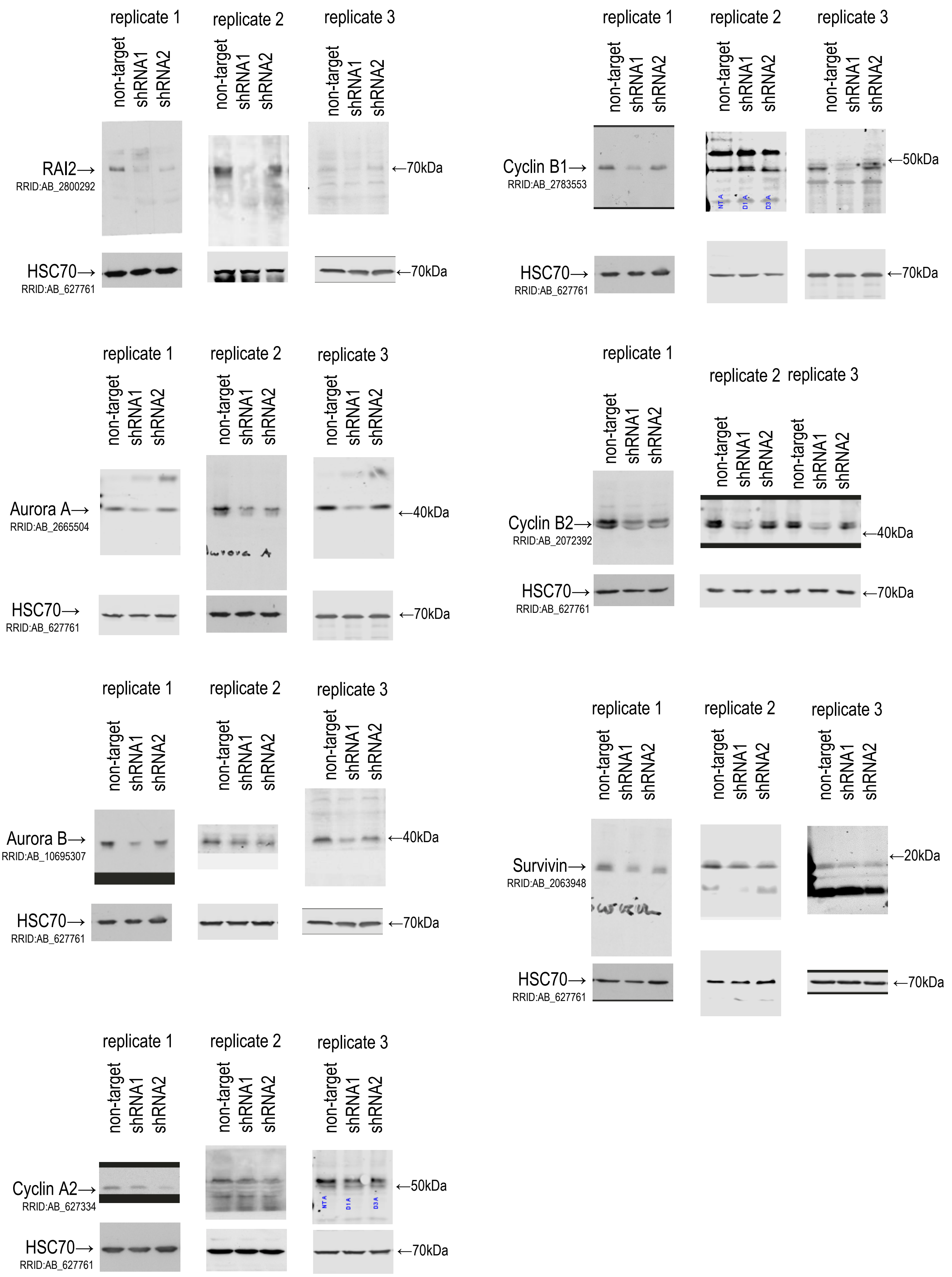

# Western blot analysis of cell cycle-related proteins in RAI2-depleted CAMA-1 cells (Figure 2C)

To enable multiplex analysis, some membranes are cut after electro-transfer or membranes were reprobed.
Thus, different blots can share the same loading control.

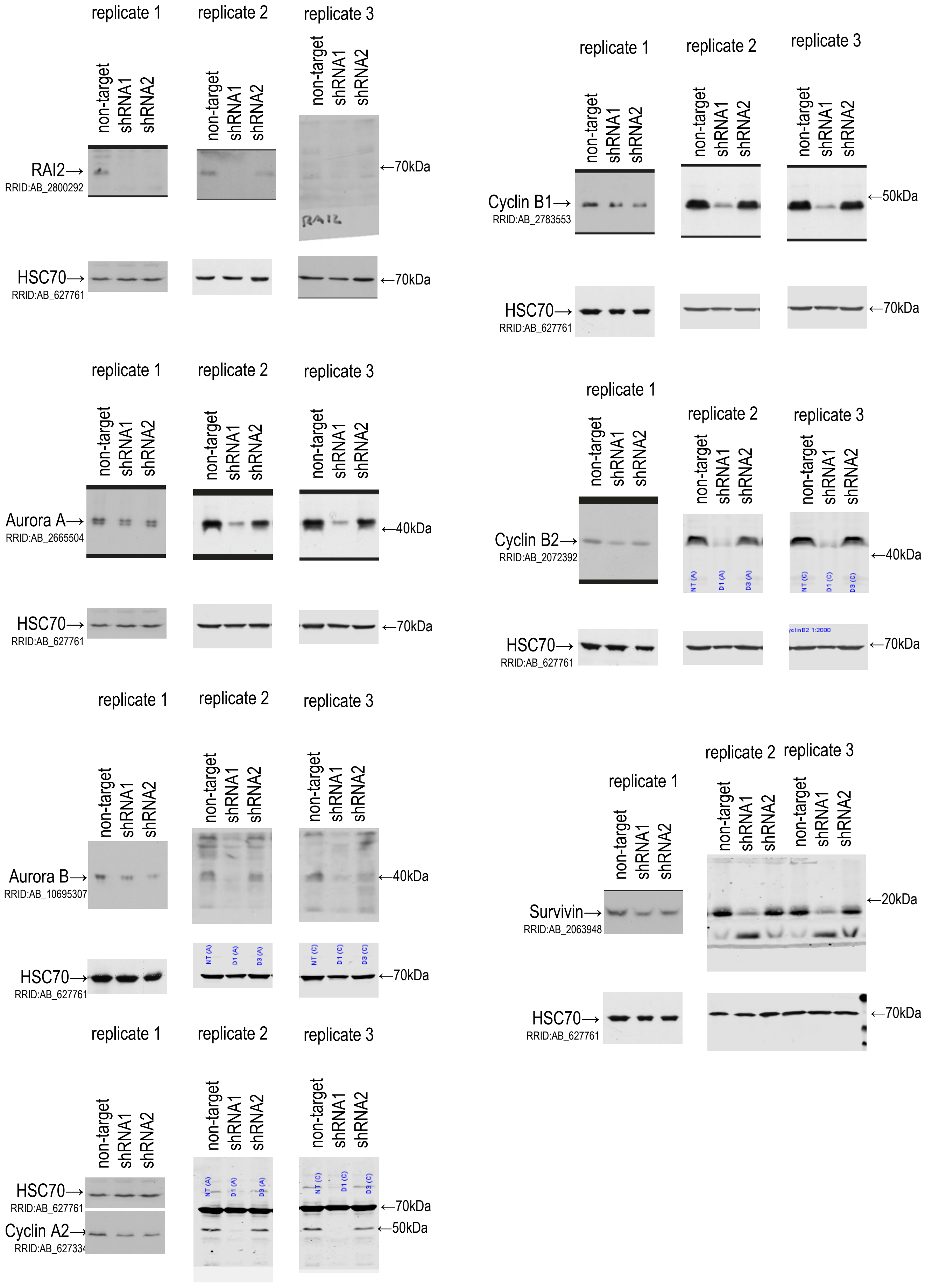

Western blot analysis of cell cycle-related proteins in RAI2-depleted MCF-7 cells (Figure 2C)

To enable multiplex analysis, some membranes are cut after electro-transfer or membranes were reprobed. Thus, different blots can share the same loading control

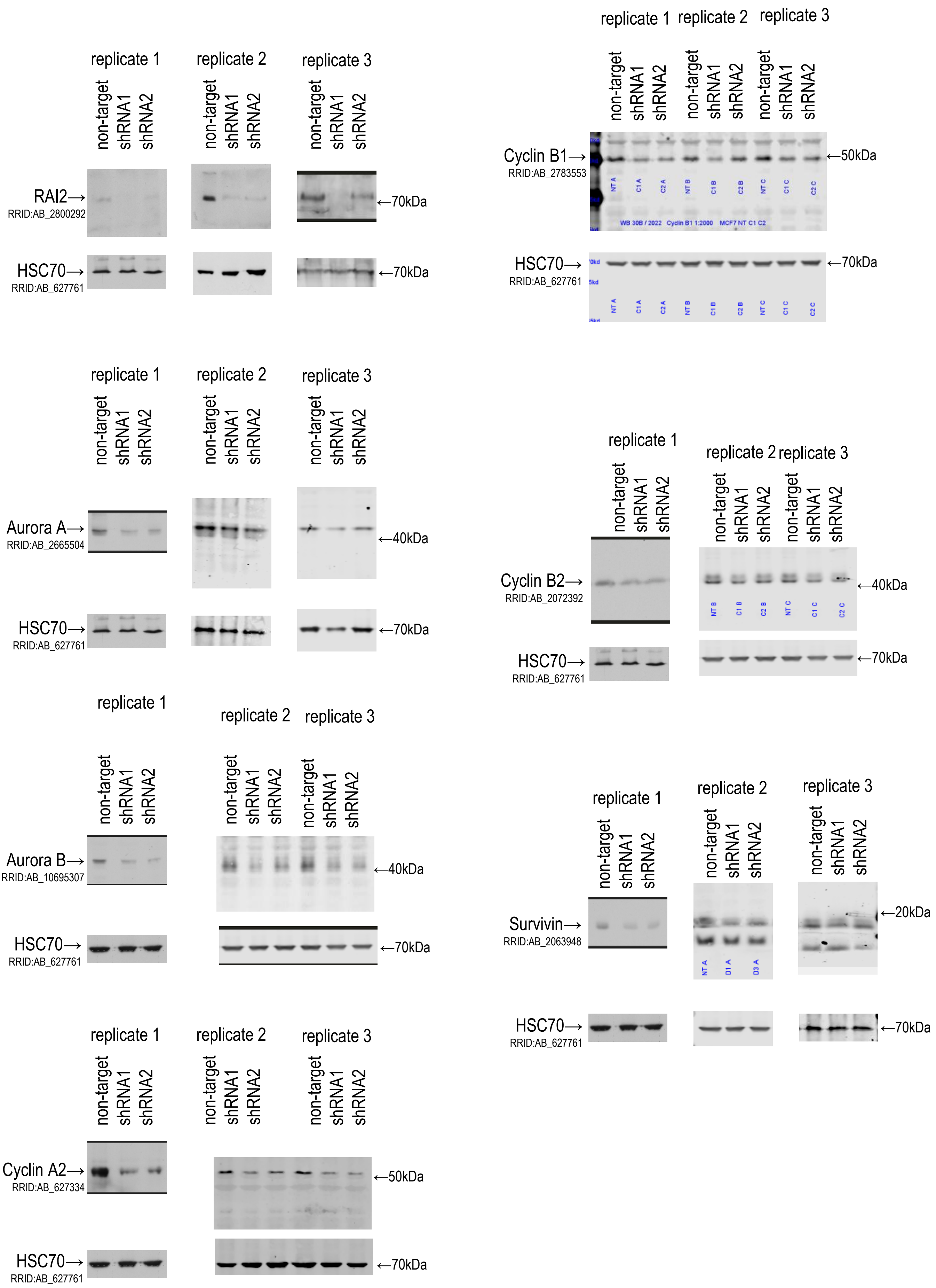

Validation of RAI2 knockdown in KPL-1 cells with constitutive overexpression of H2B-GFP (Figure 3D)

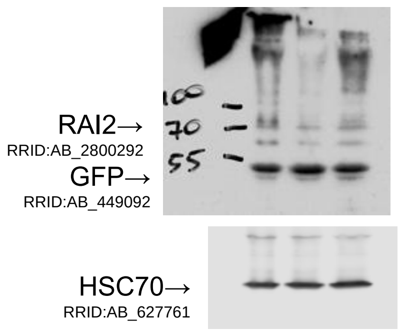


Validation of RAI2 overexpression in transiently transfected 293T cells (Figure 6C)

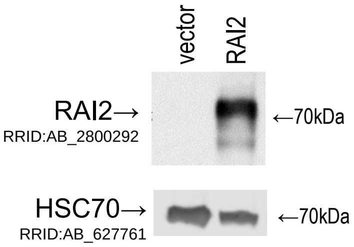


Western blot analysis of RAI2 and δH2AX induction in CPT treated KPL-1 cells (Figure 5B)

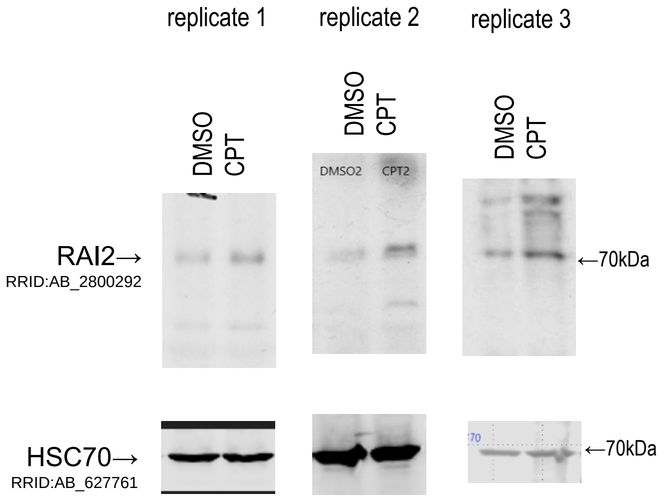


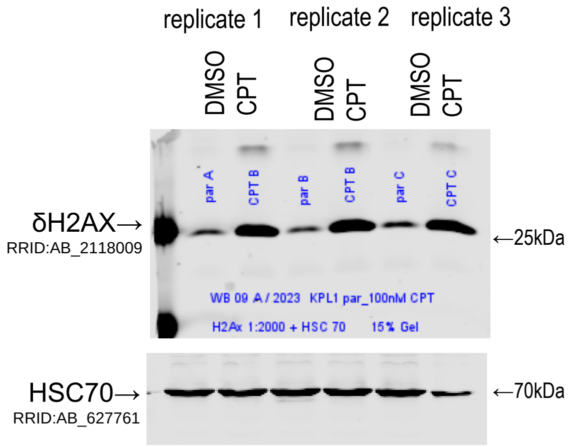


Western blot analysis of RAI2 and δH2AX induction in CPT treated MCF-7 cells (Figure 5B)

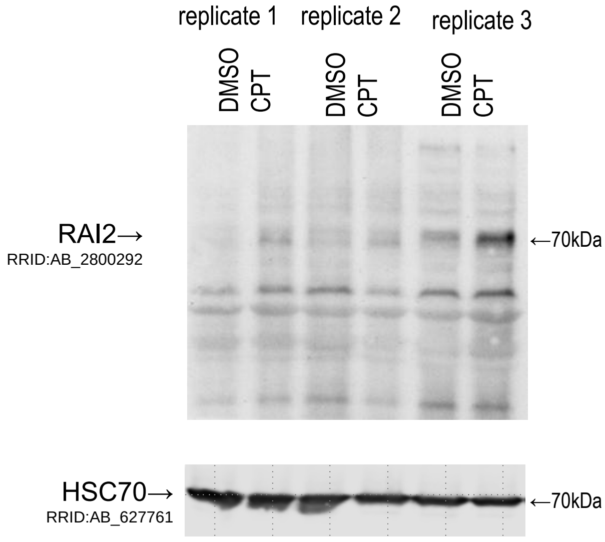


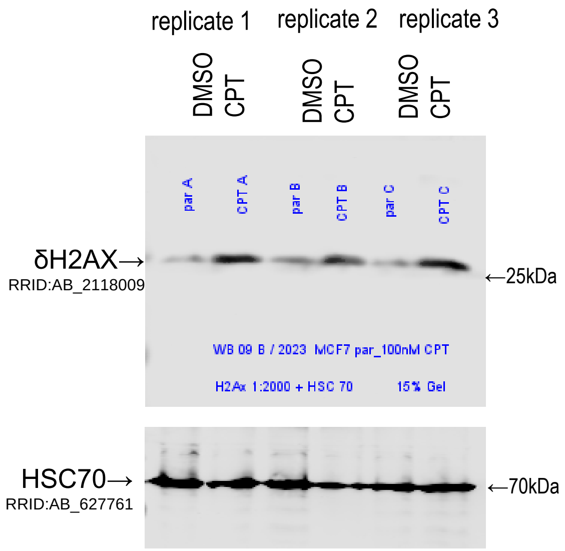

Western blot analysis of cell cycle-related proteins in KPL-1 cells with recovered RAI2 protein (Supplementary Figure S11)

To enable multiplex analysis, some membranes are cut after electro-transfer or membranes were reprobed. Thus, different blots can share the same loading control.

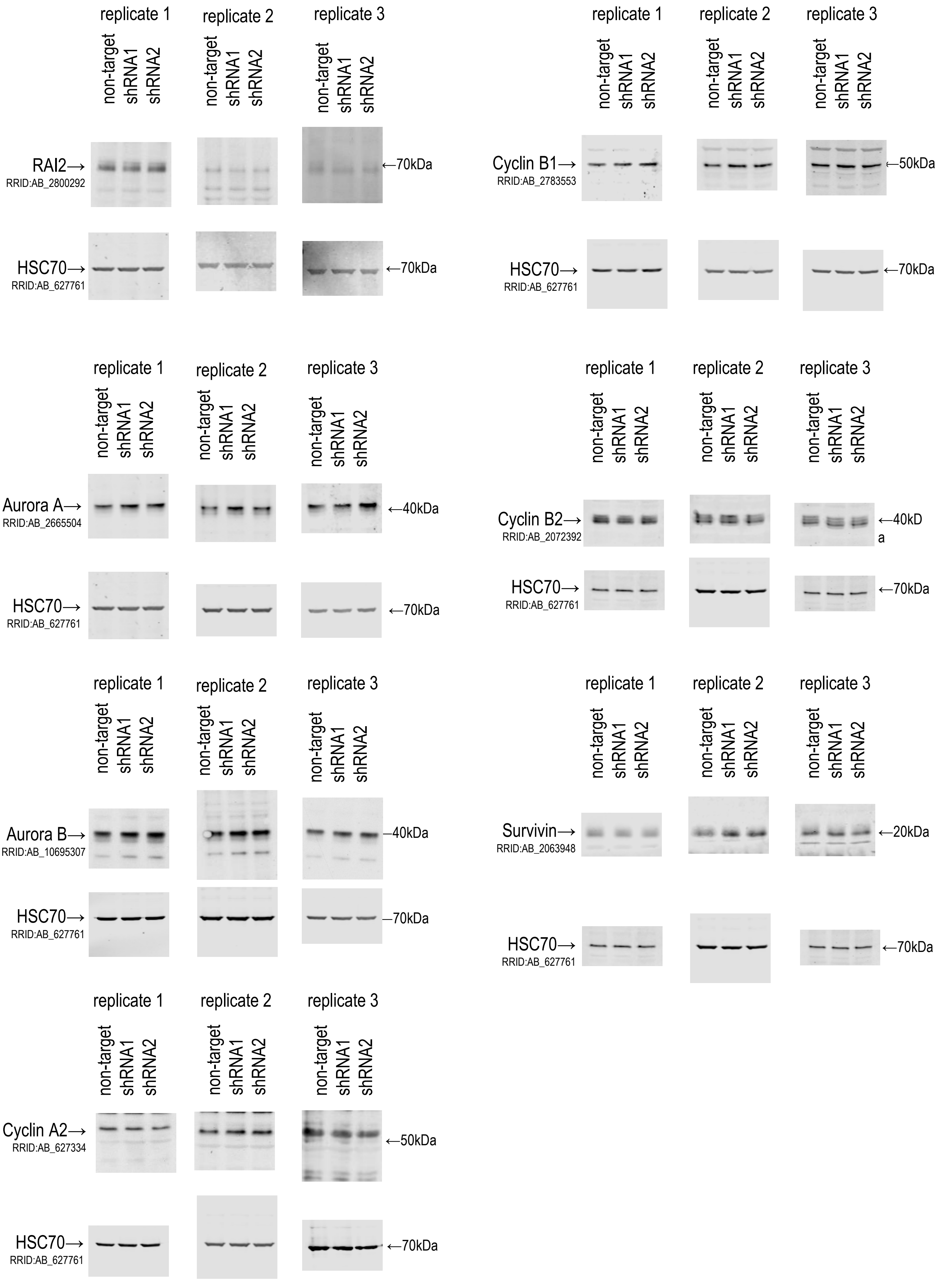

Supplement: Supplementary file 19 — Supplementary Material 19 [file 13058_2025_2085_MOESM19_ESM.pdf]
