# Supplementary material for: Retinoic acid-induced 2 deficiency impairs genomic stability in breast cancer
Source: Breast Cancer Res. 2025 Jul 22;27:137. doi: 10.1186/s13058-025-02085-8 (PMC12285165; doi:10.1186/s13058-025-02085-8)

Supplementary Figure S5: Analysis of **(A)** cell cycle distribution, **(B)** fraction of mitotic cells positive for P-H3(S10), and **(C)** cell proliferation in cell populations of KPL-1, MCF-7 and CAMA-1 cells expressing *RAI2*-specific shRNAs

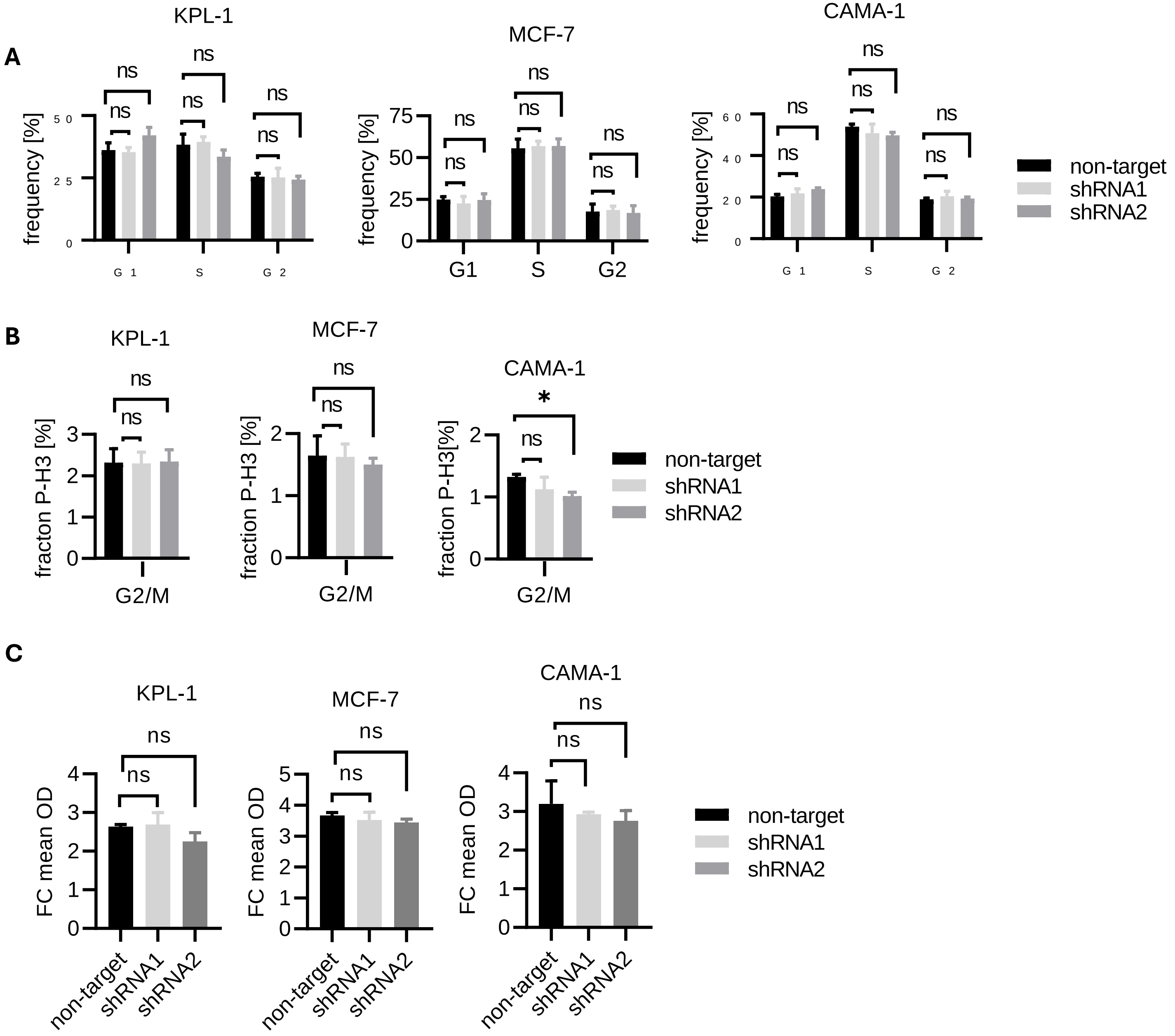

Supplement: Supplementary file 12 — Supplementary Material 12 [file 13058_2025_2085_MOESM12_ESM.pdf]
